# Mode of action-based risk assessment of genotoxic carcinogens

**DOI:** 10.1007/s00204-020-02733-2

**Published:** 2020-06-15

**Authors:** Andrea Hartwig, Michael Arand, Bernd Epe, Sabine Guth, Gunnar Jahnke, Alfonso Lampen, Hans-Jörg Martus, Bernhard Monien, Ivonne M. C. M. Rietjens, Simone Schmitz-Spanke, Gerlinde Schriever-Schwemmer, Pablo Steinberg, Gerhard Eisenbrand

**Affiliations:** 1 Department of Food Chemistry and Toxicology, Institute of Applied Biosciences (IAB), Karlsruhe Institute of Technology (KIT), Adenauerring 20a, 76131 Karlsruhe, Germany; 2 Institute of Pharmacology and Toxicology, University of Zurich, 8057 Zurich, Switzerland; 3 Institute of Pharmacy and Biochemistry, University of Mainz, 55099 Mainz, Germany; 4 Department of Toxicology, IfADo-Leibniz Research Centre for Working Environment and Human Factors, TU Dortmund, Ardeystr. 67, 44139 Dortmund, Germany; 5 Department of Food Safety, German Federal Institute for Risk Assessment (BfR), 10589 Berlin, Germany; 6 Novartis Institutes for BioMedical Research, 4002 Basel, Switzerland; 7 Division of Toxicology, Wageningen University, Stippeneng 4, 6708 WE Wageningen, The Netherlands; 8 Institute and Outpatient Clinic of Occupational, Social and Environmental Medicine, University of Erlangen-Nuremberg, Henkestr. 9-11, 91054 Erlangen, Germany; 9 Max Rubner-Institut, Federal Research Institute of Nutrition and Food, Haid-und-Neu-Str. 9, 76131 Karlsruhe, Germany; 10 Retired Senior Professor for Food Chemistry and Toxicology, Kühler Grund 48/1, 69126 Heidelberg, Germany

**Keywords:** Genotoxicity, Carcinogens, Mutagens, Risk assessment, Mode of action, Endogenous exposure, Exogenous exposure, Toxicogenomics, Biomarker dosimetry

## Abstract

The risk assessment of chemical carcinogens is one major task in toxicology. Even though exposure has been mitigated effectively during the last decades, low levels of carcinogenic substances in food and at the workplace are still present and often not completely avoidable. The distinction between genotoxic and non-genotoxic carcinogens has traditionally been regarded as particularly relevant for risk assessment, with the assumption of the existence of no-effect concentrations (threshold levels) in case of the latter group. In contrast, genotoxic carcinogens, their metabolic precursors and DNA reactive metabolites are considered to represent risk factors at all concentrations since even one or a few DNA lesions may in principle result in mutations and, thus, increase tumour risk. Within the current document, an updated risk evaluation for genotoxic carcinogens is proposed, based on mechanistic knowledge regarding the substance (group) under investigation, and taking into account recent improvements in analytical techniques used to quantify DNA lesions and mutations as well as “omics” approaches. Furthermore, wherever possible and appropriate, special attention is given to the integration of background levels of the same or comparable DNA lesions. Within part A, fundamental considerations highlight the terms hazard and risk with respect to DNA reactivity of genotoxic agents, as compared to non-genotoxic agents. Also, current methodologies used in genetic toxicology as well as in dosimetry of exposure are described. Special focus is given on the elucidation of modes of action (MOA) and on the relation between DNA damage and cancer risk. Part B addresses specific examples of genotoxic carcinogens, including those humans are exposed to exogenously and endogenously, such as formaldehyde, acetaldehyde and the corresponding alcohols as well as some alkylating agents, ethylene oxide, and acrylamide, but also examples resulting from exogenous sources like aflatoxin B_1_, allylalkoxybenzenes, 2-amino-3,8-dimethylimidazo[4,5-f] quinoxaline (MeIQx), benzo[*a*]pyrene and pyrrolizidine alkaloids. Additionally, special attention is given to some carcinogenic metal compounds, which are considered indirect genotoxins, by accelerating mutagenicity via interactions with the cellular response to DNA damage even at low exposure conditions. Part C finally encompasses conclusions and perspectives, suggesting a refined strategy for the assessment of the carcinogenic risk associated with an exposure to genotoxic compounds and addressing research needs.

## Preamble

The risk assessment of carcinogenic substances in food and at the workplace requires sustained scientific evaluation. These substances are usually ingested at trace concentrations through food and are often not completely avoidable at the workplace. Therefore, a working group consisting of members of the SKLM (Senate Commission on Food Safety) and MAK (Senate Commission for the Investigation of Health Hazards of Chemical Compounds in the Work Area) commissions of the German Research Foundation (Deutsche Forschungsgemeinschaft, DFG) was established to delineate a refined risk assessment of chemical carcinogens, based on their modes of action.

The document is structured into an introductory part, followed by three main parts A, B and C.

Part A "Fundamental considerations" develops fundamental considerations highlighting the terms hazard and risk with respect to DNA reactivity of genotoxic agents, as compared to non-genotoxic agents. It further discusses biological consequences and the lessons for risk assessment. This is complemented by an in-depth analysis of the relation between DNA damage and cancer risk. These fundamental considerations are seconded by a more detailed description of current methodologies used in genetic toxicology, in dosimetry of exposure and for the elucidation of modes of action (MOA). Finally, a major consideration is given to the endogenous exposure and its association with background DNA damage in animal and human tissues/cells and its potential relevance for human health risk assessment.

Part B "Selected examples" addresses a series of specific examples of genotoxic carcinogens humans are exposed to as a result of their living conditions, encompassing working place associated exposures as well as those contributed exogenously from their environment and their consumption habits. In addition, endogenous exposure to such agents is taken into consideration, tracing back to endogenous energy metabolism. The latter aspect is reflected by a group of agents put together under the term “the aggregate exogenous and endogenous exposome”. It encompasses compounds humans are exposed to exogenously and endogenously, such as formaldehyde, acetaldehyde and the corresponding alcohols as well as some alkylating agents, ethylene oxide, and acrylamide (Sec. “Acetaldehyde and Ethanol” to Sec. “Formaldehyde”).

The second section of part B addresses compounds humans are exclusively exposed to by exogenous exposure. The third section of part B is devoted to carcinogenic metal compounds with special emphasis on cadmium and arsenic.

Part C "Conclusions and Perspectives" represents the final part of the document, encompassing conclusions and perspectives. This part reiterates the requirement for a MOA-driven risk assessment, exemplified by selected agents where the MOA categories apply and where consideration of an endogenous background is applicable. A flow chart suggesting a strategy for the assessment of the carcinogenic risk associated with an exposure to genotoxic compounds is presented. Furthermore, gaps in knowledge and research needs are discussed at the end.

## Introduction

The distinction between genotoxic and non-genotoxic carcinogens has traditionally been regarded as particularly relevant for risk assessment. For the latter type of agents, which are often classified as “tumour promoters”, independently of diverse underlying mechanisms, the existence of no-effect concentrations or threshold levels is assumed. In contrast, genotoxic carcinogens, their metabolic precursors and DNA reactive metabolites are classically conceived to represent a risk factor at all concentrations because even one or a few DNA lesions, according to the concept of a non-threshold MOA may result in mutations and thus increase the tumour risk. Other substances may increase genomic instability by indirect genotoxic mechanisms, i.e. by not directly reacting with DNA, such as by interference with the cellular response to DNA damage (see part A below), so that again no-effect levels may exist. In the case of the direct genotoxic agents, risk managers have followed for a long time the so-called minimization principle (ALARA: As Low As Reasonably Achievable). From a practical point of view, however, this approach does not adequately take into account the MOA and thus may be overprotective in many cases. As a consequence, the plausibility of the linear-no-threshold hypothesis of cancer risk assessment has increasingly been scrutinized (Cohen et al. 2019; Costantini and Borremans 2019; Doe et al. 2019; EPA 2005; EU 2009; Golden et al. 2019; Greim and Albertini 2015; Kobets et al. 2019; Kobets and Williams 2019; Preston and Williams 2005; Williams et al. 2005; Wolf et al. 2019). For example, threshold levels in experimental animal studies have been postulated for several carcinogens (Kobets and Williams 2019). Since most of the multiple key events in chemical carcinogenesis have a non-linear dose–response relationship intrinsic to the mechanism involved, tumor development will likely show an overall threshold. However, it has to be kept in mind that thresholds for tumorigenicity derived from long term animal experiments necessarily reflect approximations since the biological data observed are prone to many experimental variables and limitations in statistical power. Therefore, within this review, one main focus is given to the quantification of bioindicators of key toxic effects, including the induction of DNA damage, mutations, cell cycle control, enhanced cell proliferation and apoptosis. As detailed below, recent advancements in analytical and cell molecular technologies have markedly contributed to a better-informed risk evaluation. This applies for instance to the detection and quantification of DNA lesions and mutations but also to transcriptomic and other cellular responses, all contributing to a deeper mechanistic understanding of the key processes governing the respective adverse outcome. Scientific experience tells that the dose–response relationship for genotoxic carcinogens in the low dose range and thus the existence of an apparent or true threshold is substance-specific and inevitably depends on the biological effect considered and on the specific MOA(s) that are key for a given adverse outcome (in this case malignant transformation).

Within this manuscript, special attention is also given to background levels of DNA damage of selected substances, arising from endogenous and/or exogenous sources. It proposes, where applicable, to take this “physiological” background more into account when undertaking a risk assessment. These background levels are amenable to quantification with utmost sensitivity and specificity and can be used as reference values against which to compare effects on the integrity of DNA potentially associated with low dose human exposure to genotoxic agents.

This strategy has been propagated already previously, e.g. for risk assessment of formaldehyde exposure (Clewell et al. 2019; Farland et al. 2019). Taken together, instead of classical default linear extrapolation to a lifetime cancer risk level (e.g. 1/ 10^6^), an alternative, mechanistic data-based approach of risk assessment is proposed.

**Fig. 1:**
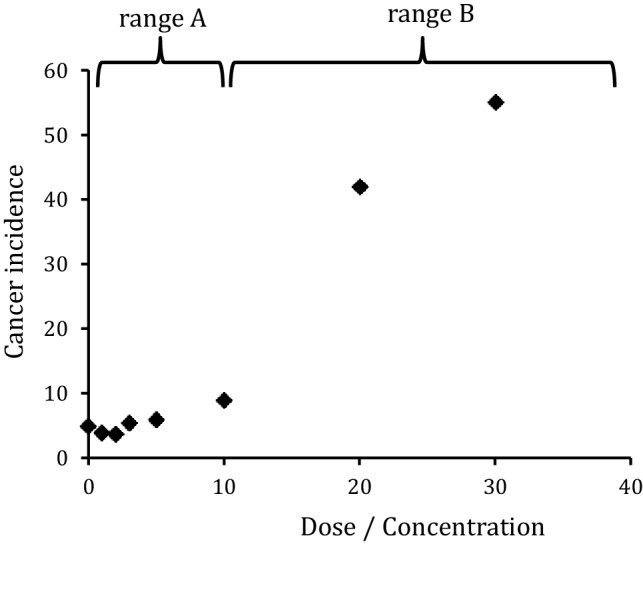
Schematic graph of an apparently non-linear dose–response as observed in many cancer risk studies. In a low dose range (“A”), in which promotional effects are absent and detoxification and repair mechanisms fully active, the cancer risk of genotoxic carcinogens is often too low to be quantified directly. A measurable (often steep) increase of the cancer incidence is observed in the high dose range (“B”) due to the onset of tumour promotion and/or saturation effects. If the slope of this range is used for risk assessment by (linear) extrapolation, the cancer risk in range “A” is likely to be overestimated.

For many carcinogens (both genotoxic and non-genotoxic), the dose–response curves are not linear across the entire dose range. In fact, they often are at least biphasic, in the sense that a smooth (sometimes nearly flat) range is followed by a steep increase, as shown in Fig. 1. While the slope of range A is determined by the induction of DNA damage and its conversion into mutations under conditions where no additional promotional mechanisms are active, the steep increase in range B can be mechanistically explained by the saturation of detoxifying or repair mechanisms and/or by the induction of any type of tumour promotion mechanisms, which generally follow a non-linear (often threshold-like) dose-/concentration-dependent response.

Toxicological risk assessment is particularly interested in the determination of the slope in the low dose range (range A in Fig. 1). Obviously, this slope is not accurately accessible by the direct measurements of dose-dependent cancer incidences, neither in animal studies nor in human population studies, since incidences of 1:1000 up to 1:100,000 are highly relevant for human risk evaluation, but out of scale in the experimental settings. Furthermore, this slope is also not accessible by extrapolation from the high-dose range (range B in Fig. 1) even when benchmark dose (BMD) methods or the calculation of a point of departure (POD) from the BMD is used (“top-down” models).

For theoretical reasons, the concentration dependence at very low doses is expected to be linear, since both chemical and physico-chemical reaction rates become pseudo-first-order with respect to a reactant if its concentration is low enough. This may apply to adduct formation rates, detoxification rates, bioactivation rates and other factors, which ultimately determine the rate of cancer formation. The real slope can also be very close to zero in this low dose range. In practice, experimental data points in the low dose range often will result in a calculated slope that is statistically not significantly different from zero. This does not mean that the actual risk is zero or that the data are useless for risk assessment. Actually, they can be used to calculate a statistical “maximum slope” in the low dose range and thereby a maximum value for the cancer risk for a given exposure.

How can toxicological risk assessment deal with this situation? Three different concepts can be distinguished, as outlined below.

1. The linear extrapolation of the experimentally accessible dose–response data (generally from range B in Fig. 1) may be taken as a worst-case scenario. In the “margin of exposure” (MOE) approach the whole dose–response range is considered and submitted to mathematical modelling. The best-fitting model (or more conservatively the model average) is selected to define a benchmark response (BMR, usually 10% = BMD_10_, but a lower value may be used in certain cases). The lower bound of the confidence limit of the dose associated with the selected BMR, the BMDL, is used as a POD to identify exposures of concern. The MOE then is defined as the dose giving 10% extra risk above background level (BMDL_10_) divided by the estimated daily intake (EDI). If the commonly observed average exposure range in the population is at least 10,000-fold lower than the BMDL_10_, this is equivalent to a MOE of > 10,000. For such a MOE, no serious health concerns or no priority for risk management measures are concluded (EFSA 2009b). The MOE approach has been used by the European Food Safety Authority (EFSA) (EFSA 2005) e.g. to assess exposure to naturally occurring food constituents such as estragole or methyleugenol as well as to certain process-related food contaminants such as acrylamide and furan (EFSA 2015, 2017a). Alternatively, linear extrapolation (sometimes over several orders of magnitude) can be used to define a so-called virtually safe dose (VSD), which is the dose estimated to be associated with an additional risk of one cancer in a million above background levels upon life-time exposure for the general population. For workplace exposure, respective “accepted” risk levels are not generally agreed on, but are usually higher as compared to those for the general population. Thus, one concept applied in Germany by the Committee on Hazardous Substances (Ausschuss für Gefahrstoffe, AGS) consists in calculating a tolerable (4:1000) or acceptable risk (4:10,000 down to 4:100,000) by extrapolation from epidemiological data or from carcinogenicity studies.
2. The “mode of action” (MOA) concept is based on the understanding that experimental data for cancer risk calculations very often result from a dose range at which the induction of non-linear “tumour promotion” mechanisms (e.g. accelerated cell division due to irritation or inflammation) or the saturation of defence mechanisms are likely to play an important role. Thus, the data reflect range B of a dose–response curve as shown in Fig. 1. In all these cases, the type and dose-dependence of the supposed relevant non-linear, promotional mechanisms can be investigated in independent experiments, and the results can be exploited to estimate the lower end of range B in Fig. 1. This lower end of range B then will allow a calculation of a “maximum slope” for the (linear) dose–response curve in the (experimentally not accessible) low dose range A. The German MAK Commission initiated MOA based risk assessments in 1998 by the introduction of the categories 4 and 5 for those carcinogenic substances, for which a MAK (maximum workplace concentration) value can be derived. Category 4 includes substances that cause cancer in humans or animals, where a non-genotoxic MOA is of prime importance for the observed carcinogenicity, and genotoxic effects play no or at most a minor part provided the MAK and BAT (biological tolerance) values are complied with. Under these conditions, no contribution to human cancer risk is expected. Category 5 applies to substances that cause cancer in humans or animals, for which a genotoxic MOA is of prime importance, but is considered to contribute only very slightly to human cancer risk, provided the MAK and BAT values are observed. Both categories require a detailed understanding on the MOA of the respective substances; for category 5, the cancer risk at the low dose level must be quantifiable and considered to be low. The relevant detoxification, repair and “damage response” mechanisms for these MOA considerations as well as respective test systems and a toxicogenomics approach for their identification are discussed in more detail below (see part A "Fundamental considerations", chapters "DNA reactivity of chemicals: Hazard versus risk", "Protein adducts as human biomarkers", "Background DNA lesions in rodent and human tissues/body fluids").
3. Further carcinogenesis-related endpoints, in particular the frequency of DNA adducts and mutations as well as surrogate endpoints such as protein adduct levels, might be used to extend the experimentally accessible exposure range for effect measurements thereby aiming at better risk estimates for the low dose range. Methodological progress, e.g. in the quantification of DNA adduct levels, has stimulated research in this area. In particular, endogenous background levels of DNA adducts have become available in some cases and may allow to calculate a risk increment caused by the exposure to exogenous carcinogens. Although in its infancy, this approach appears promising, and may potentially pave the way to a more comprehensive evaluation of human health risk associated with aggregate exposures to the so-called *exposome,* designating, in this case, the whole array of electrophilic compounds of relevance to human exposure from both exogenous and endogenous sources. Moreover, with respect to a workplace exposure, reference values such as the BAR (Biological Reference Value) in Germany and the BGV (Biological Guidance Value) of SCOEL (Scientific Committee on Occupational Exposure Limits) referring to background levels of a given substance or its metabolite in the occupationally unexposed population are increasingly being defined and can thus be used for evaluation of occupational exposure if exceeded. The potential scope and limitations of these approaches are described below in more detail (see part A "Fundamental considerations", chapters "Toxicogenomics for hazard identification and risk assessment").

In this review, we will describe the mechanistic background of the various concepts and the ways in which they can be applied for a toxicological risk assessment, both in general terms (see part A "Fundamental considerations") and in the case of selected examples (see part B "Selected examples"). Points to be considered when wanting to refine the risk assessment of chemical carcinogens in food and at the workplace are outlined in part C "Conclusions and Perspectives".

## Part A: Fundamental considerations

### DNA reactivity of chemicals: hazard versus risk

**Fig. 2:**
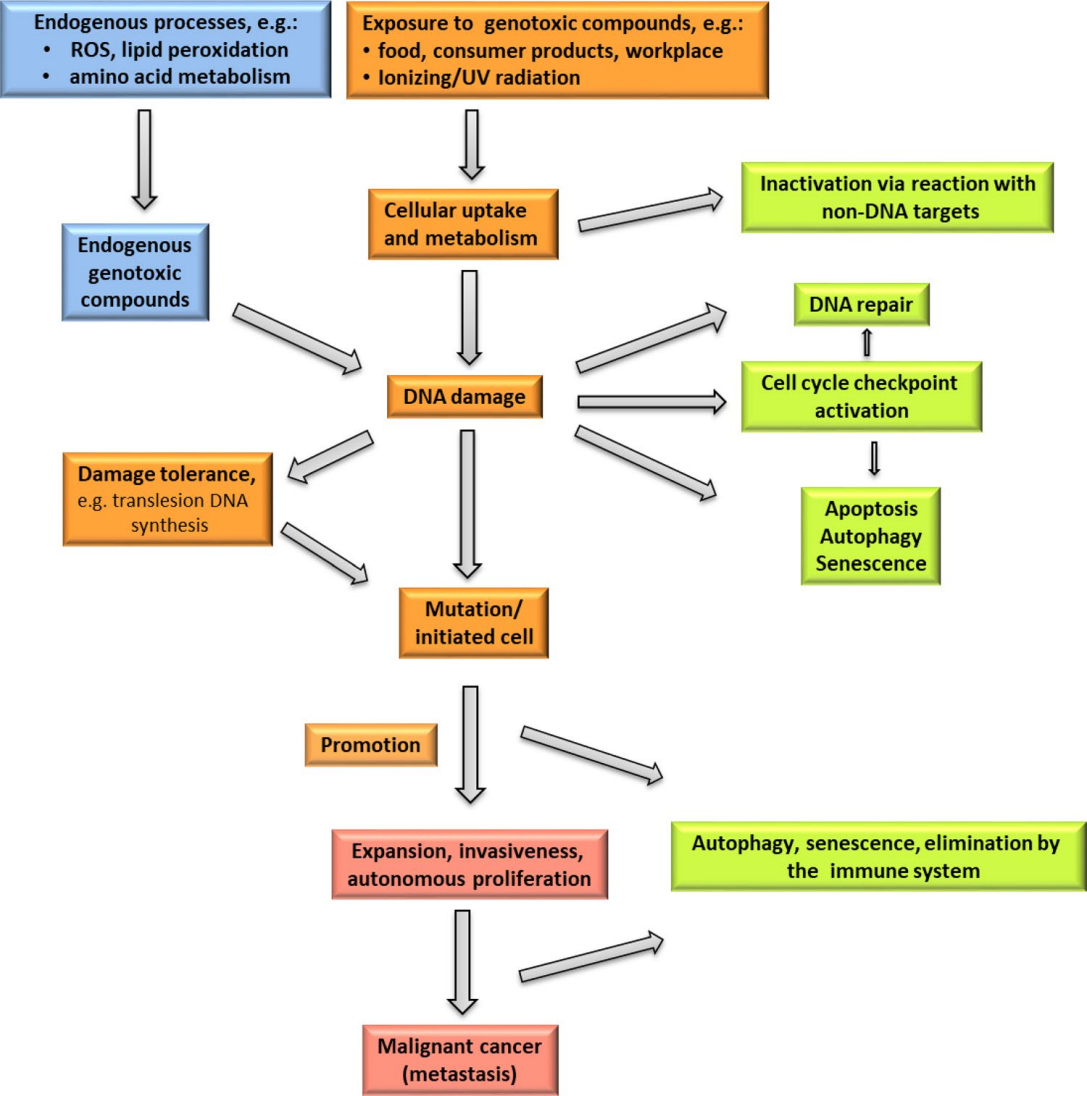
Schematic outline of causes and consequences of DNA damage (partly proposed previously by Thomas et al. 2015) *Left* Endogenous and exogenous factors and cellular processes leading to DNA damage and increasing the risk of tumour development. *Right* Processes decreasing the extent of DNA damage, mutation induction and tumour development

The process of carcinogenesis is outlined in Fig. 2. Every single step is expected to follow its own (often non-linear) dose-dependence and kinetics. It is important to note that environmental/xenobiotic substances are not only able to trigger the process at its origin by causing DNA damage, but potentially can influence all relevant steps and pathways shown in Fig. 2, either directly or indirectly, for example by inhibiting specific steps or by inducing an adaptive response. In the following paragraphs, some of the steps are discussed in greater detail, in particular with respect to their impact on risk assessment and risk evaluation.

The mutagenicity of chemical or physical agents is frequently assessed by short-term mutagenicity assays either in bacteria (Ames-test) or in cultured mammalian cells. These test systems are designed to detect the ability of these agents to damage DNA and thus lead to mutations, yielding important hints for hazard identification. However, in the frame of the risk assessment process, informed judgement is required in terms of relevance for human health. This also implies that positive findings in these in vitro models need follow-up in in vivo models before a definite conclusion on their in vivo genotoxicity can be reached.

#### From exposure to DNA damage

There are several scenarios that would prevent the induction of significant levels of DNA damage by a potential mutagen in humans

- A substance is not absorbed in the gastrointestinal tract and is thus excreted unchanged. However, even though this would not lead to systemic bioavailability, this may lead to an exposure of the epithelium lining of the gastrointestinal tract.
- A substance or its metabolite may be so reactive that it preferentially reacts with proteins and/or other nucleophiles within cells or tissues, thereby not reaching the target tissue DNA in relevant concentrations or only reaching the upper cell layers and not the proliferative basal cells in a particular tissue (see part B "Selected examples", Sec. “Formaldehyde”).
- There may be effective detoxification mechanisms, such as conjugation with glutathione (GSH) or epoxide hydrolysis and other phase II metabolic processes, which scavenge the proximate/ultimate electrophiles and thus limit their availability for DNA interaction.

Whether or not significant levels of DNA damage are induced under defined exposure conditions needs to be determined by mechanistic in vitro*/*in vivo experiments and/or biomarker studies. Furthermore, if arising from the same reactive metabolite(s), following similar kinetics, protein adducts may be utilized as surrogate markers for DNA damage.

In addition, several DNA reactive metabolites also arise endogenously, e.g., during physiologic carbohydrate, lipid and amino acid metabolism. Examples are formaldehyde, acetaldehyde, ethylene oxide, acrylamide and certain alkenals generated from lipid peroxidation (see part B Sec. “Presence of endogenous background levels of the same or similar DNA lesions”). Furthermore, DNA is continuously damaged by endogenous processes, such as the generation of reactive oxygen radicals (ROS) and other electrophiles, in part due to leakage from the electron transport chain operative in cellular respiration and to electrophile leakage from physiological metabolism (Hakem 2008; Sharma et al. 2016; Valko et al. 2006). It thus becomes mandatory to assess the increment in health risk associated with exogenous exposure to genotoxic compounds from food, consumer products or workplace-exposure in relation to the natural variance of endogenous DNA damage.

Finally, the cell has quite efficient DNA repair systems, which may prevent the conversion of DNA lesions into mutations. In this context, it has been postulated that there is a general level of DNA lesions conceived not to be relevant for mutations, due to efficient repair (EFSA 2005; Jenkins et al. 2010). Nevertheless, whether or not this general assumption of comprehensive repair in the low dose range holds true for all types of DNA lesions will be discussed below, both theoretically (in part A "Fundamental considerations") and by considering different genotoxic substances (in part B "Selected examples").

#### From DNA damage to mutations

The maintenance of intact genetic information is essential for basically all cellular processes and for the prevention of tumour development. However, many environmental agents as well as genotoxic carcinogens/mutagens in food or at workplaces may compromise genetic stability by inducing different types of DNA lesions (see part B "Selected examples" for examples and details). DNA damage interferes with DNA transcription and replication; potential consequences are programmed cell death, mutagenesis and genomic instability, which may lead to cancer when occurring in somatic cells, but also to reproductive toxicity when affecting sperm or egg cells. To maintain the integrity of the genome and to keep the mutation rate low, a complex DNA damage response network has evolved (Camenisch and Naegeli 2009; Christmann et al. 2003; Fousteri and Mullenders 2008; Roos et al. 2016). It includes diverse DNA repair systems for different types of DNA lesions, cell cycle control mechanisms to prevent replication of damaged DNA as well as the induction of apoptosis in case of heavily damaged DNA. Mutations arise by direct integration of incorrect DNA bases in the course of replication or by adaptive mechanisms, depending on the type of DNA lesion. One example for the direct induction of mutations is the presence of apurinic/apyrimidinic (AP) or abasic sites, where the base is missing and, therefore, the correct base pairing information. Further examples are methylated bases such as *O*^6^-methylguanine, which may induce direct miscoding during DNA replication. In case of bulky DNA lesions, replication on a damaged DNA template is prevented by the high fidelity of replicative polymerase delta and associated proofreading factors. Nevertheless, adaptive mechanisms allow for completion of DNA replication and thus for cell survival in spite of low levels of DNA damage. Besides homologous recombination, error-tolerating polymerases may be activated; this adaptation operates–depending on the type of DNA lesion–at the expense of genomic stability due to enhanced mutation rates (e.g. Thomas et al. 2015) (summarized in Fig. 2).

#### DNA repair systems

**Fig. 3:**
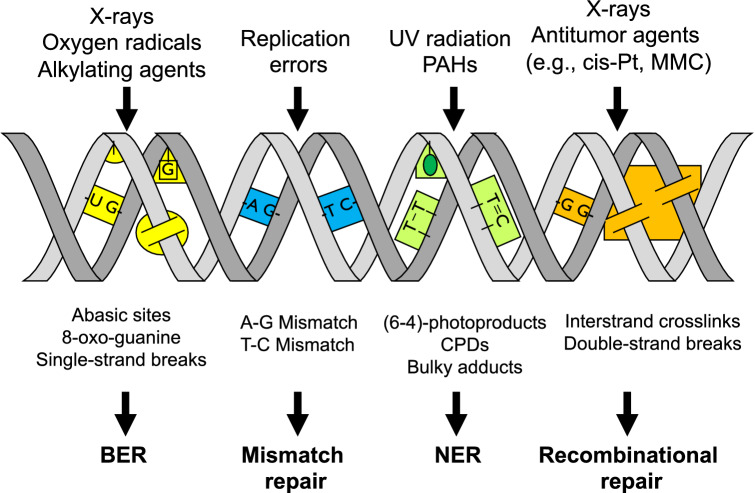
Major causes of DNA damage and DNA repair pathways (adapted from de Laat et al. 1999); *BER* base excision repair, *NER* nucleotide excision repair, *CPD* cyclobutane-pyrimidine dimer *cis-Pt* cisplatin, *MMC* mitomycin C

As indicated above, DNA repair systems are most important to largely prevent the conversion of DNA lesions into mutations, i.e. fixed changes of genetic information. In mammals, there are only a few examples for direct DNA damage reversal, such as the transfer of the methyl group from *O*^6^-methylguanine to the methylguanine methyltransferase (MGMT) and the oxidative dealkylation of 3-methylcytosine and 1-methyladenine by the human homolog of the bacterial AlkB, ABH2. In other cases, several different DNA repair pathways become selectively activated depending on the type of DNA damage (Fig. 3).

##### Nucleotide excision repair (NER)

NER is the most versatile repair system involved in the removal of structurally unrelated bulky base adducts, which cause significant helical distortions. It can be subdivided into global genome repair (GG-NER) and transcription-coupled nucleotide excision repair (TC-NER), which preferentially removes transcription-blocking bulky DNA lesions. At least 30 different proteins and enzymes are required in mammalian cells, including those which are defective in patients suffering from the DNA repair disorder Xeroderma Pigmentosum (XP) complementation groups A through G. The most crucial step is the damage recognition, followed by the incision at both sides of the lesion and the repair polymerisation leading to the displacement of the damaged oligonucleotide. Repair is completed by the ligation of the repair patch. Well investigated substrates are UVC-induced pyrimidine dimers, but also DNA lesions induced by genotoxic chemicals such as benzo[*a*]pyrene and others (for reviews see e.g. Camenisch and Naegeli 2009; Christmann et al. 2003; de Boer and Hoeijmakers 2000; Fousteri and Mullenders 2008; Marteijn et al. 2014). With respect to genomic stability, NER is an error-free process, as long as only one strand of the DNA double helix is affected. The correct information is located on the intact strand and can be copied during repair replication. However, due to the heterogeneity of DNA repair, largely depending on the accessibility of the lesions in regions with different degrees of chromatin condensation, nucleotide excision repair is comparatively slow and may not be completed, especially in heterochromatic regions, before the cell divides (Feng et al. 2003; Mullenders et al. 1991).

##### Base excision repair (BER)

In contrast to NER, which detects a rather broad spectrum of DNA lesions, BER is initiated by a specific class of DNA repair enzymes called glycosylases, which specifically act on one or few substrates. BER is mainly responsible for the removal of different types of DNA lesions that are also generated by endogenous processes, including oxidative DNA base modifications like 8-hydroxy-deoxyguanosine (8-oxo-dG) or DNA alkylation, most frequently at the N7-position of guanine. The first step of BER generates AP sites, which are further processed in a multistep procedure with slight differences depending on the type of damage (Camenisch and Naegeli 2009; Christmann et al. 2003; de Boer and Hoeijmakers 2000; Hakem 2008; Kennedy et al. 2018). Compared to NER, lesions are repaired faster and also error-free; nevertheless, the occurrence of AP at the time of replication may be premutagenic.

##### Mismatch repair (MMR)

Another DNA repair system of particular relevance for maintaining genomic stability is MMR. This evolutionary conserved pathway is responsible for the repair of mismatched normal bases after DNA replication, contributing significantly to the extraordinary fidelity of DNA replication. Cells deficient in MMR exhibit a “mutator phenotype”, in which the rate of spontaneous mutations is greatly elevated, and defects in MMR are associated with an increased risk of different types of cancer. The MMR system also plays a key role in cell killing in response to alkylating agents, and MMR-deficient cells are about 100 times more resistant to the cytotoxic effects of alkylating agents (Gupta and Heinen 2019; Hsieh and Yamane 2008; Liu et al. 2017; O'Brien and Brown 2006).

##### DNA double-strand break repair

DNA double-strand breaks (DSB) are induced by exogenous agents, including ionizing radiation, DNA crosslinking agents such as mitomycin C, and topoisomerase inhibitors, but also by endogenous processes, for example, ROS formation, replication on a damaged DNA template and meiotic recombination. They represent most critical DNA lesions since both DNA strands are affected. If not repaired, they lead to loss of large chromosomal regions and cell death. Broadly, two major principles of DSB repair can be discriminated, namely non-homologous end-joining (NHEJ), which does not require sequence homology, and homologous recombination (HR) which uses sister-chromatids as homologous template to copy and restore the DNA sequence missing on the damaged chromatid. While NHEJ is active throughout the cell cycle, HR is restricted to the S- and G2-phase of the cell cycle; however, even in G2 NHEJ is the most active mode of DSB repair. Both types of DNA repair pathways also exert different impacts on genomic stability: While NHEJ protects from cytotoxicity, it is highly error-prone, and thus a pro-mutagenic process. In contrast, HR is largely error-free. Finally, microhomology-mediated end-joining (MMEJ) and single-strand annealing (SSA) may occur, for example in the case of defects in HR. DSB repair deficiencies are associated in different human disease syndromes with increased cancer susceptibility, neurological abnormalities and immunodeficiency, including Ataxia telangiectasia and the Nijmegen Breakage syndrome. With regard to genomic instability, for example, women carrying mutations in the BRCA1/BRCA2 (breast cancer 1/2) genes resulting in HR deficiency exert increased cancer risk (for reviews see Aparicio et al. 2014; Bonetti et al. 2018; Chang et al. 2017).

#### Tumour suppressor functions

Besides DNA repair systems, further DNA damage responses are activated upon genotoxic stress in mammalian cells (Fig. 2). They include cell cycle control mechanisms, increasing the time for DNA repair, as well as apoptosis, thereby eliminating heavily damaged cells. The DNA damage response is strictly coordinated, for example, by the tumour suppressor protein p53. p53 regulates cell cycle control and apoptosis by several coordinated pathways and thus exerts a pronounced impact on the processing of DNA damage and on genomic stability (Hainaut and Hollstein 2000).

#### Consequences for chemical risk assessment

Taken together, the induction of DNA lesions and the fixation of mutations are separate processes, discriminated by mechanisms and kinetics. While the former may be repaired by the DNA damage response system, mutations are fixed changes in nucleotide sequence, which may or may not affect protein function. Nevertheless, the DNA damage response has to be recognized as a double-edged sword. While DNA repair systems are largely error-free, persisting DNA lesions may be tolerated at the expense of generating mutations.

With respect to the risk assessment of chemicals, DNA repair systems are protective by removing many types of lesions by base or nucleotide excision repair. However, several chemicals have been shown to interfere with distinct steps of DNA repair pathways and, thus, may lead to relevant effects, similar to the increased genomic instability in repair-defective cells. In some cases, the disturbance of DNA repair systems has been observed at very low compound concentrations, which are relevant even under environmental exposure conditions, with arsenic and its metabolites as one prominent example (Hartwig 2013b) (see also part B "Selected examples"", Sec. “Carcinogenic metal compounds: Examples cadmium and arsenic”). In addition to DNA repair systems, cell cycle control and apoptosis also have to be considered as protective mechanisms in case of heavily damaged DNA, and, consequently, agents that interfere with the cell cycle control or inhibit apoptosis may increase genomic instability and thus cancer risk. Finally, all events that promote cell division and growth, either via the induction of specific signalling pathways or via cytotoxicity or inflammation with subsequently accelerated cell proliferation, may indirectly impair genomic stability by decreasing the time window for repair and thus promoting replication of damaged DNA templates. Even though the interactions with the DNA damage response system are based on protein interactions, and may, therefore, follow non-linear dose–response relationships, safe concentration ranges need to be defined to prevent the corresponding interactions (Langie et al. 2015).

#### From mutations to cancer

Based on the current understanding of tumour development, DNA damage and mutations are key events in carcinogenicity. Mutations that arise in critical genes like DNA repair genes increase the probability of further mutations in proto-oncogenes and tumour suppressor genes or their regulatory sequences, leading to a gradual increase in genomic instability (“mutator phenotype”) (Loeb 1991). At the cellular level, characteristic changes associated with malignant transformation can be observed. Thus, the “Hallmarks of Cancer” originally described by Hanahan and Weinberg (Hanahan and Weinberg 2000) comprise “sustaining proliferative signalling”, “evading growth suppressors”, “resisting cell death”, “inducing angiogenesis”, “replicative immortality” as well as “activating invasion and metastasis” (Hanahan and Weinberg 2000). They were complemented in 2011 by two additional hallmarks “deregulating cellular energetics” and “avoiding immune destruction”, with the consequences of “tumour-promoting inflammation” and “genome instability and mutation” (Hanahan and Weinberg 2011). These cellular biological changes become evident in late stages of carcinogenesis and may occur as a result of numerous selection processes in the tumour tissue. Furthermore, epigenetic alterations of DNA are also associated with tumour development. For example, hypo- or hypermethylation of promoter regions of critical genes may lead to the overexpression of oncogenes or the suppression of tumour suppressor genes, and exposure towards some chemical carcinogens such as certain metal compounds may lead to changes in the methylation patterns. Moreover, interference with histone acetylation and deacetylation may also interfere with gene expression.

### Test systems in genetic toxicology

#### General considerations

In the case of tumour initiation, in which genotoxicity is critically involved, tumour suppressor gene inactivation or oncogene activation is the decisive molecular event. However, up to now, these biologically meaningful genes do not lend themselves to a technically feasible analysis of mutation frequencies. Thus, mainly for practical reasons, surrogate genes are used to assess mutation frequency, and it is assumed that mutation induction is basically comparable across different parts of the genome. Whereas this certainly is a generalization, it is a valid assumption that whenever mutations or other types of genetic damage are induced in a gene used for mutation testing, the risk for cancer-relevant genetic lesions will rise as well. Under routine conditions, germline mutagenicity is estimated by read-across from somatic genotoxicity systems, assuming a likewise risk for germ cells if the risk has been identified in somatic tissue unless exposure of germline tissue can be excluded, but without conducting dedicated germline genotoxicity assays (Yauk et al. 2015).

For any test system, two fundamental characteristics need to be considered to define the appropriateness of the approach: (i) the genetic endpoint and (ii) the experimental model. Gene mutations and chromosome damage are used as endpoints in routine testing because they have been recognized as critical molecular events in tumour initiation. In addition, primary DNA lesions or other endpoints can be used to investigate genetic damage but in general are not used routinely. To measure gene mutations, biochemical cell functions such as enzyme activities or membrane receptors are analysed, whereas chromosome aberrations are mainly assessed by microscopic techniques or flow cytometry. Obviously, to investigate chromosomal integrity, eukaryotic systems are needed, whereas gene mutations can also be measured in bacterial cells. In this context, it is important to understand that a spectrum of genetic damage can lead to tumour initiation. For this reason, in most testing settings, a combination of tests is used, with the idea of covering all possible mechanisms that can lead to tumour initiation (MacGregor et al. 2000). Additional considerations of the appropriate applicable testing strategy are derived from the intended use of a chemical, i.e. whether a molecule is used as an industrial chemical, a pesticide or a pharmaceutical ingredient, or whether it is present therein as a component or an impurity, or in food or the environment. Accordingly, the regulatory context and the applicable guidances vary with respect to the rationale underlying the testing strategies and the stringency and scientific depth of the approaches. As an example of a highly regulated field, ICH (International Council for Harmonisation of Technical Requirements for Pharmaceuticals for Human Use) guidance S2 describes the testing approach for novel pharmaceutical ingredients (ICH 2011).

#### Strategies for genotoxicity assessment

According to this guidance, basic genotoxicity assessment consists of a bacterial mutation (*Salmonella typhimurium* reverse mutation) test to investigate the induction of gene mutations in vitro (OECD TG 471) (OECD 1997). In this test, like in all in vitro tests, an extracellular system is used to provide for metabolic activation of pre-mutagens. Routinely, this consists of a preparation from chemically induced rat liver, which contains cytosolic as well as microsomal enzymes plus co-substrates to mainly stimulate oxidative metabolism, to detect those mutagens that need metabolic activation to become DNA-reactive. Obviously, this experimental design is not sufficient to cover all relevant activating conditions but has nonetheless shown to possess a remarkable predictivity for genotoxic carcinogenesis (Kirkland et al. 2005). Moreover, since this test system is both sensitive for mutagens of diverse chemical classes as well as not very prone to artefacts, it is considered a reliable predictor of relevant mutagenicity (Kirkland et al. 2005).

According to ICH M3 (R2), an assay for gene mutation is generally considered sufficient to support all single-dose clinical development trials (ICH 2009). ICH S2 provides two options for genotoxicity testing starting in the first place with the gene mutation assay in bacteria for both options. To support multiple-dose Phase I trials, an additional assessment capable of detecting chromosomal damage in a mammalian system(s) should be completed. A complete battery of tests for genotoxicity should be performed before initiation of Phase II trials. However, if positive findings occur, an assessment and then possibly additional testing is needed.

Option 1 consists furthermore of a mammalian cell test in vitro, which is either a thymidine kinase (tk) gene mutation test in L5178Y mouse lymphoma cells or a chromosome aberration or micronucleus test (MNT) in basically any cell type that is amenable to these endpoints. If positive only under conditions in which human relevance can be convincingly excluded (e.g. excessive cytotoxicity) or negative, this package of two negative tests will allow the initiation of a clinical Phase I study with multiple dosing, and an in vivo test (chromosome aberration or MNT in rodent bone marrow or peripheral blood) is not needed before Phase II clinical studies. If positive, the relevance of the positive result(s) needs to be investigated. Relevance in this context can be demonstrated in various ways. Mostly, if an effect can be excluded under appropriate conditions in animals (e.g. sufficiently high exposure in the absence of target tissue toxicity), this is a strong evidence of the absence of a risk in humans. In the case of a positive effect in the mentioned mammalian tests, the applicable follow-up strategy would consist of a micronucleus or chromosome aberration test in rodent bone marrow or peripheral blood, plus a second test using a second endpoint and tissue. Due to ease of conductance and applicability to a variety of tissues the second endpoint will be in many cases the evaluation of primary DNA damage via the alkaline single cell gel electrophoresis (comet) assay. Tissue selection should be guided by scientific arguments specific to the situation, e.g. the liver if hepatic metabolic activation is suspected, the GI tract as a tissue of high local compound concentration in the case of the oral administration of the chemical, or organs in which toxic effects or drug accumulation have been observed. In the case of a positive effect in the Ames test, transgenic animals, the comet assay, or the Pig-A assay are available to investigate gene mutations in vivo (Kirkland et al. 2019; OECD 2011). In any case, the conditions need to be appropriate to provide arguments to exclude a human risk with sufficient stringency, for example with respect to doses and treatment schedules used. Accumulating experience has allowed ICH S2 to accept the combination of various endpoints into a single study, such as e.g. a micronucleus and comet test in the same animal. Whereas traditionally these tests were conducted under an acute treatment paradigm, it is now also accepted to use a subacute or subchronic treatment, provided a number of criteria are fulfilled to assure sufficient sensitivity, such as e.g. a highest dose level of at least 50% of a potential acute maximum tolerated dose (MTD).

Alternatively, as Option 2, ICH S2 allows the initiation of Phase II clinical trials without conducting a mammalian in vitro test. In this case, two in vivo tests are needed, similar to the situation in which the mammalian in vitro test is positive under Option 1.

Routinely, in drug development, screening tests will precede those tests intended to assess human risk, which allows an earlier prediction of the outcome of those regulatory tests important for the early selection of the right molecules. Furthermore, computer-assisted prediction systems are widely used to predict bacterial mutagenicity and accomplish it with an acceptable precision (Naven et al. 2012). However, for other endpoints they have not matured to the level that would render them reliable enough for routine use. Generally, endpoints and test systems, which resemble those of the regulatory tests to ensure a sufficient predictivity, such as down-scaled versions of the Ames or MNT, are used. If other test systems, such as e.g. high-throughput test systems, are applied, thorough validation studies need to be performed to ensure their predictivity. If equivocal results are obtained in those standard approaches, exploratory methodologies are applied to clarify the situation. While the above examples of test strategies apply to pharmaceutical ingredients, the underlying principles are comparable to those in other areas, in which genotoxicity testing is applied (Eastmond et al. 2009; ECHA 2017; EFSA 2011b). For example, in the food and feed regulation field, as outlined by EFSA (2011b), a stepwise approach is recommended as well. Initially, a basic in vitro testing step will include the Salmonella reverse mutation test and an in vitro MNT in mammalian cells. This approach is more restrictive than for pharmaceuticals, in which case three tests (MNT, mouse lymphoma tk test and chromosome aberrations test) are offered as equivalent alternatives for assessing mammalian genotoxicity in vitro. If a positive result is obtained in vitro, one or several in vivo follow-up tests are recommended, following a similar logic as with e.g. pharmaceuticals, which includes a test using a comparable genetic endpoint as in the positive in vitro test. Similarly, if characteristics such as metabolism preclude a meaningful use of an in vitro test as the first step, the assessment could be done based on the in vivo testing alone. However, an a priori assessment using in vivo data alone, i.e. Option 2 (ICH S2)-like approach in the absence of arguments that would preclude the use of in vitro tests, is not foreseen by EFSA. In any case, for both ICH and EFSA, if the initial in vitro testing is overall negative in adequately conducted tests, this is sufficient to conclude on the absence of a genotoxic potential of the tested material. For EFSA purposes, this may be sufficient as a final conclusion for a chemical, whereas according to ICH an in vivo test will be needed as a final proof. Moreover, routine germline genotoxicity testing is deemed unnecessary under both regulations, and information obtained in the routine tests is considered sufficient to identify a risk in both somatic and reproductive tissue.

In contrast, for industrial chemicals as outlined in the REACH guidance (ECHA 2017), testing requirements are mainly triggered by annual tonnage, i.e. by the amount of the chemical that is marketed per year in the EU. This means that whenever a new level of tonnage is reached, additional tests are asked for. The underlying principle as to why this is feasible is that for chemicals, whenever a risk is identified, exposure to humans during production, transport and handling can be restricted by technical measures if necessary, whereas this is not possible for drugs or food ingredients. Therefore, the purpose of genotoxicity testing of industrial chemicals is mainly to support labelling and protection measures, whereas in the case of drugs, the purpose is for a risk/benefit consideration. Relevant genotoxicity is incompatible with the development and application of pharmaceuticals in most disease indications. In the case of food constituents and/or contaminants risk assessment is the key step. When for food the presence of trace levels of naturally occurring mutagens/carcinogens or those generated during food processing cannot be avoided, risk assessment with due consideration of exposure levels is mandatory.

In all cases, the conductance of all individual routine tests should follow the recommendations as outlined by the applicable OECD guidelines, which are specific for each test and layout test characteristics, which are essential for a meaningful result, such as e.g. the maximum concentration or dose that should be applied and criteria to define these.

While quantitative considerations have been unusual in genotoxicity testing, in carcinogenicity potency assessment it is an established factor to discuss risk determinants. A methodology to compare potencies is the benchmark dose (BMD), defined as the dose that corresponds to a specific change in an adverse response compared to the background. Often, the lower 95% confidence limit of the benchmark dose level (BMDL) is used, and guidance documents have been released e.g. by EPA (https://www.epa.gov/raf/publications/benchmarkdose.htm) or EFSA (2009b, 2017c). However, comparisons of endpoints of varying biological depth (i.e. for example DNA damage, mutation and cancer) are rare, and at the same time problematic due to the variability of potentially confounding influence factors. In addition, in general genotoxicity tests are not designed to derive quantitative data, so that dose–response relationships are normally not worked out in much detail. Similarly, despite the fact that BMD considerations imply quantitative information, carcinogenicity studies are normally lacking a large number of dose levels, mainly due to the enormous effort associated with such an approach, so that detailed quantitative information is often not available.

In an attempt to quantitatively compare different test systems and endpoints, a literature study was performed, using data from the in vivo comet assay, the MNT, and the in vivo transgenic rodent mutation assay (TG). These assays were chosen because they represent different stages of the mutagenicity process, i.e. the comet assay detects single- and double-DNA strand breaks, the MNT detects numerical and structural chromosomal aberrations, and the TG assay detects mutations. Only studies with two or more dose levels were included (Hernandez et al. 2011). Those data were compared to carcinogenicity data from the Gold Carcinogenicity Database, using IARC Class 1–3 carcinogens. In this study, data on 18 compounds tested in all four test systems were available. Of those 18 compounds, 15 had acceptable dose–response data from the MNT and the TG, but only 4 from the comet assay. Quantitative relationships were investigated by comparing BMD_10_ values modelled from the available genotoxicity data (Comet, MNT, and TG) to BMD_10_ values derived from carcinogenicity studies, acknowledging different administration routes between genotoxicity and carcinogenicity studies with a high number of compounds. Interestingly, despite these confounding factors, a relatively good correlation was observed between MNT and TG tests and the carcinogenicity data. The strongest correlation was found between the lowest BMD_10_ from MNT or TG data (i.e., lowest genotoxicity BMD_10_) and the tissue-matched tumour BMD_10_, in that the strongest genotoxins were also the strongest carcinogens and vice versa, which corroborates the importance of these genotoxicity endpoints in predicting tumour initiation.

Thus, while in general a correlation between the potency of each endpoint can be assumed and has been demonstrated, the sensitivity of the test systems has not been systematically compared. Intuitively, a test system that is able to analyse early events in tumour induction should be more sensitive than a test system quantifying later events such as mutations, owing to the fact that many earlier events (e.g. DNA base modifications) are induced to initiate a lower number of the subsequent ones (e.g. mutations), culminating in a relatively low number of tumours per animal and dose group.

However, experimentally, DNA modifications as the first discernible endpoint of DNA damage were not always the most sensitive markers in certain experimental settings, depending on the sensitivity of the analytical method applied. For example, in a study using L5178Y mouse lymphoma cells in vitro, 8-oxo-7,8-dihydro-2-deoxyguanosine (8-oxo-dG) and 1,*N*^6^-etheno-2-deoxyadenosine DNA base modifications were measured by LC-MS/MS after treatment with hydrogen peroxide or cumene hydroperoxide at concentrations of up to 500 µM or 10 µM, respectively (Brink et al. 2009). While at these concentrations no increase in either DNA modification levels were found, DNA damage as assessed in the comet assay as well as the MN and tk gene mutation frequencies were significantly elevated. In case of 8-oxo-dG this may be due to potential artefacts when measuring oxidatively induced DNA lesions by LC-MS/MS resulting from the unintended generation of DNA lesions during sample preparation (Gedik et al. 2005). In contrast, after treatment with methyl methanesulfonate (MMS), the induction of 7-methylguanine (7-MG) or *O*^6^-methyl-2′-deoxyguanosine (*O*^6^-mdGuo) adducts occurred at a much lower concentration than those inducing the other genotoxicity endpoints. Moreover, the authors caution that other factors may have contributed to this unexpected result. As such, it is only those two adducts that were determined but it is conceivable that other adducts or mechanisms not involving adduct formation are more relevant regarding the mutations induced. Furthermore, no detailed time courses were chosen for the various endpoints, so that optimal readouts may not have been obtained. Thus, based on these results, it was concluded that DNA adduct formation, like other endpoints, needs to be analysed on a case-by-case basis, using optimized conditions and that there is no general correlation between DNA adduct levels at a given time point and the subsequent mutations.

Finally, novel test systems have been described to refine the available approaches. Among the most advanced, the Pig-A assay offers a promising potential. The assay is based on the cytometric visualization and quantitation of markers linked to cell surfaces via glycosylphosphatidylinositol (GPI)-anchored proteins. The gene of this anchor protein is X-linked and as such only haploid in mammalian cells, so that a mutational loss of that protein will generate cells lacking the attached phenotypic marker (Gollapudi et al. 2015). Despite the fact that mutations that lead to a loss of GPI anchors have not been well characterized, it is a gene mutation that constitutes the genetic endpoint addressed so that this test could be a follow-up test to investigate positive Ames test results in vivo. Whereas this assay can be used in various animal species and cell types in vitro and in vivo (Olsen et al. 2017), it is currently being mostly used to quantify CD59-deficient (presumed Pig-a mutant) erythrocytes in the peripheral blood of rats. Thus, it lends itself to being integrated into repeated-dose toxicity studies, since only a minimal amount of peripheral blood needs to be collected for analysis so that even a longitudinal analysis is feasible (Godin-Ethier et al. 2015). The preparation of an OECD Guidance Document for this test is underway, based on recently published detailed experimental descriptions (Gollapudi et al. 2015).

### Protein adducts as human biomarkers

Chemical carcinogens form covalent bonds with nucleophilic sites of physiological macromolecules, e.g. DNA, RNA and proteins. Resulting DNA adducts may lead to mutation events. However, the internal exposure to electrophilic compounds in humans is usually not characterized by DNA adduct levels, mainly due to the limited accessibility of human DNA. Instead, techniques for the assessment of the internal exposure via quantification of protein adducts are well established in the case of haemoglobin (Hb) and serum albumin (SA) as preferred targets (*dosimetry*). Especially thiol groups of cysteine (Cys) residues as well as the nitrogen atoms of histidine (His) and the N-terminal valine (Val) in Hb undergo addition reactions and nucleophilic substitutions leading to the formation of covalent bonds. The resulting adducts may be suitable biomarkers for the internal dose of reactive metabolites. They are not only integral parameters, which reflect the actual intake regardless of the source and the uptake route. They also account for the wide substance-specific interindividual toxicokinetic variations in humans.

Analytical techniques for the adduct quantification were initially established as tools to monitor the occupational exposure to mutagens and carcinogens during work shifts, e.g. to ethylene oxide (Calleman et al. 1978) and aniline (Lewalter and Korallus 1985). More recently, the analytical assessment of protein adducts was introduced in molecular cancer epidemiology. A substance-specific biomarker that complements assessment of dietary exposure may be supportive to uncover causal associations between the uptake of food-borne carcinogens and cancer risk.

The blood proteins Hb and SA are suitable targets for the analysis of the adduct load in humans. Both proteins are available in relatively large quantities (~ 150 mg Hb/ml blood, ~ 30 mg SA/ml blood). The time intervals of internal exposure monitoring to electrophilic compounds are primarily determined by the lifespan of red blood cells for Hb (120 d in humans) and the half-life of SA (*t*_1/2_ = 20 d in humans). Because of the relative longevity of Hb, monitoring of Hb adducts is preferred over that of SA adducts. However, since the number and accessibility of nucleophilic amino acid side chains on the protein surface determine their reactivity, monitoring of SA adducts may be a more sensitive parameter compared to those in Hb (Rappaport et al. 2012). Moreover, SA is biosynthesized in hepatocytes, in which the bioactivation of many carcinogens takes place.

To monitor short-term exposures, e.g. immediately after accidental scenarios or short term nutritional exposure, it may be advisable to quantify mercapturic acids. They are formed from glutathione conjugates of reactive metabolites and are excreted in the urine. Mercapturic acids are detected shortly after the uptake of chemicals and are eliminated usually within 24 h. As a consequence, they are sensitive biomarkers of choice to monitor short-term exposures, which may not cause measurable changes of adduct levels in blood proteins (Mathias and B'Hymer 2014) (see also part B "Selected examples", Sec. “Acrylamide”).

#### Methodological aspects of protein adduct isolation

Among numerous techniques for the cleavage or the isolation of adducts from proteins the most common methods are the Edman degradation of the *N*-terminal Val of Hb, the enzymatic hydrolysis of either Hb or SA (usually with the aim of extracting adducts from single amino acids) and the cleavage of sulfinamides to release aromatic amines from Cys adducts (Törnqvist et al. 2002). The concept of using Hb as a molecular dosimeter for alkylating agents was introduced by Ehrenberg and co-workers (Ehrenberg et al. 1974; Osterman-Golkar et al. 1976). The most important reason for its wide applicability is the reactivity of the N-terminal Val residues. The amino group of the free Val (pKa = 9.74) is expected to be largely protonated in the blood (pH ~ 7.4). In the protein environment of Hb the nitrogen acidity of the Val residues, which are somewhat shielded, is increased in the subunit α (pKa = 7.8) and β subunit (pKa = 6.8), thereby resulting in large fractions of unprotonated primary amine groups (Törnqvist et al. 2002). Due to its convenience, the Edman degradation of modified N-terminal Val residues is the most well-established technique for studying protein adducts in humans (Fennell et al. 2005; Rydberg et al. 2009; Törnqvist et al. 1986).

Alternatively, adducts may be quantified after the proteolytic digestion of blood proteins to mixtures of peptides and single amino acids. In this case, serum proteins such as SA are the preferred molecular targets (Sabbioni and Turesky 2017; Skipper and Tannenbaum 1990; Yang et al. 2014). The applicability of this method may be somewhat restricted due to the limited efficiency of the digestion. Peng and Turesky (2014) tested the proteolytic cleavage with five different mixtures of enzymes to analyse the adduct of the food carcinogen 2-amino-1-methyl-6-phenylimidazo(4,5-*b*)pyridine (PhIP) at Cys^34^ in SA. The results indicated that the complete enzymatic hydrolysis of SA to the level of single amino acids was not achieved, even with mixtures of various proteolytic enzymes (Peng and Turesky 2014). These authors concluded that it is impossible to determine the digestion efficiency, which prohibits referring adduct levels determined by mass spectrometric means to the amount of protein used for the analysis.

A third method was specifically developed for the release of Hb adducts from carcinogenic aromatic amines and nitroarenes. Aromatic amines are exceptionally well studied due to their relevance in occupational health, e.g. aniline and toluidine (Teass et al. 1993), and their presence in tobacco smoke, e.g. 4-aminobiphenyl (Bryant et al. 1987). The compounds are metabolized to arylhydroxylamines, which are oxidized to arylnitroso intermediates. These are prone to form sulfinamide adducts with Cys residues. The cleavage under acidic or alkaline conditions yields the free parent amines, which can be readily quantified by mass spectrometric methods (Skipper and Stillwell 1994).

#### The simultaneous analysis of multiple protein adducts

With the availability of increasingly sensitive GC-MS and LC-MS/MS techniques it became feasible to study the background levels of adducts originating from the continuous intake of low doses of environmental and food carcinogens. However, as yet there are only a few applications of protein adduct analysis in molecular epidemiology. In part, this is due to the perception that it is implausible to correlate low exposure to single carcinogens in the diet with tumour formation. Such an association was seldom shown. An example is the case of aflatoxin B1 and hepatocellular carcinoma (Liu and Wu 2010; Wogan 1992) (see also part B "Selected examples", Sec. “Aflatoxin B1”). It is more probable that the internal exposure to the whole array of exposure relevant compounds, the so-called *exposome*, and the subsequent global genotoxic insult may be responsible for tumour development. The challenge is to assess the internal doses of a wide range of electrophilic compounds, e.g. by simultaneous monitoring of protein adducts. First proof-of-principle studies focused on the adduct arrays of single amino acid hot spots in Hb (Carlsson et al. 2014) or SA (Li et al. 2011a; Osaki et al. 2014), e.g. the N-terminal Val in Hb or Cys^34^ in SA. Li et al. (2011a, b) described a mass spectrometric method for the profiling of adducts at Cys^34^ after isolation and tryptic digest of SA from human blood samples. This allowed to discriminate blood samples of smokers and non-smokers. However, the adducts and their biochemical origin were not characterized (Li et al. 2011a). Carlsson et al. (2014) presented a novel screening strategy for unknown Hb adducts using the modified Edman degradation with Fluorescein-5-isothiocyanate (FITC) as the cleavage reagent for the *N*-terminal Val. The technique allowed detecting seven known and 19 unknown Val adducts in human Hb (Carlsson and Tornqvist 2017; Carlsson et al. 2014).

In summary, the progress in the area of simultaneous monitoring of protein adducts that reflect the internal exposure to a range of reactive compounds is relatively slow. It becomes clear that the monitoring of a large range of electrophilic compounds may require more than one technique of adduct isolation because different nucleophilic sites within a protein react with different classes of electrophiles. One of the methods in the toolbox for the future characterization of the human exposome may be the Edman degradation.

#### Protein adduct analyses in occupational medicine

Human biomonitoring (HBM) is used as a diagnostic tool to estimate the health risk resulting from the long-term exposure to a hazardous compound e.g. at the workplace. The MAK Commission of the German Research Foundation (DFG) derived reference values for the concentrations of Hb adducts for some toxicologically relevant substances. For example, health-related parameters, e.g. so-called Biological Reference Values (BAR values) for workplace substances were determined to evaluate biomonitoring results of adducts from acrylamide (50 pmol/g Hb), acrylonitrile (10 pmol/g Hb), 1,2-epoxypropane (10 pmol/g Hb), 4-aminobiphenyl (after release from Hb, 15 ng/l) and 4,4´-diaminodiphenylmethane (after release from Hb, < 5 ng/l) (DFG 2018). However, the BAR values exclusively reflect the background levels of protein adducts of environmental agents in the not occupationally exposed reference population and thus cannot be used directly to assess the health risk resulting from a particular exposure and increases of adduct levels.

In the case of carcinogenic compounds, there are usually no applicable risk-based exposure limits in occupational medicine. However, in some cases, acceptance and tolerance concentrations that result in an estimated excess tumour risk of 4:10,000 and 4:1000, respectively, after life-time inhalative exposure to a specific compound at the workplace were derived by the Committee on Hazardous Substances (AGS) (AGS 2014). For some of the substances, the MAK Commission also derived Exposure Equivalents for Cancerous Substances (EKA) values. Usually, the EKA values describe the correlation between the concentration of a carcinogen at the workplace air and of the substance or its metabolites in a biological sample. However, there are also EKA values including levels of Hb adducts as measures of the internal exposure of the carcinogenic substances (DFG 2018). Based on the combination of acceptance values or tolerance values and the EKA values risk-related parameters were derived for the acceptance concentration of Hb adducts (equivalent value for an excess risk of 4:10,000) of acrylamide (400 pmol/g) and acrylonitrile (650 pmol/g) and for the tolerance concentration (equivalent value for an excess risk of 4:1000) of Hb adducts of acrylonitrile (6500 pmol/g) and ethylene oxide (3900 pmol/g) (AGS 2014).

#### Protein adducts as exposure markers of dietary and environmental carcinogens

The correlations between intake of dietary and environmental carcinogens and the development of tumours in humans are difficult to study due to various problems. For example, the assessment of exact exposure levels over a long time is difficult and, in addition, there are wide substance-specific interindividual toxicokinetic variations. It is, therefore, reasonable to use biomarkers that reflect the actual intake and the toxicokinetic properties of a mutagenic substance. Early examples come from studies of inhalative exposure to tobacco smoke containing aromatic amines, which form sulfinamide linkages with Cys residues in Hb. High concentrations of Hb adducts derived from 4-aminobiphenyl and other arylamine-sulfinamides were associated with an increased risk of bladder cancer in smokers but also in non-smokers (Skipper et al. 2003; Yu et al. 2002). The progress in the field of protein adduct analysis is demonstrated by recently developed mass spectrometric techniques for the determination of adducts from various mutagenic and carcinogenic substances in food, for example, from aflatoxin B1 (Lys-AFB 1 in serum protein (McCoy et al. 2005)), from the glucosinolate neoglucobrassicin (*N*-(1-MIM)-His in Hb and SA of mice (Barknowitz et al. 2014)), benzo[*a*]pyrene (His-adduct of the reactive ( ±)-anti-benzo[*a*]pyrene-7,8-diol-9,10-epoxide (BPDE) in mouse SA (Westberg et al. 2014)), and from PhIP (the peptide LQQC^34^(PhIP)PFEDHVK in SA (Peng and Turesky 2011) and from the heat-induced contaminants glycidol (Hielscher et al. 2017) and furfuryl alcohol (Sachse et al. 2017) (Val-adducts in Hb). However, there only are a few studies on the molecular epidemiology of dietary compounds reporting associations between protein adduct levels and cancer risk. The Hb adducts of acrylamide and the reactive metabolite glycidamide at the *N*-terminal Val were used as biomarkers of the internal dose in a various nested case–control studies. For example, a weak positive association between levels of the acrylamide adduct *N*-(2-carbamoylethyl)Val from Hb in 269 breast cancer cases was reported from the “Danish Diet, Cancer and Health Study” with an incidence ratio of 2.7 fold per tenfold increase of *N*-(2-carbamoylethyl)Val (95% CI 1.1–6.6) (Olesen et al. 2008). In 170 prostate cancer patients from the “Cancer of the Prostate in Sweden Study” there was no correlation detected between Hb adduct levels of acrylamide and glycidamide and the tumour incidence (Wilson et al. 2009). Moreover, there was no evidence for an association between the increased risk of epithelial ovarian cancer in 263 cases and elevated levels of Hb adducts of acrylamide and glycidamide (Xie et al. 2013). It is important to note, that epidemiological studies have failed to demonstrate a correlation between dietary acrylamide intake and an increased risk of tumour incidence (Lipworth et al. 2012). The demonstration of a correlation between acrylamide intake and the formation of acrylamide and glycidamide adducts by future duplicate studies may support the detection of associations in the field of cancer epidemiology. Another interesting example for the application of a protein adduct as a biomarker for the internal exposure to an endogenous carcinogen is the quantification of Cys adducts of 17β-estradiol-2,3-quinone and 17β-estradiol-3,4-quinone in Hb of blood samples from breast cancer patients in Taiwan. The analyses showed that the concentrations of estrogen quinone-derived adducts were on average six times higher in the group of patients than in the control group, thereby supporting the relationship between elevated estrogen plasma levels and breast cancer (Lin et al. 2014).

#### Future risk assessment using protein adducts of food and environmental carcinogens

Protein adducts formed by reactive xenobiotic carcinogens, their respective metabolites and some electrophiles of endogenous origin may not directly reflect the genetic damage. Correlations between adduct levels in blood proteins and risks of tumour development in particular tissues should be considered with caution. One of the factors which might influence the adduct levels of plasma proteins is their biological stability, e.g. resistance to proteolysis after modification. Proteolytic activities are influenced by individual factors as genetics, lifestyle, age etc. The above-mentioned definition of acceptance concentrations and tolerance concentrations by the AGS are exceptions, in which protein adduct levels of occupationally relevant substances were associated with specific excess risks of tumour formation in the working population (AGS 2014).

An interesting case for a well-established correlation of exposure → protein adducts → cancer incidence was described for 4-aminobiphenyl. The levels of the sulfinamide adducts of the aromatic amine in Hb were shown to increase with the number of cigarettes smoked per day, and the protein adduct levels were correlated with the DNA adduct concentration of *C*8-(4-aminobiphenyl-*N*^4^-yl)-2′-deoxyguanosine (dG-*C*8-4ABP) in exfoliated urothelial cells. Furthermore, the increase of the protein adduct concentration was correlated with increased bladder cancer risk (Turesky and Le Marchand 2011). However, such correlations between external dose, biomarkers of internal exposure and tumorigenic effect are still an exception.

Risk assessment may also be supported by detailed information on the correlation of external exposure and protein adducts. This may allow determining the external exposure from the measurement of protein adduct levels on an individual basis. Recently, Abraham et al. described the dose-specific increase of the adduct *N*-(2,3-dihydroxypropyl)Val in Hb after intentional exposure to glycidyl esters in a human study (*n* = 11) using a commercially available fat. From this increase, a mathematical model was used to calculate the external exposure in the group of participants in the 4 months prior to the start of the controlled exposure study. Of course, this approach seems only possible if the interindividual variations of bioactivation and detoxification of the substance in question are relatively small (Abraham et al. 2019).

One obstacle in the risk assessment of carcinogenic substances is the extrapolation of the association between dose and tumour incidence from 2-year bioassays to the human situation. This is not easy due to various reasons, for example, because the metabolic capacities for activation and detoxification in animal models and humans are different. To support risk assessment, key enzymatic parameters may be determined in vitro at the level of individual enzymes or tissue samples (Sachse et al. 2016). Alternatively, protein adducts as biomarkers for the characterization of the species-dependent metabolic activation may allow to compare the internal exposure resulting from a defined external dose between animals and humans. With this approach, the internal exposure to 1,2:3,4-diepoxybutane, a reactive metabolite of the industrial building block 1,3-butadiene, was compared in mice, rats and humans. The metabolite forms the *N*,*N*-(2,3-dihydroxy-1,4-butadiyl)Val adduct at the N-terminus of Hb. A tryptic digest yields the peptide containing the modified Val (*pyr*-Val), which is readily quantified by LC–MS/MS (Boysen et al. 2004). After inhalative exposure to five different concentrations between 0.1 ppm and 625 ppm 1,3-butadiene, mice had 10- to 60-fold higher levels of *pyr*-Val if compared to rats (Georgieva et al. 2010). These findings supported the hypothesis that the relative tumour susceptibility of mice may partially be attributed to enhanced 1,3-butadiene bioactivation (NTP 2011). Georgieva et al. (2010) analysed *pyr*-Val levels in a group of over 300 industrially workers exposed to 1,3-butadiene. In animal models, the exposure to 0.1 to 1 ppm, which corresponds to the ambient 1,3-butadiene concentrations detected at workplaces, led to approximately tenfold and 100-fold higher levels of *pyr*-Val in rats and mice, respectively, compared to humans (Georgieva et al. 2010). These findings indicate that rodent models are more sensitive to 1,3-butadiene bioactivation when compared to humans. The future risk assessment of dietary carcinogens may be improved by replacing the external dose parameters by protein adduct monitoring in humans in, e.g., the margin of exposure calculation.

In summary, blood protein adducts are ideal biomarkers for the characterization of the internal exposure to electrophilic compounds due to the relatively long lifetime of Hb and SA, their accessibility and their abundance. In the area of occupational health, protein adducts are routinely analysed to monitor the internal exposure to reactive metabolites of particular substances at the workplace. The biomonitoring results are usually evaluated using reference parameters, e.g. the BAR values of the German MAK Commission. Currently, efforts are directed towards the further development of the analytical techniques as well as the application of protein adduct analyses in other scientific areas. In molecular epidemiology, for example, protein adducts may complement or replace the parameter of external exposure to dietary and environmental carcinogens to support the detection of hitherto unknown associations between exposure and tumour development. Very few studies, mainly on dietary acrylamide exposure, have been published (Olesen et al. 2008; Wilson et al. 2009; Xie et al. 2013). They have limited significance due to the small numbers of participants and analyses of single blood drawings.

Protein adduct quantification may also support current routine procedures in risk assessment. The adduct concentration resulting from a defined dose of a particular substance in humans may be compared to the same parameter in animal models, for which also carcinogenicity data may be available. This allows to compare the metabolic bioactivation and detoxification rates in humans and animals to support risk assessment. A promising technical advancement is the implementation of mass spectrometric techniques for the simultaneous quantification of multiple protein adducts. Their incorporation into human biomonitoring may greatly increase our knowledge on the internal human exposure to common food and environmental carcinogens.

### Toxicogenomics for hazard identification and risk assessment

Elucidating the nature of the dose–response relationship, particularly in the low dose range, requires a detailed understanding of the biological response to xenobiotic exposure. Therefore, it seems promising to use a toxicogenomic approach which brings together the knowledge gained from toxicology, genomics and bioinformatics. The diverse technologies that are subsumed under the term “toxicogenomics” have been described in detail by Ellinger-Ziegelbauer and Ahr (2014). Confusingly, the term “toxicogenomics” is used in two ways, on the one hand it is used as a general term for omics techniques applied to toxicological studies, and on the other hand it refers to the analysis of gene expression profiles (transcriptomics). Further toxicogenomic techniques monitor, for instance, functionally relevant changes in the genome (epigenomics), global protein and/or post-translational modifications (proteomics) or metabolites (metabolomics). To date, transcriptomics is the most frequently used omics technique with the most advanced quality standards, so that only this approach is described below (Kauffmann et al. 2017; Sauer et al. 2017).

However, the plethora of data collected by omics techniques is challenging regarding the identification of patterns of genes, proteins or metabolites that report specific exposures and their biological consequences. A reason for this lies in the fact that alterations in the expression or regulation of biomolecules do not necessarily indicate an adverse effect. To identify the biological event related to these changes, the toxicogenomic data have to be linked to toxicological or apical endpoints, the so-called “phenotypic anchoring” (Buesen et al. 2017; Paules 2003). Moreover, the application of bioinformatic tools such as principal component analysis, clustering, statistical comparison of classes, class prediction or the mechanistic analysis is an effective approach to extract patterns or signatures from toxicogenomic datasets that are differentially expressed (Afshari et al. 2011). Meanwhile, a framework has been developed to incorporate bioinformatics procedures in the whole processes of data generating and storage, data processing and data interpretation (Gant et al. 2017). A common type of data interpretation entails comparing sets of genes in terms of their functional annotations, for instance to identify functions that are enriched or depleted in particular subsets of genes, using Gene Ontology (GO) (Gaudet and Dessimoz 2017). Since this tool is based on existing knowledge, however, some bias may be introduced in the course of data interpretation and may compromise the detection of an essential but not yet intensively investigated process. Nevertheless, the ongoing work particularly in the field of mathematical modelling and linking pathway perturbations measured at the omics level to apical endpoints will contribute to a process of constant improvement.

#### Hazard identification

Until now, toxicogenomics has traditionally been applied in regulatory toxicology for hazard identification with signatures used to distinguish different chemical classes, in particular genotoxic and non-genotoxic carcinogens. This concept was first introduced by Nuwaysir et al. (1999). The authors intended to derive characteristic gene signatures elicited by model compounds to enable the classification of other compounds with unknown toxicity. In addition, they emphasized the potential of this technique to elucidate molecular mechanisms underlying toxicological effects. Subsequently, numerous groups have demonstrated the utility of toxicogenomics in classifying and predicting the carcinogenic potential of compounds (Eichner et al. 2014; Ellinger-Ziegelbauer et al. 2004; Jackson et al. 2017; Rieswijk et al. 2015; Schaap et al. 2015; Suenaga et al. 2013; Williams et al. 2015; Yauk et al. 2016). Using various mathematic tools, they described signature gene sets which have the potential to predict the carcinogenic potential of chemical compounds even in in vitro systems, which is an important point in the view of the growing awareness of avoidable animal studies (Li et al. 2017). According to these studies, genotoxic carcinogens have a well-described MOA, which essentially leads to the activation of p53 tumour suppressor gene products in response to DNA damage. This, in turn, will initiate a cascade of pathways such as DNA damage response, DNA repair response, apoptosis or cell cycle arrest (Ellinger-Ziegelbauer et al. 2004). In contrast, non-genotoxic carcinogens act through several distinct pathways including increased cell proliferation, decreased apoptosis, energy depletion or production of reactive oxygen species (Deferme et al. 2015). In addition, they have been shown to act as tumour promoters, e.g. by acting as peroxisome proliferators, endocrine disruptors, receptor mediators or immunosuppressants (Rieswijk et al. 2015). However, this dichotomous way of classification based on the genotoxic potential of a compound is primarily a qualitative hazard directed method reflecting the restriction of the predictive potential of toxicogenomic techniques.

#### Risk assessment

Hence, there is a growing demand for a shift/transition from qualitative hazard identification to quantitative dose–response analysis, which is crucial for the application of toxicogenomic data in the field of risk assessment. Only a quantitative approach enables the (mathematical) determination of the low dose–response relationship and, consequently, of the point of departure (POD) both of which are essential for risk assessment and regulatory decision-making (Johnson et al. 2015; Li et al. 2017). Until now, few groups have attempted to evaluate the utility of toxicogenomics in risk assessment and to compare this approach with the traditional one (Farmahin et al. 2017; McMullen et al. 2016; NTP 2018).

In basic work in this field, Thomas and co-workers exposed mice for 13 weeks to five chemical carcinogens (Thomas et al. 2011, 2012). In the first step, they calculated the BMDs for each gene to determine at which point of the dose–response curve the majority of pathways became transcriptionally active. In the next step, a gene enrichment analysis was performed to determine which functional pathways, resp. processes (also termed GO category) were activated. Finally, the average BMD and BMDL values were calculated for each GO category. The pathway or GO category with the lowest median transcriptional BMD/BMDL—regardless of the biological function—was compared with the corresponding values for the apical endpoints (such as liver weight or histological changes in target tissues, resp. incidence of combined adenomas and carcinomas). The authors concluded that the transcriptional values showed a good correlation with the values derived from the traditional endpoints leading to the suggestion that the lowest transcriptional BMD/BMDL value should be used as a POD.

The qualitative response to benzo[a]pyrene (B[a]P) was investigated in a comprehensive manner by integrated literature-based traditional data for apical endpoints with toxicogenomic data derived from in vitro human cell cultures as well as from tissues, organ systems, or entire organisms (Moffat et al. 2015). The basis for the development of the MOA was the enrichment analysis of the genomic data to detect activated pathways as well as the doses and the time points at which they were affected. In the next step, based on these key events “mutations” were selected as the decisive/irreversible step towards carcinogenesis and the preceding key event “DNA adducts and DNA damage” as the POD to adequately protect against the carcinogenic outcome. The calculated BMDL values for the traditional and transcriptional approaches were in the same range (liver 1.2 vs. 1.0 mg/kg bw/day; lung 0.8 vs. 3.7 mg/kg bw/day; forestomach 0.5 vs. 7.4 mg/kg bw/day). The authors argued that the differences in the PODs concerning the forestomach could be due to the delay of three days between the last exposure and transcriptional profiling. Applying the approach using GO-enrichment analysis described by Thomas et al. (2011, 2012), they obtained BMDL_10_ values of 0.2 mg/kg bw/day for liver; 2.1 mg/kg bw/day for the lung and 4.5 mg/kg bw/day for the forestomach. The authors concluded that the approach of Thomas et al. may be useful in preliminary studies with unknown or unclear MOA.

In subsequent studies, the group of Yauk applied this approach to derive PODs for furan and multi-walled carbon nanotubes (Dong et al. 2015; Labib et al. 2016). In both studies, transcriptional BMDs were comparable to traditional BMDs. Other groups also use this approach to derive PODs for various chemical compounds, such as naphthalene, nickel subsulfide or cholestatic drugs (Clewell et al. 2014; Efremenko et al. 2014; Kawamoto et al. 2017). An important and critical point in the application of toxicogenomics in this field is the determination of the best way to select predictive groups of genes. Farmahin et al. evaluated 11 approaches and compared the transcriptional BMD values with BMDs derived from apical endpoint changes. Four approaches led to BMDs showing a good concordance with apical BMD values (Farmahin et al. 2017).

A conservative approach is taken by groups that focus on the “No-Transcriptional-Effect-Level” (NOTEL), threshold at which no effect on the transcriptome is observed (Pisani et al. 2015; Quercioli et al. 2018; Zarbl et al. 2010). As to be expected, the derived NOTELs were considerably lower than the “No-Observed-Adverse-Effect-Levels” (NOAELs). One possible application could be the quick estimate of a benchmark dose of chemicals and mixtures, especially within the framework of large toxicology programs.

A slightly different approach was chosen by Ji and co-workers (Ji et al. 2016) who determined what they called a “No-Observed-Genotoxic-Effect-Level” (NOGEL) based on blood reticulocyte micronuclei number of rats exposed to methyl methanesulfonate (MMS) and methylnitrosourea (MNU). Whole-genome transcript analysis of the liver demonstrated no statistically significant gene alterations below the NOGEL.

Taken together, the studies show how toxicogenomic approaches may become a useful complement to hazard identification and risk assessment. These techniques, if properly used, provide information that can improve our mechanistic understanding of dose–response relationships and, in consequence, of biological thresholds. One advantage of these approaches is the considerable amount of data which can be obtained in a time- and cost-optimized way when compared to 2-year rodent cancer bioassays, thus minimizing animal use. A considerable number of studies have demonstrated the utility of toxicogenomics to rapidly identify genotoxic and non-genotoxic carcinogens, even based on in vitro studies. In addition, it becomes apparent that patterns or signatures of genes can be used to develop MOAs and to identify key events, which in turn allow the calculation of PODs.

Nevertheless, several points need to be critically addressed. First, genes of unknown function are not included in pathway and enrichment analyses. This possible bias must be taken into account, as previously mentioned, since it can prevent the recognition of important pathways. In cases where metabolic activation of premutagens is required the choice of the metabolizing system is pivotal.

Second, POD calculation requires a clear definition regarding the BMDL to be selected. The selection of the lowest transcriptional BMDL is highly conservative and does not necessarily reflect the dose–response relation and/or the apical adverse effect (Farmahin et al. 2017). This may be improved by distinguishing adaptive from adverse effects and by ascertaining pathways, dose and exposure time that indicate or cause the transition from one state to another.

Third, to utilize toxicogenomic data in risk assessment, effects have to be quantified, particularly at low doses. Since the results of microarray analysis are only semi-quantitative, quantitative high throughput RT-qPCR analysis represents a serious alternative technique. A step in this direction was undertaken by Fischer et al. who analysed the expression of 95 genes related to distinct pathways relevant for genomic stability by high-throughput RT-qPCR (Fischer et al. 2016).

In summary, toxicogenomic techniques have the potential to complement the existing approaches for hazard identification and risk assessment. When reasonably applied to toxicological issues and using appropriate experimental designs and quality standards, the information obtained by omics technologies can provide valuable insights into a variety of aspects of the toxic response. However, the low dose range is particularly important, but still largely unexplored by toxicogenomic approaches.

### Background DNA lesions in rodent and human tissues/body fluids

The ability to exactly identify and measure DNA lesions in tissues and body fluids has remarkably increased in recent years. Reliable dosimetry of DNA damage associated with exposure to minute traces of genotoxic contaminants in food and other consumer media can be achieved with present day advanced instrumental analysis. However, it is important to take into account that cells are continuously exposed to genotoxic agents leaking from endogenous metabolic processes in the frame of normal physiological nutrient turnover. This encompasses not only reactive oxygen radicals (ROS) but also many other endogenous substrates, such as ethylene and its epoxide, formaldehyde, acetaldehyde, lipid peroxidation products, acrolein equivalents and others.

#### DNA adducts arising from endogenous processes

##### Lipid peroxidation products

For example, aldehydes formed from lipid peroxidation are able to form DNA adducts such as etheno, propano and malondialdehyde adducts. The etheno adducts 1,*N*^6^-etheno-deoxyadenosine (εdA) and 3,*N*^4^-ethenodeoxycytidine (εdC), which are formed from reactions of DNA bases with 2,3-epoxyaldehydes of 4-hydroxy-2-nonenal or crotonaldehyde, were the most abundant adducts detected in human lung samples (De Bont and van Larebeke 2004; Swenberg et al. 2011). Markedly different half-lives have been reported for etheno adducts. The *N*^2^,3-ethenoguanine (εG) adduct has by far the longest half-life (150 days in rats). In contrast, εdA is rapidly repaired with a half-life of ~ 24 h (Swenberg et al. 2011). Another product of lipid peroxidation, malondialdehyde, mainly forms deoxyguanosine (dG) adducts such as pyrimido[1,2-a]-purin-10(3H)-one (M1G), to a lesser extent also deoxyadenosine (dA) and deoxycytidine (dC) adducts (De Bont and van Larebeke 2004).

##### Endogenous alkylating compounds

In addition to ROS and lipid peroxidation products, several other reactive molecules with the propensity to interact with DNA as electrophiles are produced in the organism. The methyl group donor *S*-adenosylmethionine (SAM) is essential for physiological enzymatic methylation but may also contribute to endogenous DNA base methylation, including 7-methylguanine (7-MG), 3-methyldA or *O*^6^-methyldG (Nakamura et al. 2014). 7-MG is the most frequent alkylation product but does not alter the coding specificity of the DNA base whereas *O*^6^-methyldG is highly promutagenic and can result in mismatches during DNA replication (Nakamura et al. 2014). Amongst others, the generation of ethylene from methionine oxidation, lipid peroxidation, and bacterial metabolism can give rise to ethylene oxide, another endogenous electrophile that preferentially reacts with the N7-position of guanine, forming the DNA lesion N7-(2-hydroxyethyl)guanine (7-HEG) (Swenberg et al. 2011). Although 7-MG and 7-HEG are not considered to be promutagenic by themselves, they may lead to abasic sites through depurination which eventually could result in a mutation if unrepaired (Swenberg et al. 2011). Abasic sites are among the most frequent endogenous lesions found in DNA (De Bont and van Larebeke 2004).

Formaldehyde is a metabolic intermediate generated in all living cells from methanol, continuously generated during food digestion, but also from other precursors like serine, glycine, methionine, choline and/or by oxidative demethylation of a wide variety of substrates. Formaldehyde can induce DNA adducts including *N*^2^-hydroxymethyl-dG, *N*^6^-hydroxymethyl-dA, as well as *N*^4^-hydroxymethyl-dC and, in turn, DNA protein crosslinks. Those DNA adducts are considered to be promutagenic, as the amino groups participating in Watson–Crick base pairing are involved and DNA protein crosslinks are formed which give rise to double-strand breaks (Lai et al. 2016; Swenberg et al. 2011).

#### Steady-state levels of endogenous DNA lesions

A compilation of steady-state levels of selected endogenous DNA lesions reported in the literature (Nakamura et al. 2014; Swenberg et al. 2011) is given in Table 1. Concerning 8-oxo-dG and abasic sites, the levels observed in earlier studies appear to be highly overestimated due to technical artefacts. A multicentre study comparing different methods to quantify 8-oxo-dG came to the conclusion that the actual levels of this lesion in cultured mammalian cells are clearly below 100 lesions per 10^8^ nucleotides (Collins et al. 2004; Gedik et al. 2005). In the case of abasic sites, the use of repair enzymes as probes revealed levels in cultured mammalian cells that are much lower than those of 8-oxo-dG and close to the detection limit. Data obtained from formamidopyrimidine DNA glycosylase (Fpg) treatment suggest approx. 5 lesions per 10^8^ nucleotides (Andersen et al. 2005; Sossou et al. 2005). The so-called "aldehyde reactive probes", which have frequently been employed in the quantification of abasic sites, have indicated, for example, in HeLa cells levels of 200 lesions per 10^8^ nucleotides. Again, this method may suffer from considerable background problems and thus result in overestimation of levels of abasic sites (Wei et al. 2015). In view of these inconsistencies, Table 1 exclusively lists well-defined adducts.

**Table 1:** Compilation of endogenous levels of DNA lesions estimated for human and animal cells (modified from Nakamura et al. (2014); Swenberg et al. (2011))

Endogenous DNA lesions^1^	Number per cell	Number per 10^8^ nucleotides^2^
N7-(2-Hydroxyethyl)guanine (7-HEG)	3000	25
8-Oxo-dG	2400	20
N7-(2-Oxoethyl)guanine (7-OEG)	3000	25
Formaldehyde adducts	1000–4000	8–33
Acetaldehyde adducts	1000–5000	8–42
7-Methylguanine (7-MG)	2300	19
Acrolein-deoxyguanosine	120	1
Malondialdehyde-deoxyguanosine (M1G)	60	0.5
*N*^2^,3-Ethenoguanine (εG)	36	0.3
1,*N*^2^-Ethenodeoxyguanosine (1,*N*^2^-εdG)	30	0.25
1,*N*^6^-Ethenodeoxyadenosine (1,*N*^6^-εdA)	12	0.1
*O*^6^-Methyldeoxyguanosine	2	0.016
Total	13,000 + (13,000–20,000)	107 + (107–167)

^1^Nomenclature as indicated by the authors

^2^Assuming 6 billion bp/ 12 billion nucleotides per diploid cell (https://www.ncbi.nlm.nih.gov/genome/51)

#### Selected DNA lesions in rodents and humans

Selected DNA lesions measured in human cells and body fluids are listed together with the putative causal agents in Table 2 and corresponding data in rodent cells and body fluids are listed in Table 3. Although not being exhaustive, the tables allow for an estimate of the overall endogenous adduct levels. However, levels of a specific adduct can easily vary by more than one order of magnitude, even within the same species and organ (Paini et al. 2011). In addition, depending on the type of adduct, adduct levels tend to be somewhat higher in humans than in laboratory rats kept under controlled housing conditions. This has been ascribed to lifestyle factors such as unknown dietary and environmental sources of exposure to alkylating agents, smoking or oxidative/metabolic stress (Paini et al. 2011). Thus, especially lesions derived from lipid peroxidation products such as 7-HEG, M1G and other cyclic adducts have been found to be about one order of magnitude higher in humans than in animals (see Tables2 and 3). For example, 7-HEG adduct levels (supposedly from ethylene oxide) have been reported to be 48–300 adducts/10^8^ nucleotides in humans (Farmer and Shuker 1999; Wu et al. 1999a) versus 1–9 adducts/10^8^ nucleotides in rats (Marsden et al. 2009, 2007; Wu et al. 1999a; Zhao et al. 1999). Likewise, 14–110 M1G adducts/10^8^ nucleotides were reported in human liver (De Bont and van Larebeke 2004; Farmer and Shuker 1999) versus 0.8–4.2 adducts/10^8^ nucleotides in rat tissues including liver (Jeong et al. 2005). This also applies to some extent to other cyclic base adducts, especially etheno-dA and etheno-dC adducts, which have been found in humans at levels of up to 36–80 adducts/10^8^ nucleotides (Chen et al. 2010a; Monien et al. 2015), in rats at a level of 0.7–1.4 adducts/10^8^ nucleotides (Morinello et al. 2002; Swenberg et al. 2011).

**Table 2:** Selected DNA lesions in human tissues/body fluids determined by high precision instrumental analysis

Lesion^1^	Human tissue/body fluid	Presumed agent/ exposed to	Level (adducts/10^8^ nucleotide^2^)	References
*N*^2^-Ethylidene-dG detected as *N*^2^-Ethyl-dG^3^	Blood cells	Ethanol/acetaldehyde:		Balbo et al. (2008)
		Non drinkers	59	
		Drinkers	116	
	Lung tissue	Smokers & non-smokers	13	Singh et al. (2009b)
	Granulocytes & lymphocytes	Ethanol (0.05–0.07% blood ethanol)	150 (background level) up to about fivefold after ethanol consumption	Balbo et al. (2012a); Balbo et al. (2012b)
	Liver	Acetaldehyde	12	Wang et al. (2006)
7-Ethyl-G			0.8	Chen et al. (2007)
7-(2´-Carboxyethyl)-G	Liver	Acrylic acid/acrolein	7.5	Cheng et al. (2010)
*1,N*^*2*^*-*Propano-dG	Liver, lung	Crotonaldehyde/ acetaldehyde	0.3–0.4	Zhang et al. (2006)
Etheno-base adducts (1,*N*^6^-Etheno-dA; 3,*N*^4^-Etheno-dC; 1,*N*^2^-Etheno-dG)	Leukocytes	Lipid peroxidation products	~ 36 (averaged mean values)	Chen et al. (2010a)
3,*N*^4^-Etheno-dC	Lung	Lipid peroxidation products	~ 80	Monien et al. (2015)
1,*N*^6^-Etheno-dA	Lung	Lipid peroxidation products	~ 48	Monien et al. (2015)
*N*^2^-(trans-Methylisoeugenol-3‘-yl)-2‘-dG	Lung	Methyleugenol	~ 11	Monien et al. (2015)
*N*^*2*^*,3-*Etheno-G	Liver		2	Farmer and Shuker (1999)
N7-(2-Hydroxyethyl)-G (7-HEG)	Liver Lymphocytes	Ethene (from methionine oxidation, lipid peroxidation and bacterial metabolism)	58 48	Wu et al. (1999a)
			300	Farmer and Shuker (1999)
7-Alkyl-G adducts (combined 7-MG and 7-HEG)	Lung (*n* = 2) WBC (*n* = 8) (non-smokers)		36–44 29	Zhao et al. (1999)
M1G (Pyrimido[1,2-a]-purin-10(3H)-one)	Liver Leucocytes	Lipid peroxidation products	50–110 6	Farmer and Shuker (1999)
	Liver Lung WBC		14–90 10 26	De Bont and van Larebeke (2004)
*O*^6^ Methyl-G	Liver	Methylating agents	2–13	De Bont and van Larebeke (2004)
*N*^6^-Hydroxymethyl-dA	Leukocytes	Formaldehyde Non-smokers	0.47	Wang et al. (2009)
8-Oxo-dG	Lymphocytes	Oxidative damage, ROS	13–200	Epe (2002)
			< 100	Collins et al. (2004); Gedik et al. (2005)
Abasic sites	Liver	Alkylating agents, oxidative damage	800–900	De Bont and van Larebeke (2004)
	Liver, colon, brain, lung, kidney		370–1130	Barbin et al. (2003)
			~ 5	Andersen et al. (2005); Sossou et al. (2005)

*WBC* white blood cells

^1^Nomenclature as indicated by the authors

^2^In part corrected from the number of adducts per parent base using a content of 22% G or C and 28% of A or T in mammalian DNA (Paini et al. 2011)

^3^After reduction with NaBH_3_CN

**Table 3:** Selected DNA lesions in rodent tissues/body fluids and the respective causative genotoxic agent

Lesion^1^	Rodent tissue/body fluid	Presumed agent/ exposed to	Level (adducts/10^8^ nucleotides^2^)	References
N7-(2-Hydroxyethyl)-G (7-HEG)	Liver (rats)	Lipid peroxidation products	1.3–4.4	Marsden et al. (2009); Marsden et al. (2007); Swenberg et al. (1995)
	Lymphocytes, liver, kidney (rats)	Lipid peroxidation products	6–9	Zhao et al. (1999)
	Liver, spleen, brain, lung (rats, mice)	Lipid peroxidation products	4.4–6.6	Wu et al. (1999a)
M1G (Pyrimido[1,2-a]-purin-10(3H)-one)	Liver, brain, kidney, lung, heart (rats)	Lipid peroxidation products	0.8–4.2	Jeong et al. (2005)
Etheno-base adducts (*N*^2^,3-Etheno-G)	Liver cells (rats):	Lipid peroxidation products		Morinello et al. (2002) Swenberg et al. (2011)
	Hepatocytes		0.7–1.2	
	Nonparenchymal cells		2	
1,*N*^6^-Etheno-dA	Liver (rats)	Lipid peroxidation	1.4	Swenberg et al. (2011)
7-Methyl-G (7-MG)	Lymphocytes, liver, kidney (rats)	Methyl group donors	21–27	Zhao et al. (1999)
*N*^2^-hydroxymethyl-dG	Nasal DNA samples (rats)	Formaldehyde	10 2–15	Swenberg et al. (2011) Lu et al. (2010)
*N*^6^-hydroxymethyl-dA	Nasal DNA samples (rats)	Formaldehyde	2.8–8.4	Cheng et al. (2008); Lu et al. (2010)
8-Oxo-dG	Liver (rats)	Oxidation	170–250^3^	Singh et al. (2009c)
8-Oxo-dA	Liver (rats)	Oxidation	22–26^3^	Singh et al. (2009c)
7-(2-Oxoethyl)-G (OEG)	Liver (rats)		44	Swenberg et al. (2011)
Abasic sites	Brain (rats)		500 up to < 1000	Lan et al. (2003)

^1^Nomenclature as indicated by the authors

^2^In part corrected from the number of adducts per parent base using a content of 22%G or C and 28% of A or T in mammalian DNA (Paini et al. 2011)

^3^Using a chaotropic DNA extraction procedure

Further examples of individual lesions in humans include 7-ethyl-G (0.8 adducts/10^8^ nucleotides) (Chen et al. 2007), and again cyclic adducts, such as 1,*N*^*2*^-propano-dG (0.3–0.4 adducts/10^8^ nucleotides) (Zhang et al. 2006). Higher “background” DNA adduct levels have been reported for 7-(2´-carboxyethyl)G (7.5 adducts/10^8^ nucleotides) (Cheng et al. 2010) and *N*^2^-ethylidene-dG detected after reduction as *N*^2^-ethyl-dG (12.0 adducts/10^8^ nucleotides) (Wang et al. 2006).

Background levels of formaldehyde DNA adducts (*N*^2^-hydroxymethyl-dG and *N*^6^-hydroxymethyl-dA) have been detected in nasal DNA samples of rats at a level of 10.3 adducts/10^8^ nucleotides (Cheng et al. 2008; Lu et al. 2010; Swenberg et al. 2011). The formation of formaldehyde-DNA adducts in humans has also been demonstrated. The endogenous *N*^6^-hydroxymethyl-dA level was determined to be ~ 0.47 adducts/10^8^ nucleotides in human leukocytes from non-smokers (Wang et al. 2009). The overall endogenous background level of DNA lesions induced by alkylating low molecular weight electrophiles has been reported for human and rat tissues to range within about 10–100 adducts/10^8^ nucleotides (Farmer 2008; Paini et al. 2011; Swenberg et al. 2008).

In rodents and humans, the oxidative damage usually is reported to exceed other endogenous DNA lesions such as those generated by alkylation or lipid oxidation (De Bont and van Larebeke 2004; Gupta and Lutz 1999; Paini et al. 2011; Povey 2000). However, as indicated above for cultured cells, the reported basal levels for 8-oxo-dG and abasic sites (see Tables 2 and 3) may be overestimated. The quantification of Fpg-sensitive sites, which include both 8-oxo-dG and abasic sites in the liver and various other tissues of untreated wild-type mice resulted in levels similar to those observed in the cultured cells, i.e. the sum of 8-oxo-dG and abasic sites was found at less than 50 lesions per 10^8^ nucleotides (Osterod et al. 2001). These basal levels are supposed to reflect the equilibrium between the continuous generation and repair of the lesions, which both depend on the cell and tissue type. In proliferating primary human fibroblasts as well as melanoma cells, a half-life of approx. 4 h was reported for Fpg-sensitive modifications (Eiberger et al. 2008). In primary human lymphocytes, however, the repair is very slow but accelerates after stimulation of cell proliferation due to an induction of the expression of OGG1, the major repair glycosylase for 8-oxo-dG. In accordance with the expectation, this is associated with a decrease in the basal levels of Fpg-sensitive modifications (von der Lippen et al. 2015).

With the exception of methyleugenol, ingested through certain foods, herbs and spices, all agents listed in Table 2 may arise from physiological nutrient/energy metabolism. They may as well result from exogenous exposure.

#### Development of a database on background DNA lesions

Clearly, the database on background DNA damage needs to be enlarged and substantiated. This requires to explore its individual/population associated variance and the potential influence of health and age, ethnicity, gender and living conditions. More data are also required to better characterize the correlation between DNA damage and mutation induction. The intrinsic mutagenic potency of DNA modifications is vastly different and ranges over several orders of magnitude (Nestmann et al. 1996). For example, the mutagenic potential of N7-alkyl-dG-adducts is generally considered to be low or even absent. However, an exceptionally mutagenic N7-dG adduct is generated, by the mycotoxin aflatoxin B1 (AFB1). AFB1 is a potent mutagen and carcinogen, primarily reacting at the N7 position of deoxyguanosines in DNA to form, as secondary lesions, abasic sites and highly persistent AFB1-formamidopyrimidines (see also part B "Selected example" Sec. “Aflatoxin B1”) (Smela et al. 2001). To correlate DNA damage and mutation induction, extended case-by-case studies for individual genotoxic agents are required. This may eventually offer the perspective of applying a read-across approach for closely related agents and/or DNA lesions. Thus, the development of a comprehensive database on DNA background damage in humans is considered of great value to inform future risk assessment. This so-called “bottom-up” approach, contrasts with the classical top-down extrapolation from cancer studies in experimental animals (Starr and Swenberg 2013, 2016). A refined example of this “bottom-up” approach has recently been published (Starr and Swenberg 2016). It utilizes background cancer risk and the background (endogenous) level of cancer-related biomarkers in target tissues (e.g. formaldehyde DNA-adducts such as *N*^2^-hydroxymethyl-dG detected in nasal tissue and bone marrow of monkeys) to obtain a slope factor estimate. This estimate is utilized to define the added risk associated with incremental exogenous exposure to such genotoxic agents that invariably are also generated in the body as a result of normal physiologic processes (Starr and Swenberg 2016). Of note, the “bottom-up” estimates of formaldehyde induced nasopharyngeal cancer and leukaemia were markedly smaller than those obtained by the US Environmental Protection Agency (US EPA) with a conventional “top-down” approach on mortality data from a US worker cohort. However, the authors also state that this “bottom-up” approach would likely not apply at exogenous exposures sufficiently high to induce nonlinear processes that amplify the carcinogenic response, such as saturation of metabolic pathways, cytotoxicity and tissue damage, and accelerated regenerative cell proliferation (Starr and Swenberg 2016).

The discrimination between endogenously generated DNA lesions and those induced by exogenous (e.g. nutritional or occupational) exposure requires novel approaches. A good choice appears to be nutritional intervention studies with volunteers (see part C "Conclusions and Perspectives"). Such studies should allow for adequate wash-out periods without any exposure to the agent under investigation, prior to a tightly controlled intervention. It is mandatory for such an approach to ascertain that the endogenous background DNA damage can be measured without interference from exogenous exposure. The subsequent nutritional intervention, e.g. with food containing known (predetermined) levels of a nutritional genotoxic agent present at levels of normal consumer exposure, should provide a metric to discriminate endogenous from exogenous DNA damage (Goempel et al. 2017; Ruenz et al. 2016). Where feasible, this can also be achieved using isotope-labelled agents (Swenberg et al. 2011).

The following part B "Selected examples" of this document discusses specific examples of genotoxic agents humans are exposed to as a result of their living conditions. Living conditions include working place associated exposures as well as those contributed from the environment and from consumption habits. Where applicable, endogenous exposure to such agents is additionally taken into consideration, tracing back to physiological (endogenous) energy metabolism. Accordingly, this group encompasses compounds humans are exposed to exogenously and endogenously, such as formaldehyde, acetaldehyde and the corresponding alcohols as well as some alkylating agents, ethylene oxide, and acrylamide (Sec. “Acetaldehyde and Ethanol” to Sec. “Formaldehyde”). They are grouped together under the term “the aggregate exogenous and endogenous exposome”.

The second section of part B “Selected examples” (Sec. “Aflatoxin B1” to Sec. “Pyrrolizidine alkaloids”) addresses compounds humans are exclusively exposed to by exogenous exposure. The third section of part B “Selected examples” (Sec “Carcinogenic metal compounds: Examples cadmium and arsenic”) is devoted to carcinogenic metal compounds with special emphasis on cadmium and arsenic.

## Part B: Selected examples

In the following, selected genotoxic compounds are described in more detail, covering a spectrum of carcinogens with different potency and MOAs, including metals. They comprise (1) substances exerting exogenous exposure on top of a significant component of endogenous background exposure, (2) those for which endogenous exposure is not known but exerting lifestyle-associated, often unavoidable exogenous background exposure, such as aflatoxins, benzo[a]pyrene (BAP), heterocyclic aromatic amines (HAAs), allylbenzenes, pyrrolizidine alkaloids, and (3) selected metals, acting mostly by indirect genotoxic MOAs. These examples have been selected based on the availability of data on aggregate exposure levels and exposure biomarkers, on biological effects and on underlying MOAs. Furthermore, gaps in knowledge and research needs are defined.

### Presence of endogenous background levels of the same or similar DNA lesions

#### Acetaldehyde and ethanol

##### Introduction

Besides their occurrence in food and beverages and possible exposure at the workplace, ethanol and acetaldehyde are also endogenous substances with concentrations of about 2.2–6.5 µM (ca. 0.1–0.3 mg/l) in blood for ethanol (Greim 1998) and 2.2–3.6 µM (ca. 0.1 mg/l) in blood for acetaldehyde (Greim 2008). Endogenous acetaldehyde is produced in the intermediary metabolism by oxidative decarboxylation of pyruvate, in the course of amino acid metabolism, and by other metabolic processes. It is also formed by intestinal bacteria. Most of it is converted to ethanol by alcohol dehydrogenase (ADH) (Greim 2008).

Chronic alcohol consumption is associated with several forms of cancer, especially of the upper aerodigestive tract (Yu et al. 2010). The most important mechanism for the carcinogenicity of ethanol seems to be the formation of acetaldehyde as its primary metabolite (Brooks and Theruvathu 2005). Based on epidemiological data, the International Agency for Research on Cancer (IARC) concluded that there is sufficient evidence in humans for the carcinogenicity of alcohol consumption and for the carcinogenicity of acetaldehyde associated with the consumption of alcoholic beverages, and both were classified in category 1 (IARC 2010a; IARC 2012b).

In several animal studies with oral administration of ethanol, increased incidences of cancers of the head and neck and the liver and benign tumours of several organs in rats and liver tumours and mammary gland adenocarcinomas in mice were found (IARC 2012b).

In two studies with inhalation exposure in rats and hamsters, acetaldehyde showed carcinogenic effects in nose and larynx (Greim 2008). There is only one carcinogenicity study in rats with the oral application of acetaldehyde. Increased incidences of several tumours were observed, but there was no obvious dose–response relationship (IARC 2012b).

The German Commission for the Investigation of Health Hazards of Chemical Compounds in the Work Area (MAK Commission) classified ethanol and acetaldehyde in category 5, which is defined for substances with carcinogenic and genotoxic effects which are considered to contribute very slightly to human cancer risk, provided the MAK and BAT values are observed (DFG 2018; Greim 1998). At the MAK value for ethanol of 200 ml/m^3^ (≙ 380 mg/m^3^) the average life-time body burden of ethanol is still within the range of variation of the endogenous body burden. The commission, therefore, concluded that workplace exposure to concentrations up to 200 ml/m^3^ would contribute only little to cancer risk. A similar approach was chosen for acetaldehyde, where exposure levels up to the MAK value of 50 ml/m^3^ (≙ 91 mg/m^3^) were estimated to lead to an additional body burden in the range of variation of the life-time endogenous body burden. Prevention of nasal tissue irritation was also considered for the derivation of the MAK value for acetaldehyde.

##### Genotoxicity and DNA adduct formation

Acetaldehyde is genotoxic in vitro and in vivo. In vitro tests for the induction of SCE, chromosomal aberrations and micronuclei as well as gene mutation tests gave positive results. In vivo tests for the induction of SCE and micronuclei were also positive (Greim 2008). Ethanol exerted a weak genotoxic potential in vitro with metabolic activation and in vivo (Greim 1998). These effects are assumed to be predominantly related to acetaldehyde formation, but there are controversial reports about the genotoxicity of ethanol itself (Kayani and Parry 2010).

**Fig. 4:**
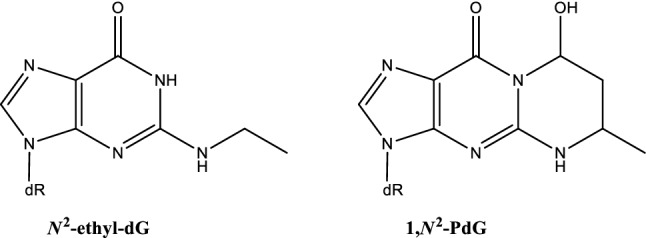
Structures of *N*^2^-ethyl-2′-deoxyguanosine (*N*^2^-ethyl-dG) and α-methyl-γ-hydroxy-1,*N*^2^-propano-2′-deoxyguanosine (α-Me-γ-OH-PdG; 1,*N*^2^-PdG) (according to Brooks and Theruvathu 2005)

Several DNA adducts are formed in vitro and in vivo after exposure to acetaldehyde. The main adduct is *N*^2^-ethylidene-2′-deoxyguanosine (*N*^2^-ethylidene-dG), which is unstable in hydrolysed DNA (Wang et al. 2006). Therefore, it is chemically reduced during the analytical procedure to its stable reduction product *N*^2^-ethyl-2′-deoxyguanosine (*N*^2^-ethyl-dG) (Fig. 4), which has been used as a biomarker for acetaldehyde-induced DNA damage, but its biological significance for the carcinogenicity of acetaldehyde remains unclear (Brooks and Theruvathu 2005; Brooks and Zakhari 2014; Yu et al. 2010). In contrast, the α-methyl-γ-hydroxy-1,*N*^2^-propano-2′-deoxyguanosine (α-Me-γ-OH-PdG; 1,*N*^2^-PdG) (Fig. 4) is considered a biologically relevant acetaldehyde-derived adduct, because it has also been identified as being responsible for the mutagenic, genotoxic and carcinogenic properties of crotonaldehyde (Eder and Budiawan 2001). Its formation is stimulated by the presence of histones and cellular polyamines (Brooks and Theruvathu 2005). Additionally, the ring-opened form of 1,*N*^2^-PdG is a precursor lesion for the formation of DNA–protein or DNA-DNA cross-links (Yu et al. 2010). The formation of the 1,*N*^2^-PdG adduct from acetaldehyde has been shown in vitro and in vivo (Garcia et al. 2011; Sanchez et al. 2018). Site-directed mutagenesis studies have shown that both the *N*^2^-ethyl-dG and the 1,*N*^2^-PdG adducts have mutagenic potential (Choi and Guengerich 2006; Stein et al. 2006; Terashima et al. 2001; Upton et al. 2006a, 2006b). Further identified acetaldehyde-derived adducts are *N*^2^-(3-hydroxybutyl)-dG and *N*^2^-(4-hydroxybutyl)-dG, *N*^2^-(2,6-dimethyl-1,3-dioxan-4-yl)-dG (*N*^2^-Dio-dG) and 3-(2-deoxyribos-1-yl)-5,6,7,8-tetrahydro-8-(*N*^2^-deoxyguanosyl)-6-methylpyrimido-[1,2-α]purine-10(3H)one (Yu et al. 2010).

##### Dose–response data for DNA adduct formation, mutagenicity and clastogenicity in vitro

In vitro studies were conducted with human lung fibroblasts (IMR90) and a human buccal epithelial cell line (SVpgC2a). In the latter, a dose-dependent increase in *N*^2^-ethyl-dG adducts was measured (by reduction) after incubation with up to 100 mM acetaldehyde for 1 h (Vaca et al. 1998). In the lung fibroblasts, exposure against acetaldehyde for 3 h led to increased levels of 1,*N*^2^-PdG adducts (Garcia et al. 2011).

In a study with a human leukaemia cell line (HL60), after incubation with 1.8 mM acetaldehyde for 1 or 2 h, *N*^2^-ethylidene-dG adducts were analysed (after reduction to *N*^2^-ethyl-dG). In the cells exposed for 1 h, the mean (± SD) levels immediately, 24 h, and 48 h after exposure were 121, 82, and 67/10^8^ nt, respectively, indicating about 50% repair within 48 h. In the control group, the mean levels were about 33/10^8^ nt at all time points. In the cells exposed for 2 h, the levels were 214, 105, and 98/10^8^ nt, respectively. A half-life of *N*^2^-ethylidene-dG adducts of 35 h was calculated (Hori et al. 2012).

Further studies with human lymphoblastoid TK6 cells using [^13^C_2_]-acetaldehyde in the range of 50 nM to 2 mM, incubated for 12 h, revealed an increase in exogenous *N*^2^-ethylidene-dG formation (after reduction to *N*^2^-ethyl-dG) at exposure concentrations ≥ 1 μM, whereas the endogenous adducts remained nearly constant across all exposure concentrations, with an average of 6.6 adducts/10^8^ nt. Levels of exogenous adducts were lower than endogenous adducts at concentrations ≤ 10 μM and were higher than endogenous adducts at concentrations ≥ 250 μM. The sum of endogenous and exogenous adducts reached a statistically significant increase over the endogenous background at 50 µM. Statistically significant decreases in cell survival and increases in micronucleus formation occurred at ≥ 1000 μM acetaldehyde (Moeller et al. 2013).

In another study with TK6 cells, acetaldehyde induced a concentration dependent, statistically significant increase in apoptotic cells and in micronuclei formation beginning at 0.25 mM. In the low concentration range up to 0.05 mM, these effects did not occur. Similar results were obtained in the TK gene mutation assay, with acetaldehyde inducing a significantly increased mutation frequency at 0.05 mM and above. In the HPRT gene mutation test no significant increase in mutation frequency could be detected (Budinsky et al. 2013).

##### Dose–response data for DNA adduct formation in experimental animals and the impact of genetic polymorphisms

The impact of aldehyde dehydrogenase (Aldh) genotype on adduct formation was tested in *Aldh2*-proficient (+ / +), heterozygous ( ±) and knockout ( – / – ) mice. After repeated ethanol intake (about 23 g/kg bw and day for 5 weeks) *N*^2^-ethyl-dG adducts (after reduction) in liver and stomach increased genotype-dependently up to 40-fold in *Aldh2*-knockout animals and about tenfold in heterozygous mice compared to untreated control animals. In *Aldh2*-proficient mice, ethanol intake leads to an up to fourfold increase in *N*^2^-ethyl-dG adducts. In untreated mice, no significant differences in *N*^2^-ethyl-dG adducts between the different genotypes were detected. There were no treatment-dependent changes in 1,*N*^2^-PdG adduct levels. *N*^2^-ethyl-dG adducts (without reduction) were not detected in any sample (Matsuda et al. 2007; Nagayoshi et al. 2009).

In another study in mice, basal mean *N*^2^-ethyl-dG adduct levels (after reduction) were about two times higher in the oesophagus and the tongue of *Aldh2*-knockout mice compared with *Aldh2*-proficient animals. In the submandibular gland, basal levels of *N*^2^-ethyl-dG adducts in *Aldh2*-knockout animals were relatively low compared with those of* Aldh2* proficient mice. Treatment with 8% ethanol in drinking water for 14 months resulted in increased adduct levels, which were considerably higher in *Aldh2*-knockout compared to *Aldh2*-proficient mice in all three tissues examined (Yu et al. 2012).

In Rhesus monkeys, the self-administered average ethanol consumption of 2.3 ± 0.8 g/kg bw and day for one year resulted in average *N*^2^-ethyl-dG levels (after reduction) of 9.4/10^8^ nt in oral mucosa (control: 3.3/10^8^ nt) and 4.5/10^8^ nt in oesophageal mucosa (control: 2.9/10^8^ nt). In mammary gland tissue of female animals exposed to ethanol, no increase in average *N*^2^-ethyl-dG levels was found. The correlation between *N*^2^-ethyl-dG levels in oral mucosa DNA and amounts of alcohol consumed per day was investigated. Levels of the DNA adducts increased with amounts of alcohol consumed even though the trend was not significant. The presence of 1,*N*^2^-PdG was also investigated in the oral and oesophageal mucosa DNA samples. No quantifiable levels of this DNA adduct were found in the samples analysed except for the oral mucosa sample of one animal, which had the highest level of *N*^2^-ethyl-dG adducts (33/10^8^ nt) (Balbo et al. 2016).

In an inhalation study with Aldh2-proficient (+ / +) and knockout ( – / – ) mice, the animals were exposed to 0, 125 or 500 ml acetaldehyde/m^3^ 24 h per day for 14 days. Formation of *N*^2^-ethylidene-dG was analysed after reduction to *N*^2^-ethyl-dG. In the liver, adduct levels in the knockout mice were always lower compared with the *Aldh2*-proficient animals, but these differences were not statistically significant. However, *Aldh2*-knockout mice showed significantly higher adduct levels than *Aldh2*-proficient mice in the nasal epithelium at 125 ml/m^3^ and in dorsal skin at 500 ml/m^3^, the other concentrations were not analysed in these tissues. In the lung, there was a statistically significant increase in adduct levels only at the high concentration of 500 ml/m^3^ with 171/10^8^ bases (control 43.3/10^8^ bases) for *Aldh2*-proficient and 283/10^8^ bases (control 65.7/10^8^ bases) for Aldh2 knockout mice (Oyama et al. 2010). Micronucleus frequencies in reticulocytes were significantly increased after exposure to 125 or 500 ml acetaldehyde/m^3^ and after oral administration of 100 mg acetaldehyde/kg bw for 2 weeks in *Aldh2*-knockout mice only (Kunugita et al. 2008).

In an abstract, a dose-dependent increase in 1,*N*^2^-PdG adduct levels in the lungs of rats after inhalation exposure to acetaldehyde (12–96 ppb) for 50 days is reported (Garcia et al. 2014). However, no full study report has been published so far.

Wistar rats were continuously exposed by inhalation to 0 or 10 ppb (14 µg/m^3^) of [^13^C_2_]-acetaldehyde for 50 days. Unlabelled endogenous 1,*N*^2^-PdG adducts were detected in the liver, brain and lungs in both groups. Due to the small sample size (*n* = 5), the quantification resulted in a high inter-individual variation and no significant differences between the groups were reported. The [^13^C_2_]-1,*N*^2^-PdG adduct (from the addition of one molecule of labelled acetaldehyde and one unlabelled molecule) was detected in a percentage similar to the natural abundance of the isotope. Labelled exogenous [^13^C_4_]-1,*N*^2^-PdG adducts (from the addition of two molecules of labelled acetaldehyde) were detected in the brain and lungs of [^13^C_2_]-acetaldehyde exposed animals, but the adduct levels were below the limit of quantification (Sanchez et al. 2018).

Taken together, the studies with oral uptake of ethanol in mice and monkeys showed an increase of *N*^2^-ethyl-dG adducts in the liver, stomach, and several tissues of the oral cavity. Inhalation exposure of mice and rats to acetaldehyde led to an increase of *N*^2^-ethyl-dG adducts in the lungs of mice and to the formation of 1,*N*^2^-PdG adducts in the brain and lungs of rats. Aldehyde dehydrogenase deficient animals are more susceptible to the generation of *N*^2^-ethyl-dG adducts after oral ethanol uptake and inhalation exposure to acetaldehyde.

##### DNA adduct levels and DNA adduct formation in humans

Basal levels of *N*^2^-ethyl-dG adducts (after reduction) have been quantified in white blood cells of human volunteers as well as in human liver and lung tissue. In the blood cells of drinkers *N*^2^-ethyl-dG adduct levels were increased, whereas in the lung tissue of smokers no significant differences were detected compared with non-smokers (Balbo et al. 2008).

The 1,*N*^2^-PdG adduct was detected in low levels in 4 of 23 human liver samples as well as in 16 of 45 human lung samples (Zhang et al. 2006).

In a group of 30 male non-smoking volunteers, levels of *N*^2^-ethyl-dG adducts (after reduction) in leukocyte DNA were analysed before and after the consumption of 150 mL of vodka (containing 42% ethanol). Baseline adduct levels were 34.6 ± 21.9/10^8^ nt. Average levels of *N*^2^-ethyl-dG observed at different time points up to 48 h following ingestion of alcohol were not statistically significant from the baseline level (Singh et al. 2012).

In a study with 10 human volunteers, the kinetics of formation and repair of *N*^2^-ethyl-dG adducts (after reduction) was investigated after consumption of alcohol corresponding to blood alcohol levels of 0.03%, 0.05% and 0.07%. Average basal levels of *N*^2^-ethyl-dG adducts in DNA extracted from granulocytes and lymphocytes were determined. *N*^2^-ethyl-dG adduct levels increased in all subjects after most of the doses. The increase was up to fivefold in granulocytes and lymphocytes and up to 100-fold in human oral cells. Peak levels were reached within 40 h in peripheral blood cells and within 4 to 6 h in the oral cells. The authors concluded that the observed substantial intraindividual variability indicates other important sources of this DNA adduct (Balbo et al. 2012a, b).

##### Local effects of ethanol and acetaldehyde in risk assessment

Ethanol is also metabolised to acetaldehyde by oral microbes and mucosal cells. Because of inefficient detoxification due to locally different enzymatic capacities, acetaldehyde accumulates in saliva and gastric juice (Homann 2001). Salivary acetaldehyde concentrations were found to be much higher than the blood acetaldehyde concentrations after ingestion of alcoholic beverages (Yokoyama et al. 2008). Additionally, deficient activity in ALDH2 plays an important role in increasing the risk for upper digestive tract cancer. Concentrations of acetaldehyde in saliva and gastric juice of ALDH2-deficient persons is 2 times and about 5 times higher than in ALDH2-proficient persons, respectively (Lachenmeier and Salaspuro 2017; Maejima et al. 2015). Thus, for the risk assessment of ethanol and acetaldehyde, local concentrations and effects have to be considered additionally to systemic effects.

##### Additional mechanisms affecting genomic stability

There is some evidence that acetaldehyde inhibits the activity of the direct DNA repair enzyme *O*^6^-methylguanine methyltransferase (MGMT) and also of DNA methyltransferase (DNMT), which plays a role in epigenetic gene regulation by the methylation at C5 of cytosine. A further potential mechanism is the modification of genome function by direct adduction of histones (Brooks and Zakhari 2014).

##### Conclusion

A dose-dependent increase in adduct levels in vitro and in vivo was observed in several studies for *N*^2^-ethyl-dG adducts. No treatment-dependent changes in 1,*N*^2^-PdG adduct levels were found in mice after oral exposure to ethanol, whereas increased 1,*N*^2^-PdG adduct levels in the lungs of rats after inhalation of acetaldehyde have been reported. Site-directed mutagenesis studies have shown that both the *N*^2^-ethyl-dG and the 1,*N*^2^-PdG adducts have mutagenic potential. However, the biological significance of the identified adducts for mutagenicity and carcinogenicity of ethanol and acetaldehyde is still not fully elucidated, and additional mechanisms may also account for their carcinogenic effect. Further research is needed concerning the dose-dependent correlation of DNA adducts and mutagenicity. It has to be taken into account, that both ethanol and acetaldehyde are endogenous substances originating mainly from amino acid metabolism. Any additional intake from exogenous sources within the range of variation of the endogenous body burden will contribute only little to cancer risk. However, this should be verified on the level of DNA adducts since local concentrations of acetaldehyde as well as local levels of acetaldehyde-derived DNA adducts in the upper aerodigestive tract seem to play a major role in the development of cancer from ethanol and acetaldehyde. Furthermore, tissue irritation by acetaldehyde needs to be prevented as a promotional event in carcinogenicity.

#### Acrylamide

##### Occurrence and exposure

Acrylamide (AA) is used *inter alia* as an industrial chemical e.g. in the production of polyacrylamides. Furthermore, it is formed during the heating of food. Various mechanisms of AA formation in food have been discussed. The reaction of reducing carbohydrates with amino acids, in particular asparagine, during non-enzymatic browning (Maillard reaction) appears to represent the most important mechanism of formation of AA in foods (Guth et al. 2013). AA is mainly formed in carbohydrate-rich, heat-processed foods, such as for example French fries, potato chips/crisps and coffee (EFSA 2015; Guth et al. 2013). Chronic dietary exposure of adults was estimated to be on average between 0.4 and 0.9 µg/kg bw/day (95th percentile 0.6–2.0 µg/kg bw/day) and of children between 0.5 and 1.9 µg/kg bw/day (95th percentile 1.4–3.4 µg/kg bw/day) (EFSA 2015).

Of note, there is compelling evidence from human intervention studies using duplicate diet technology that in addition to exogenous (dietary) exposure there is clearly sustained endogenous exposure to AA formed metabolically in the human body. This endogenous baseline exposure is estimated to account for about 0.2–0.4 µg AA/kg bw/day, quite close to the average dietary exposure level. The source of this endogenous background is not clear at present but it may well originate from the metabolism of the intestinal microbiota, as has recently been reported for acrolein that appears to be generated at about tenfold higher level than acrylamide (Goempel et al. 2017; Goerke et al. 2019; Ruenz et al. 2016; Ruenz et al. 2019).

AA is classified as a genotoxic carcinogen. For the risk characterization based on neoplastic effects, the margin of exposure (MOE) approach was used by EFSA. As the reference point, the benchmark dose lower confidence limit for 10% extra tumour incidence (BMDL_10_) of 0.17 mg/kg bw/day was deduced from data on observed incidences of Harderian gland adenomas and adenocarcinomas in male B6C3F1 mice exposed to AA for two years in an NTP study (EFSA 2015). MOEs calculated are substantially lower than 10 000, ranging from 425 to 89 for the mean exposure estimates and from 283 to 50 for the 95th percentile exposure estimates, indicating a concern with respect to neoplastic effects.

##### Biotransformation

**Fig. 5:**
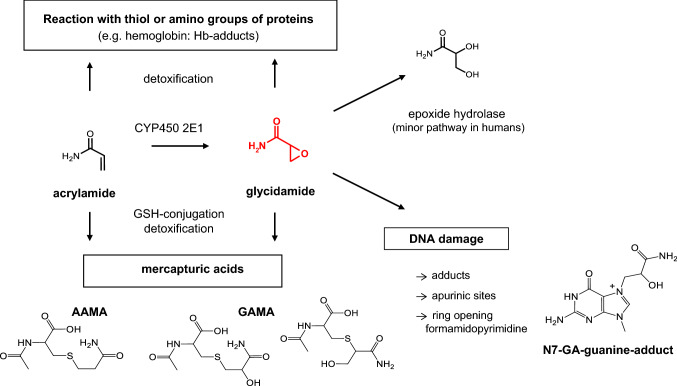
Metabolic pathway of AA in the rat. Reprinted (adapted) with permission from (Watzek et al. 2012). Copyright (2012) American Chemical Society

After ingestion and absorption from the gastrointestinal tract, AA is rapidly distributed and extensively metabolized, mainly by conjugation with glutathione (GSH) and reaction with other non-critical targets such as thiol or amino groups of proteins. To some extent, it is also converted by cytochrome P450 2E1 into the genotoxic epoxide glycidamide (GA) (Fig. 5). GA forms DNA adducts, primarily at N7 of guanine (N7-GA-Gua) which are found in experimental animals following AA exposure in various tissues. GA formation and its interaction with DNA are considered to represent the key event resulting in genotoxicity and carcinogenicity of AA (EFSA 2015). This has, however, to be reconciled with various experimental in vivo findings, indicating that formation of N7-GA-guanine DNA adducts at low exposure especially when approaching average present day consumer exposure levels, is not or only barely detectable. Further details see below and Watzek et al. (2012).

Conjugates of AA and GA with GSH after further biotransformation are excreted in urine as AA mercapturic acid (AAMA) and glycidamide mercapturic acid (GAMA) (Fig. 5). Comprehensive profiling in humans has also indicated the formation of a sulfoxide of AAMA with the abbreviation AAMA-sul (Wang et al. 2016).

Hepatic biotransformation was studied in primary rat hepatocytes, incubated with AA (0.2–2,000 µM) for up to 24 h. AA-GSH adducts became measurable much earlier than the genotoxic metabolite GA. The rate of AA-GSH formation was found to be about 1.5–3 times higher than that of GA formation. N7-GA-Gua adducts in primary hepatocyte DNA were found only at the highest AA concentration tested (2 mM) and at extended incubation times (6 h: 13 ± 3; 16 h: 127 ± 60 adducts/10^8^ nucleotides). Concomitant with reduced AA-GSH formation, GA content in the incubation medium increased and N7-GA-Gua adduct formation became measurable (Watzek et al. 2013).

Findings by several groups indicate that humans are less proficient than rodents in activating AA metabolically to GA and may be more proficient in detoxification reactions such as coupling to GSH (Berger et al. 2011; Fennell and Friedman 2005; Fuhr et al. 2006). In humans, only a minor part of an ingested dose is expected to account for the formation of GA, GAMA and glyceramide. These data suggest marked intra and interspecies differences concerning metabolism of AA to GA. Several studies have reported various approaches to physiologically based pharmacokinetic (PBPK) modelling of AA absorption, metabolism and disposition with the goal of predicting human internal exposures to AA and GA (i.e. area under the curve, AUC) and of reducing the uncertainty in risk assessment inherent in animal to human extrapolations (EFSA 2015).

##### Carcinogenicity-Observational studies

Epidemiological studies which analysed the association between AA exposure through diet or at the working place and the incidence or mortality from cancer have recently been reviewed by EFSA (EFSA 2015). EFSA concluded that the epidemiological studies did not indicate AA to be a human carcinogen, although the margins of exposure determined using the BMDL_10_ derived from animal tumor data indicated a concern. Furthermore, the two available epidemiological studies of occupational exposure to AA (Marsh et al. 2007; Swaen et al. 2007) both did not indicate an increased risk of cancer.

##### Animal studies

AA is carcinogenic in multiple tissues in both male and female mice and rats. In long-term studies in rats, a carcinogenic potential of AA was demonstrated after administration via drinking water at doses of 0.5–2 mg/kg bw/day in male animals or at doses of up to 3 mg/kg bw/day in female animals. Enhanced occurrence of certain tumours such as mesotheliomas of the tunica vaginalis testis, mammary fibroadenomas and thyroid tumours (follicular adenomas) has been reported (reviewed by Guth et al. 2013). The carcinogenic potential of AA was confirmed in a two-year NTP study (NTP 2012). In rats and mice tumours were found in different organs, some of them already at the lowest AA dose of 0.33–0.44 mg/kg bw (male and female rats) or 1.04–1.1 mg/kg bw (male and female mice), so that no threshold could be deduced in this study (Guth et al. 2013; NTP 2012). Of note, Fisher rats are known for their susceptibility to Leydig cell tumors and secondary induction of tunica vaginalis mesotheliomas, in contrast to the Wistar strain (Maronpot et al. 2009; Shipp et al. 2006). In a guideline compliant two year study that included in utero exposure, pregnant, preweanling Wistar Han rats and the F1 offspring animals were given AA in a dose range of 0.5–3.0 mg/kg bw in drinking water, starting at gestation day 6. Potentially treatment-related tumors, as previously observed in earlier studies in Fisher 344 rats, were not observed in the Wistar strain. At the end of two years mammary gland fibroadenomas were observed in females (not significant and within published control range for Wistar rats) as well as thyroid follicular cell tumors in both sexes (Maronpot et al. 2015). For mammary fibroadenomas in rats, the luteotrophic effect of age-associated prolactinaemia is supposed to be causative. This MOA is considered not likely relevant to women where prolactin is not luteotrophic. Likewise, a role for hormonal dysregulation affecting the pituitary–thyroid axis and the rat specific thyroid homoeostasis is generally supposed as likely cause for follicular cell neoplasia in rat carcinogenicity studies and is considered a rat-specific response (Alison et al. 1994; Bartsch et al. 2018; Capen 1997; Capen and Martin 1989; Khan et al. 1999; Maronpot et al. 2015; Neumann 1991). In summary, these target tissue-specific neoplastic responses are accepted to represent rat-specific MOAs, not likely predictive for human cancer risk (Maronpot et al. 2015).

In mice, the major tumours produced by AA in females and males were Harderian gland adenomas and adenocarcinomas, lung alveolar and bronchiolar adenomas, kidney tumours and stomach and forestomach squamous cell papillomas. In females also mammary gland adenoacanthomas and adenocarcinomas, benign ovary granulosa cell tumours and skin sarcomas have been observed. A BMDL_10_ of 0.17 mg/kg bw was calculated from induction of Harderian gland tumours in mice, the most sensitive lesion out of a spectrum of AA-induced rodent tumours (NTP 2012). Based on this BMDL_10_, EFSA derived MOEs ranging from 283 to 50 for the 95th percentile average exposure estimates. In view of such a low MOE range, EFSA expressed a human health concern (EFSA 2015).

A similar spectrum of tumours has been observed in rats and mice when equimolar concentrations of GA were administered in drinking water. It was concluded that the carcinogenic activity of AA is due to its metabolic conversion to GA (EFSA 2015; NTP 2012).

##### Genotoxicity in vitro

Genotoxic activity of GA was investigated in comparison to that of other activated forms of well-known carcinogens in the single-cell gel electrophoresis (Comet assay) in V79 cells and in human lymphocytes. GA induced DNA damage down to 300 µM concentration (4 h) (Baum et al. 2008). By comparison, the preactivated N-nitroso compound 3-N-nitroso-oxazolidine-2-one (NOZ-2) and ( ±)-anti-benzo[a]pyrene-7,8-dihydrodiol-9,10-epoxide ( (±)-BPDE), were much stronger genotoxic agents, significantly inducing DNA damage already at 3 µM (15 min) (Baum et al. 2008). In the *hPRT* mutagenicity test in V79 cells, GA induced mutations only at concentrations of 800 µM and above, whereas NOZ-2 as well as ( ±)-BPDE significantly induced *hPRT* mutations already at > 200-fold lower concentration (Baum et al. 2005, 2008; Thielen et al. 2006).

A comparison of the mutagenic potential of AA and GA in the hPRT assay in V79 cells to that of N-methyl-N'-nitro-N-nitroso-guanidine (MNNG) as positive control showed marked mutagenic effectivity already at 0.5 µM for MNNG, whereas AA was inactive up to a concentration of 10 mM (Baum et al. 2005). GA showed a concentration-dependent induction of mutations at concentrations of 800 µM and higher. In another experiment, human blood was used as a model system to investigate genotoxic potential in lymphocytes by the comet assay and by measuring the induction of micronuclei (MN) with bleomycin (BL) as a positive control. AA did not induce significant genotoxicity or mutagenicity up to 6 mM (Baum et al. 2005). With GA, concentration-dependent DNA damage was observed in the dose range of 300–3000 µM after 4 h incubation. Significant MN-induction was not observed with AA (up to 5 mM) and GA (up to 1 mM), whereas BL induced significantly enhanced MN frequencies at 4 µM (Baum et al. 2005). Taken together these results revealed AA not to be genotoxic/mutagenic whereas GA can be considered a rather weak genotoxic mutagen, especially when compared to established mutagens and carcinogens like activated nitroso compounds or polycyclic aromatic hydrocarbons.

The finding that GA exerts a rather modest genotoxic and mutagenic activity may be a result of its preferential N7-guanine alkylation. N7-guanine alkyl adducts are considered to exert only low or even no mutagenic effects as such.

##### Genotoxicity in vivo

AA was given to rats at a daily intake level in AA-containing foods for up to 9 days, resulting in an exposure of 50 or 100 μg AA/kg bw/day (Berger et al. 2011). Positive controls received the same dosages of AA in water, negative controls just water. As biomarkers urinary mercapturic acids (MA), haemoglobin (Hb) adducts, plasma levels of AA and GA and induction of DNA damage in white blood cells and hepatocytes were measured. Significant differences in overall bioavailability of AA from water and the different food matrices were not observed. Hb adducts of AA followed time/exposure-related dose–response. In contrast, Hb adducts of GA were not enhanced above untreated control, although GAMA excretion in urine indicated significant GA formation. This suggests that at these dose levels any GA formed metabolically in the liver is effectively scavenged by glutathione coupling (Berger et al. 2011).

**Fig. 6:**
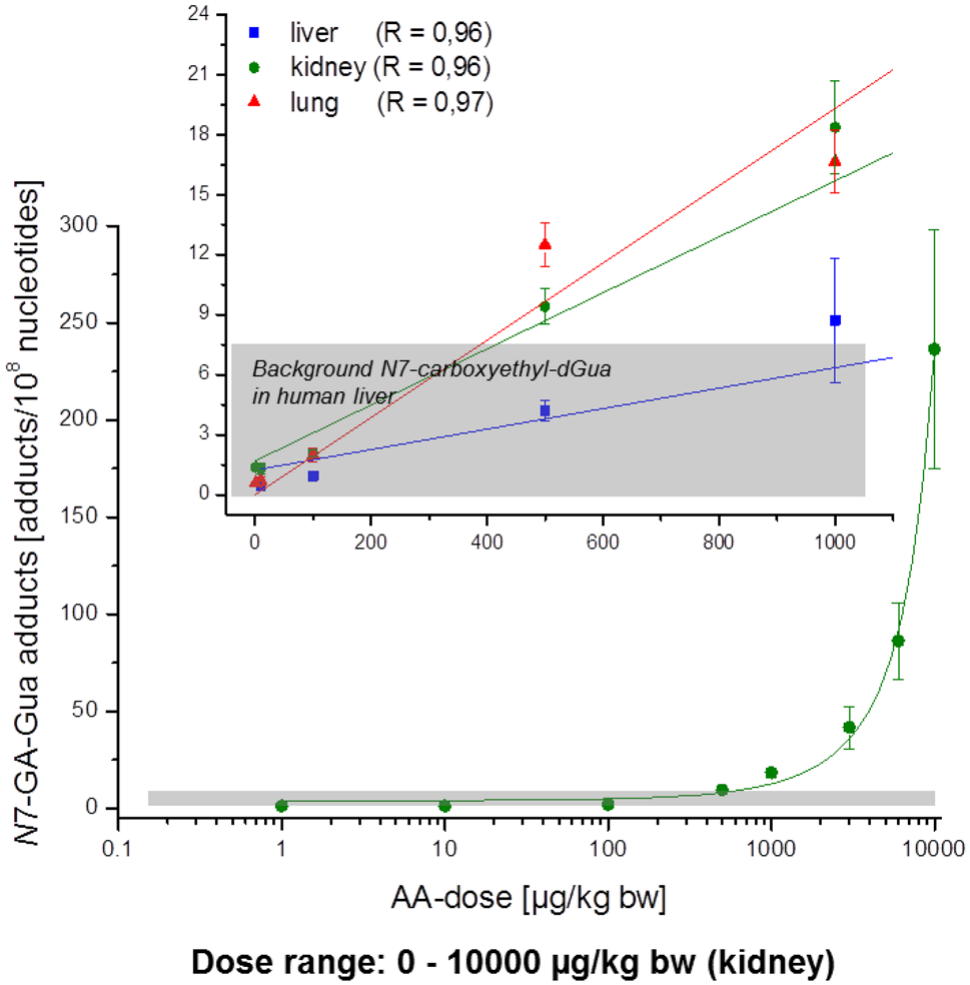
Dose–response relation of N7-GA-Gua adducts in rat kidney orally exposed to 0.1–10,000 μg AA/kg bw (*R*^2^ = 0.99) (only kidney shown in the linear-log plot for graphic clarity). Insert, linear–linear plot of the low dose range (0.1–1000 μg AA/kg bw); shaded, range of DNA background of N7-carboxyethyl-dGua in human liver (see also part A "Fundamental considerations", chapter “background DNA lesions”). Values represent mean values ± SDs (*n* = 8 or *n* = 3). Reprinted with permission from (Watzek et al. 2012), Copyright (2012) American Chemical Society

In a further dose–response study AA was given orally in a single dosage of 0.1–10,000 μg/kg bw to female Sprague-Dawley (SD) rats (Watzek et al. 2012). Formation of urinary mercapturic acids and of N7-GA-Gua DNA adducts in liver, kidney, and lung were measured 16 h after application, which had previously been determined as the time point of maximal N7-GA-Gua DNA concentration (Fig. 6). At this time point, urinary excretion of mercapturic acids is not complete yet. A mean of 37.0 ± 11.5% of a given AA dose was found as mercapturic acids in urine. MA excretion in urine of untreated controls indicated some background exposure from endogenous AA. N7-GA-Gua adduct formation was not detectable in any organ tested at 0.1 μg AA/kg bw. At a dose of 1 μg/kg bw, adducts were found in kidney (around 1 adduct/10^8^ nucleotides) and lung (below 1 adduct/10^8^ nucleotides), but not in liver. At 10 and 100 μg/kg bw, adducts were found in all three organs, at levels close to those found at 1 μg AA/kg, covering a range of about 1–2 adducts/10^8^ nucleotides. In the dose range from 0.1–100 µg/kg bw/d no linear dose–response relationship was apparent (Fig. 6). DNA adduct levels from electrophilic genotoxic agents of various origin were found in human tissues at levels of up to about 200 specific adducts/10^8^ nucleotides (Nakamura et al. 2014; Swenberg et al. 2011) (see also part A "Fundamental considerations", chapter “background DNA lesions”). An adduct considered closely related to N7-GA-guanine, N7-carboxyethyl dGua, was found at a background level of about 8 adducts/10^8^ nucleotides (Watzek et al. 2012). By comparison, N7-GA-Gua adduct levels within the dose range of 0.1–100 μg AA/kg bw were at the low end of human background. At the reported BMDL_10_ (0.17 mg AA/kg bw/day in mice), N7-GA-guanine adduct levels can be expected from this single dose–response study to not exceed around 3–4 adducts/10^8^ nucleotides in rats, still not exceeding comparable human background (Watzek et al. 2012).

Altogether these data do not provide compelling support for genotoxicity of AA being relevant for cancer endpoints in experimental animals. Likewise, novel toxicogenomic evidence obtained from studies in F344 rats given AA at dosages up to 12.0 mg/kg bw/day for different subchronic time periods does not convincingly support a genotoxic or hormonal MOA. Instead, pronounced effects on calcium signalling and on cytoskeletal functions were observed in the thyroid, a tumour target organ in the male rat (Chepelev et al. 2017). Under the same experimental conditions, evidence for a similar MOA being operative in rat testes has been reported (Recio et al. 2017). In vivo mutagenicity studies using the Pig-a gene mutation assay and the micronucleus test in F344 rats and in B6C3F1 mice under similar conditions gave negative to equivocal results. In addition, at dosages < 6.0 mg/kg bw/day no in vivo mutagenicity was observed. This is in agreement with the perception of a non-genotoxic MOA being relevant for AA-induced tumorigenicity in the carcinogenic dose range in rodents (Hobbs et al. 2016).

##### Impact of AA on transcription and gene expression profiles

Several toxicogenomic studies on rats and mice exposed to AA in a time- and dose-dependent manner were conducted (Chepelev et al. 2017, 2018; Recio et al. 2017). The group performed mRNA gene expression profiling to develop a MOA and to calculate transcriptional BMDLs for the most sensitive pathways, comparing these with data derived from traditional (apical) cancer studies. Concerning the MOA, they observed no altered gene expressions of pathways associated with a genotoxic MOA (i.e. p53, DNA repair, cell cycle). Moreover, the findings pointed to an alternative MOA comprising perturbation of the calcium signalling as a key event in the AA-mediated carcinogenesis in rodents. Taken together, these results are in line with those mentioned above and not supportive of a key role of genotoxicity in the MOA of AA. The comparison of transcriptional-derived PODs with those obtained from 2-year rodent studies revealed that the transcriptional BMDLs were around one to threefold of apical BMDLs.

##### Conclusion

The totality of the evidence does not compellingly support the note that AA induces malignant transformation in animal experiments by virtue of a genotoxic MOA. AA itself clearly is non genotoxic, but can be converted metabolically to the epoxide GA which may exert DNA damage by covalent binding. Such genetic damage may result in fixed mutations, eventually leading to neoplastic transformation. Although this has been favoured in the past as most probable key event of AA-induced neoplastic transformation, compelling evidence is lacking. The genotoxicity of AA may be understood as a high dose effect but the overall evidence suggests a non-genotoxic MOA even at elevated dose levels. Summarizing major arguments, it is concluded that:

1. the presumed genotoxic key metabolite GA is a rather poor mutagen, predominantly inducing N7-GA-Gua lesions, known to be of rather low promutagenic activity (Baum et al. 2008; Durling and Abramsson-Zetterberg 2005; Glatt et al. 2005; Johansson et al. 2005; Pottenger et al. 2009a; Puppel et al. 2005; Thielen et al. 2006). Accordingly, in vivo mutagenicity experiments in rodents at dosages < 6.0 mg/kg bw/day were reported not to induce a mutagenic response (Hobbs et al. 2016).
2. in vivo, at realistic low exposure levels encompassing diet-related intake, AA induces only very minor DNA damage in rats. At single dosages up to at least 100 μg/kg bw, adduct formation was not found dose-related and stayed within the human background of similar DNA lesions at N7-dG (Watzek et al. 2012).
3. evidence is accumulating from toxicogenomic studies arguing for MOAs other than genotoxicity. This applies especially to the target organs of AA in rodent carcinogenicity studies, such as the thyroid, the testes, and the Harderian gland, where pronounced effects on calcium signalling and on cytoskeletal functions have been observed, but no compelling support for a genotoxic or hormonal MOA was found (Chepelev et al. 2017, 2018; Recio et al. 2017).

Thus, the totality of evidence appears to argue for the existence of an AA exposure level not associated with induction of toxic or adverse health effects. If established, it may enable the definition of a tolerable daily intake level (TDI).

##### Gaps in knowledge and research needs

Amongst the process-related food contaminants, AA has contracted major attention of the research community and of major risk assessing bodies, under the perception that AA is a process-related toxicant acting by a genotoxic mechanism of action. Recommendations by risk management bodies were developed to limit consumers’ exposure as far as reasonably achievable (ALARA principle). Under the premise of AA being a genotoxic carcinogen, present day MOE ranges appear unsatisfactory. However, in view of the accumulating evidence for a non-genotoxic MOA, TDIs may be considered.

For future risk assessment gaps in knowledge concerning the MOA of AA at realistic exposure levels need to be closed and the endogenous background of sustained AA formation in humans needs to be taken in due consideration.

With the help of toxicogenomics, PBPK modelling, advanced biomarker monitoring and reverse dosimetry it should be possible to:

1. establish a compelling MOA for AA taken up at diet-related dosage (genotoxic/ non genotoxic)
2. create dependable correlations between exposure and biomarker levels
3. elucidate the relative importance of endogenously generated AA versus AA taken up by exogenous pathways
4. establish a comprehensive database on DNA background damage in humans

#### Alkylating agents

Alkylating agents are prototypic genotoxic carcinogens. The primary DNA modification consists of an alkylation of DNA bases, which typically lead to a base mispairing in the subsequent replication round and consequently a base-pair substitution mutation. Methyl and ethyl residues are typical transduced groups, and metabolic activation of these directly alkylating compounds is not required to render them reactive. Of the possible modifications alkylation of the *O*^6^ and N7 positions of guanine are of major importance, with methylation or ethylation of the *O*^6^ position being a particularly premutagenic lesion, despite the fact that this modification accounts for less than 8% of the total alkylations (Beranek 1990), in that it effectively leads to a GC→AT transition mutation. In mammalian cells, repair of this primary DNA modification occurs via a so-called suicide reaction by the enzyme methyl guanine methyl transferase (MGMT) (see part A Sec. “DNA reactivity of chemicals: Hazard versus Risk” `DNA reactivity of chemical substances´). The repair protein transfers the methyl group from the alkylated guanosine in a one-step reaction onto an internal cysteine residue in its active centre (Pegg et al. 1995), a reaction which leads to the inactivation of the enzyme which, therefore, needs to be regenerated. Obviously, since this reaction does not involve an additional DNA modification step it is essentially error-free. Further, since the DNA damage removal consists of basically a stoichiometric reaction of the repair protein with its substrate, followed by regeneration of the enzyme after inactivation by the reaction, a thresholded kinetic mechanism is readily conceivable considering the saturation of the repair may occur when all enzyme is inactivated (Müller et al. 2009).

Insight into the existence of an apparent threshold in this reaction was provided by an extensive series of experiments conducted by the pharmaceutical company F.Hoffman-La Roche, Basel, Switzerland. Through a manufacturing accident, in 2007 a batch of Viracept (nelfinavir mesylate), an antiviral drug used for HIV treatment, had been in contact with cleaning ethanol for a prolonged time period, by which elevated levels of ethyl methane sulfonate (EMS) were formed without being discovered before exposing patients to this material. Obviously, the distribution of this batch was immediately suspended but a thorough risk assessment for treated subjects was needed (Müller et al. 2009). EMS is an alkylating agent which leads to the formation of DNA ethyl adducts. The hypothesis formed was based on the above consideration, i.e. that of the intracellular MGMT level should provide for error-free repair up to a level of exhaustion of its pool, above which the repair kinetics can be expected to display a significantly different profile.

Key parameters to describe human exposure, based on therapeutic uptake of Viracept, were the following:

- Maximal content of EMS in Viracept: ca 2300 ppm
- Maximal content of EMS in tablets: ca 1000 ppm (TTC level: 0.6 ppm)
- → Maximal dose of EMS in patients: 0.055 mg/kg
- Maximal duration of exposure: 3 months
- Number of patients: < 40,000

The following experimental approaches were pursued:

1. A general repeated-dose oral toxicity study in rats with EMS, with the aim to establish basic knowledge of EMS organ toxicity and clinical chemistry/haematology in conjunction with toxicokinetic data to describe exposure.
2. Genotoxicity:
  (a). Induction of chromosomal damage in the bone marrow of mice (bone marrow micronucleus test) after repeat dosing (7 days) with EMS and ethyl nitrosourea (ENU), with the aim to study the dose–response behaviour of EMS at low doses for chromosome damage compared to that of ENU, of which the DNA damage is known to be not efficiently repaired by MGMT (Kaina et al. 1991). Dose levels applied were 1.25, 2.5, 5, 20, 80, 140, 200 and 260 mg/kg bw/day for EMS and 1.11, 4.45 and 17.8 mg/kg bw/day for ENU, based on dose-finding information.
  (b). Induction of LacZ gene mutations in MutaMouse (transgenic model) after 28-day oral dosing with EMS and ENU, with the aim to characterize the dose–response for EMS in low doses for gene mutations compared to the dose–response for ENU. Tissues analysed were bone marrow, liver and gastrointestinal tract, and dose levels applied were 1.56, 3.13, 5.25, 12.5, 25, 50 and 100 mg/kg bw/day for EMS and 1.39, 5.56 and 22.25 mg/kg bw/day for ENU. In addition, single doses of 350 mg/kg and 15.56 mg/kg of EMS and ENU, respectively, were administered to explore the difference between fractionated and combined administration of the same dose.
  (c). Exploration of data by statistical methods (Gocke and Wall 2009).
3. Determination of globin valine ethyl adducts in the bone marrow as a dosimetry parameter to confirm exposure to EMS.
4. Cross-species in vitro and in vivo evaluation of exposure to EMS (mouse, rat, monkey, human), with the aim to retrospectively facilitate exposure judgment in patients having been exposed to elevated levels of EMS via Viracept treatment.

In summary, under all circumstances (i.e. both with using bone marrow micronucleus frequencies as well as lacZ mutations in bone marrow, liver or gastrointestinal tract), dose–responses indicative of thresholded relationships were obtained for EMS-treated animals, supported by various statistical methods (Gocke and Wall 2009), while ENU showed a linear dose–response behaviour. Across organs and endpoints no-effect-levels were remarkably similar. Thus, daily doses of up to 25 mg EMS/kg bw/day (bone marrow, GI tract) or 50 mg EMS/kg bw/day (liver) did not induce mutations in the lacZ gene in the three organs tested. Doses up to 80 mg EMS/kg bw/day (7-day dosing regimen) did not induce micronuclei in mouse bone marrow (Gocke et al. 2009). Thus, using two different endpoints (point mutations and chromosome aberrations) revealed roughly comparable thresholds for the induction of an effect, and also across different organs. In contrast, the induction of ethyl valine adducts determined in haemoglobin, considered as surrogate biomarkers for DNA damage, followed linear kinetics with no obvious threshold, which is in line with previous reports that showed that the induction of DNA alkylations follows a linear, non-thresholded relationship (Swenberg et al. 2008).

When the same total EMS dose was administered at once rather than in portions, a much steeper dose–response was observed. For ENU, which was used as the comparator compound, no threshold was seen within the dose-range used, and dose fractionation and single-dose administration gave comparable dose–responses, which is an indication of that there is no exhaustible DNA repair system.

For EMS, safety margins to human exposure under the circumstances of treatment with the contaminated batch, using those threshold data, resulted in factors of 370 or 454, based on *C*_max_ or dose, respectively, which was considered sufficient evidence of the lack of a genotoxic and hence carcinogenic risk for the exposed persons.

Thresholded dose–response relationships have been described for EMS in vitro as well (Doak et al. 2007). In this study, human lymphoblastoid AHH-1 cells were treated with relatively closely spaced concentrations of EMS, MMS, MNU or ENU, and micronucleus frequencies were determined after cytokinesis block, or HPRT mutant frequencies to assess point mutations. In the micronucleus test, apparent thresholds ranging between 0 and 0.80 µg/ml MMS and 0–1.35 µg/ml EMS were seen. Above those concentrations, statistically significant increases in micronucleus frequencies were detected, resulting in effect concentrations of 0.85 and 1.40 µg/mL for MMS and EMS, respectively. For MNU and ENU, no thresholds were seen down to the lowest concentrations tested. Similarly, HPRT mutants were induced at 1.25 µg/mL MMS and 1.4 µg/mL EMS, but not below those concentrations, whereas for MNU and ENU again an increase in mutant frequency was seen down to the lowest concentration. Therefore, those data support the hypothesis, formulated within the Viracept investigations, that an essentially error-free DNA repair enzyme is able to protect cells and tissues against ethylation and methylation by EMS and MMS. However, both investigations were unable to identify an apparent threshold for the nitrosoureas ENU and MNU, indicating that different repair may account for the protection against those agents. In all cases, the onset of increases in genotoxic response was not correlated to a shift in cytotoxicity, so that this effect was excluded as the mechanism for a thresholded dose–response. Hypotheses formulated in this study imply that for alkyl methanesulfonate-induced DNA adducts, which consist mainly of N7-G adducts and less of *O*^6^-G adducts at low doses (Doak et al. 2007), BER may be responsible for removing the N7-alkyl-G lesions induced by MMS and EMS, whereas MGMT can cope with the low levels of *O*^6^-alkyl-G adducts, thus resulting in the observed threshold concentration ranges for the induction of chromosomal damage. MNU and ENU also induce N7-alkyl-G adducts that may be efficiently repaired by BER at low doses, but they also induce a comparatively larger proportion of *O*^6^-alkyl-G lesions that may be too extensive for the MGMT enzyme to correct even at low doses. In addition, they induce adducts at *O*^2^- and *O*^4^-Thymine, which are both very poorly repaired; thus, the unrepaired O adducts may result in linear concentration-responses for these adducts (Doak et al. 2007).

Using the PIG-A system in erythrocytes and reticulocytes in rats in vivo, Dobo et al. (2011) confirmed the value for the apparent threshold seen in the Viracept studies and, by extending ENU treatment to lower doses, also observed an apparent threshold for this mutagen (Dobo et al. 2011). EMS dose levels up to 100 mg/kg bw/day were used for 28 daily treatments, and up to 700 mg/kg for single doses. ENU was used for up to 15 mg/kg bw/day for a 28-day study, and up to 28 mg/kg acutely. In both cases, subacute vs. acute dosing was used to investigate the existence of an exhaustible damage removal system. For both compounds, a clear difference was seen in the numbers of induced mutations when a single high dose was compared to the same total dose administered in 28 fractions, in that the acute doses induced clearly higher mutant frequencies; again an indication of a saturable defence system. Thresholds for EMS treatment, i.e. doses up to which no statistically significant increase over the concurrent background value is observed, were comparable to the values determined in the Viracept study, despite the fact that different endpoints were used.

Further reports are available which are confirming apparent thresholds for alkylating agents. Thus, Jenkins et al. (2005) observed non-linear dose–response relationships for MMS treatment of cells in vitro and using ML-TK and HPRT mutations as well as micronuclei as the genotoxicity endpoints. Pottenger et al. (2009b), reported similar results for ML-TK mutations and DNA adducts in vitro.

##### Conclusion

In conclusion, there is sufficient evidence to indicate that for alkylating agents, which will induce genotoxic effects via direct interaction with DNA, low dose sublinear dose–response relationships do exist and can be described and quantified using various tests systems and endpoints. Hypotheses exist for the mechanisms of those apparent thresholds, the most prominent being saturable DNA repair systems, and there is little doubt that they are of biological relevance and should be taken into account when assessing the risk of low-dose exposure scenarios. However, open questions remain:

- What are the levels of DNA repair (e.g. MGMT) activities in different organs or species?
- What are the appropriate statistical methods to describe linearity or non-linearity?
- What is the relevance of the different endpoints, particularly DNA adducts vs. mutations?
- What should be a minimum data set to claim an apparent threshold?

#### Ethylene oxide

Ethylene oxide is both, a high volume compound in chemical industries and a direct-acting carcinogen. This combination asks for an utmost precise risk assessment. On top, the compound is also formed as an endogenous metabolite, raising the question whether the resulting natural exposure might serve as an orientation for the regulation of the occupational exposure.

##### General information

The estimated annual production volume of ethylene oxide is presently around 20 million tons, since 11% of the 150 million tons of ethylene, the organic entity with the highest annual production volume worldwide (True 2013), is used for its synthesis (Ceresana 2010). The majority of ethylene oxide is used as a chemical building block for the synthesis of other compounds. However, around 20% are applied for sterilization purposes, such as decontamination of medical instruments. At ambient temperature, the compound is a gas (boiling point 10.5 °C) and highly water-soluble, with a logP_o/w_ of  − 0.3, very similar to that of ethanol (PanGas 2012). This amphiphilic behaviour, in combination with the small molecular weight of 44, suggests easy penetration of the compound across membrane barriers and thus unlimited access to all body compartments, with a slight preference for aqueous compartments.

Ethylene oxide is electrophilically reactive, yet stable at room temperature in the absence of catalysts. Once initiated, it can undergo several types of substantially exothermic reactions, from polymerisation to spontaneous decomposition. As a gas, it is explosive in the wide range of concentrations from 2.6 to 100%. Under neutral conditions, its hydrolysis rate in aqueous solution is very slow (*t*_1/2_ = 20 days at 20 °C) but is strongly accelerated by acidification (Brönsted et al. 1929).

The odour detection threshold for ethylene oxide with 300–700 ppm is very high (Ruth 1986). Above this, the compound has a sweetish, ethyl ether-like smell. This is already in the acutely toxic range: in an NTP mouse study, 9 out of 10 animals died within the 4 h after the experiment when exposed to 800 ppm over a period of 6 h. Although not specified in this report a reasonable assumption is that the mice died from acute lung oedema. In a fourteen weeks study with repeated 6 h exposure, all animals of the 400 ppm exposure group died within the first 4 weeks (NTP 1987).

##### Kinetics and metabolism

Endogenous formation of ethylene oxide is due to CYP-dependent ethylene oxidation, mainly driven by CYP2E1 (Li et al. 2011b). Ethylene is an endogenous metabolite but is also taken up from external sources, such as food. Its metabolism to ethylene oxide is limited by saturation at exposure concentrations ≥ 1000 ppm in ambient air (Filser and Bolt 1984) and partial self-inactivation of CYP by ethylene oxide formation (Li et al. 2011b), which results in a maximum production rate equivalent to an ethylene oxide exposure of 4–8 ppm. This might explain that ethylene itself is not positive in carcinogenicity studies (Hamm et al. 1984), due to the limited sensitivity of such studies. In an attempt to estimate the lifetime risk for carcinogenesis due to ethylene-based endogenous ethylene oxide formation of approximately 1 nmol/hour for a 70 kg adult male (Filser et al. 1992), an approximate increase of 1 tumour per 10^4^ individuals has been communicated (Denk and Filser 1990).

One important metabolic route for ethylene oxide breakdown is glutathione conjugation (Li et al. 2011b), in man with a substantial contribution by the polymorphic GST T1 isoenzyme (Pemble et al. 1994). Consequently, respective metabolite concentrations in urine after exposure to ethylene oxide correlate with the GST T1 genotype (Haufroid et al. 2007), but also demonstrate the presence of other GST isoenzymes for ethylene oxide conjugation that produce mercapturic acid conjugates in the absence of a functional GST T1 gene. Hydrolysis of ethylene oxide by epoxide hydrolases is the second metabolic route (Brown et al. 1996; Li et al. 2011b). Its contribution to ethylene oxide elimination apparently plays a minor role in rodents while in humans it contributes substantially to ethylene oxide turnover. Physiologically based toxicokinetic modelling first predicted a ≥ 25% contribution of epoxide hydrolases to the overall human ethylene oxide metabolism (Li et al. 2011b) while a recently refined model increases this prediction to 85% (Filser and Klein 2018). Surprisingly, exhalation is not a major route of elimination after acute exposure, because less than 10% ethylene oxide is exhaled unchanged after systemic uptake (Csanady et al. 2000). The speed of enzymatic ethylene oxide metabolism differs significantly between mouse, rat and human. Consequently, the half-life of ethylene oxide is with approximately 3 min (mouse), 12 min (rat) and 45 min (human) substantially different between these three species (Brown et al. 1996; Fennell and Brown 2001). In mouse, continuous ethylene oxide exposure above 200 ppm leads to an over proportional increase in ethylene oxide blood levels explained by liver GSH depletion (Brown et al. 1998). Interestingly, different organs within the same animals were differentially affected, with lungs being more sensitive, i.e. displaying onset of GSH depletion already at 50 ppm and testis being less sensitive (onset of GSH depletion only at 300 ppm), as compared to liver.

##### DNA and protein adduct formation

Due to its electrophilic reactivity, ethylene oxide produces 2-hydroxyethyl adducts of DNA and proteins without metabolic activation. In vitro reaction of ethylene oxide with DNA led to the formation of at least five different adducts, the major one being the N7-guanosine adduct, followed by the N3-adenosine adduct (Segerback 1990). Substantial amounts of two adducts were observed that were suspected to represent the adenosine N7- and N1-adducts, and traces of *O*^6^-guanosine were detected (200-fold less than the amount of the N7 adduct). In vivo, the N7-guanosine hydroxyethyl adduct can be found in unexposed animals and human individuals and is ascribed to endogenous ethylene oxide formation. Interestingly, Wu et al. reported a much lower background level in rodent organs (0.2–0.3 µmol/mol guanosine in livers, lungs, brains and spleens of mice and rat, equivalent to 5–7.5 adducts per 10^8^ nt or 600–900 adducts per diploid cell) as compared to human peripheral blood lymphocytes (0.9–7.4 µmol/mol guanosine in 23 unexposed individuals, equivalent to 20–200 adducts per 10^8^ nt or 2500–20,000 adducts per diploid cell) (Wu et al. 1999b). In a follow-up study, a dose-dependent, yet non-linear adduct increase of 100–250-fold was observed in rats after 4 weeks of exposure to 100 ppm ethylene oxide for 6 h/day and 5 days a week (Wu et al. 1999a), with an over proportional adduct increase from 30 to 100 ppm in brains and lungs. In similar experiments, the steady-state level for the adenosine N7 adduct was reached within approximately 1 ½ weeks, indicating an adduct half-life of around 2 days (Walker et al. 2000).

Another valuable marker for ethylene oxide exposure is the adduct formed with the N-terminal valine of haemoglobin in rats, mice and men (Segerback 1990). This *N*^2^ adduct is formed with similar efficacy with haemoglobin from all three species, while other amino adducts differ substantially between species in their formation rate. Because its survival time is equivalent to that of haemoglobin itself and thus around 126 days in human (this value is the basis for the calculations cited below and is an accepted estimate, see Franco (2012)), the hydroxyethyl *N*^2^-valine adduct is a perfect integrating measure for average long-term ethylene oxide exposure. According to Boogaard (2002), an increment of 7 nmol hydroxyethyl valine per gram haemoglobin corresponds to an average continuous additional workplace exposure (40 h per week) of 1 ppm. Because this value was extrapolated from short term exposure to an 8 h per day exposure for 126 days, neglecting the exposure-free weekends, this value should actually be 5 nmol/g, based on its linear correlation with exposure duration during erythrocyte lifetime. Recent modelling approaches (Csanady et al. 2000; Filser and Klein 2018) predict the valine adduct burden of long-term exposure to 1 ppm ethylene oxide to be 2.5 nmol/g haemoglobin, in reasonable agreement with the above experimental values. Compared to the average background of 20 pmol hydroxyethyl valine per gram haemoglobin in non-exposed healthy non-smokers (Boogaard et al. 1999), such increase by almost three orders of magnitudes per ppm qualifies this lesion as a particularly sensitive marker of exposure.

##### Mutagenicity

Assessment of the mutagenicity of ethylene oxide in standard bacterial mutagenicity tests is not a straightforward approach, due to its volatility. However, substantial mutagenic activity has been reported by Hughes et al. (1987) using a modified procedure of the Ames test. On the other hand, the mutagenic potential of the ethylene oxide-induced DNA adducts in mammalian/human cells appears to be quite weak (Tompkins et al. 2009), which is in line with the observed low in vivo mutagenic potency in rodents (Recio et al. 2004; Tates et al. 1999).

##### Genotoxicity and carcinogenicity

Genotoxicity of ethylene oxide is well documented in vitro and in vivo (Appelgren et al. 1978; Farooqi et al. 1993; Tucker et al. 1986; Walker and Skopek 1993). Numerous epidemiologic studies clearly demonstrate that this is relevant for human workplace exposure. Enhanced micronuclei formation, sister chromatid exchanges and chromosomal aberrations have been frequently observed in studies with ethylene oxide workplace exposure levels above 0.5 ppm (Karelova et al. 1987; Richmond et al. 1985; Sarto et al. 1984). A non-linear dose–response relation has been observed in mice for heritable translocations beyond exposure concentrations of 100 ppm, yet this is paralleled by the non-linear ethylene oxide blood concentration in these animals, that is best explained by glutathione depletion (Brown et al. 1998; Generoso et al. 1990).

Carcinogenicity of ethylene oxide has been observed in rodents under conditions simulating workplace exposure at the concentration at or beyond 33 ppm. In rats, brain tumours and spleen mononuclear cell leukaemia were increased (Snellings et al. 1984) while mice showed a higher incidence of lung tumours (NTP 1987).

Two cohort studies on cancer mortality in ethylene oxide exposed workers are widely used to calculate carcinogenic risk in human: the Union Carbide Company (UCC) study (Teta et al. 1993) and the NIOSH cohort study (Stayner et al. 1993). The UCC study followed 1896 male workers exposed to ethylene oxide during chemical manufacturing with an average cumulative exposure level of 67 ppm-years. The NIOSH study (Stayner et al. 1993) observed 18,235 male (45%) and female (55%) sterilization workers that had a mean exposure level of 27 ppm-years, however, the median exposure in this study was only 5.6 ppm-years. Important (re-)analyses of the data of these studies have been performed by several authors (Kirman et al. 2004; Steenland et al. 2004; Valdez-Flores et al. 2010). The general conclusion of all of these studies is that there is little unequivocal evidence of increased incidence of malignancies due to the ethylene workplace exposure in these cohorts. Statistically significant (*p* < 0.05) is the increased incidence of bone tumours in the NIOSH cohort, that is, however, based on 6 observed against 2 expected tumours in the 18,000-person cohort. Despite a lack of statistical significance for its induction by ethylene oxide exposure, lymphoid malignancies were explored by Valdez-Flores and colleagues to estimate the tumour risk, taken as a worst-case scenario. They conclude that exposure to 0.25 ppm ethylene oxide during work time for 40 years leads at most to an increased incidence of 4 tumours in 100,000 workers (Valdez-Flores et al. 2010). This value is much lower than previous estimates by others (EPA 1985; OSHA 1984), yet is still a bit more conservative than a previous non-linear prediction model by the same group. The latter was based on mechanistic assumptions that are scientifically sound yet lack the proof of practical relevance (Kirman et al. 2004).

##### Conclusions

Despite a large pool of existing data, it is still very difficult to quantify the human risk for ethylene oxide exposure-related neoplasia at the workplace. The attempt by Valdez-Flores is an interesting approach but still falls short, as documented by the fact that the calculated risk of 4 in 100,000 would not cover the observed, statistically significant risk for bone malignancies, that, if the rather imprecise odds ratio of three would hold true, would come out to be approximately 8 in 100,000 at an exposure level of 0.25 ppm over the entire work life. However, taking the large human cohorts as the basis for the risk assessment appears to be the better approach than to take the animal experiments as the basis for risk calculation because the relevance of the latter for the human physiology is less evident. Because constant exposure to 0.25 ppm would increase the haemoglobin adduct approximately 50-fold over background, this biomarker appears to be perfectly suited to assess the actual burden of workers in an exposure range that should not yet present a significant health risk.

##### Open questions

One open question that has contributed to the uncertainty of ethylene oxide risk assessment is whether endogenous ethylene oxide production can be used as a starting point for setting limit values. This is based on the misleading statement that endogenous ethylene oxide production would result in an increase in the individual risk for malignancy by 1 in 10,000 (see above). This is definitely a heavy overestimation. If we extrapolate the endogenous formation rate from the haemoglobin adduct frequency in unexposed human individuals by linear regression we arrive at a workplace exposure equivalent of 0.003 ppm, which should represent a lifetime tumour risk increase of far less than 1 in 100,000. Therefore, endogenous formation rate is no relevant risk factor and should not be considered as a starting point for setting limit values.

An interesting open question that might support risk assessment addresses the observed tissue selectivity of ethylene oxide in different animal models. Why is the lung a particularly sensitive organ in the mouse while brain is more susceptible to ethylene oxide-induced tumorigenesis in rats? This is not necessarily expected because ethylene oxide does not require metabolic activation and should easily penetrate into all, even the bradytrophic tissues. One possible reason could be species differences in the tissue-specific glutathione depletion, which would probably be an irrelevant high dose effect. Another, more important mechanism could be cell-type specific efficacy of DNA repair mechanisms, such as *O*^6^-methyl guanosine methyltransferase (MGMT) prevalence.

#### Formaldehyde

##### General information

Formaldehyde exposure is attributed to exogenous sources (environment, indoor air, cosmetics, workplace) as well as to endogenous sources (metabolic intermediate). Due to the high chemical reactivity, formaldehyde causes local irritation and acute and chronic toxicity after direct contact in target tissues. Furthermore, formaldehyde is considered to be carcinogenic, inducing squamous cell carcinoma of the nose in experimental animals and with less compelling evidence nasopharyngeal carcinomas in humans. Also there is limited evidence for increased occurrence of leukaemia, nevertheless, without mechanistic or experimental support. On this database, formaldehyde was classified by IARC as a human carcinogen (Category 1). Considering the mode of action, the German MAK Commission classified formaldehyde in Category 4 for carcinogens, i.e. carcinogenic substances for which an increase in cancer risk is not expected provided that the MAK value is observed. The basis for this decision is, in spite of the induction of DNA lesions down to the low-dose levels, the assumption that an increase in mutagenicity and carcinogenicity is prevented as long as irritation and accelerated cell proliferation in the target cells are excluded. Therefore, a MAK value of 0.3 ppm (0.37 mg/m^3^) was established in 2000. Since then a number of publications have appeared that deal with the quantification of DNA lesions in the target tissues and the relationship of endogenously induced DNA damage compared to the same DNA lesions caused by exogenous exposure.

##### Comparison of endogenous formaldehyde formation with exogenous contribution from food sources

The European Food and Safety Authority (EFSA) compared the endogenous formaldehyde levels in humans with exogenous contribution from food sources. Formaldehyde is an intermediate generated within amino acid metabolism; an intracellular concentration of around 12 mg formaldehyde/L (400 μM) was estimated from measurements in nasal tissue (EFSA 2013). This leads to an estimated dose of approximately 0.61–0.91 mg/kg bw per minute or 878–1310 mg/kg bw per day, assuming a half-life of 1–1.5 min. Compared with formaldehyde turnover and the background levels of formaldehyde from food sources (1.7–1.4 mg/kg bw per day for a 60–70 kg person), including that from dietary methanol, the relative contribution of exogenous formaldehyde from consumption of animal products (milk, meat) from animals exposed to formaldehyde-treated feed was negligible (< 0.001%) (EFSA 2014b).

In addition, gene expression profiles were used to investigate whether a NOTEL (No Observed Transcriptional Effect Level) can be derived. The intent of the discussion in the joint working group of MAK and SKLM was to attain a risk assessment for the carcinogenicity of formaldehyde at the low dose level, taking into account most recent data.

##### Dose–response relationship of the carcinogenicity of formaldehyde

The Committee for Risk Assessment (RAC) of the European Chemicals Agency (ECHA) considered in 2012 all three industrial cohorts, comprising about 50 000 workers exposed to formaldehyde (Coggon et al. 2003; Hauptmann et al. 2004; Marsh and Youk 2005; Pinkerton et al. 2004). It has been concluded that the hypothesis of a causal association between formaldehyde exposure and mortality from nasopharyngeal cancer is supported only by evidence coming from the investigation of 4261 workers employed in plant 1 (Wallingford plant), one of the 10 plants investigated within the National Cancer Institute cohort (Hauptmann et al. 2004; Marsh and Youk 2005). It is, however, possible that this unique grouping of nasopharyngeal tumour cases in this one plant influencing the outcome of the entire NCI cohort could be caused by factors other than exposure to formaldehyde since three workers of the Wallingford plant had acquired nasopharyngeal tumours after a very short period of employment on a job with formaldehyde exposure. The meta-analysis (Bachand et al. 2010) on formaldehyde exposure and leukaemia demonstrates that there is little consistent evidence for a causal relationship and that the overall increased risk previously reported was driven by PMR (Proportionate Mortality Ratio) studies (ECHA 2012).

Regarding data from experimental animals, the information on nasal carcinogenesis in rats after inhalation (McGregor et al. 2010) showed that the tumour incidence was slightly elevated at an exposure level of 6 ppm formaldehyde and clearly elevated at 10 ppm and above, whereas at 0.7 and 2 ppm no additional nasal tumours occurred. Thus, there is a non-linear dose–response relationship. However, it must be considered that marginal increases in tumour rates cannot be detected with approximately 100 animals.

##### Gene polymorphism and gene knockout

No important gene polymorphism that caused increased susceptibility to formaldehyde as measured by DNA–protein cross-links and SCE-induction was identified in glutathione-dependent formaldehyde dehydrogenase (FDH), blood glutathione S-transferase (GST) M1 or GSTT1 of humans (Just et al. 2011; Zeller et al. 2011a, b, 2012).

In contrast, an association between genotoxicity biomarkers (CA, comet assay) and polymorphic genes coding for xenobiotic-metabolising or DNA repair enzymes was found in formaldehyde-exposed workers (84 anatomy pathology laboratory workers; 0.38 ppm formaldehyde ± 0.03 ppm) compared with controls. Regarding the effect of susceptibility biomarkers, results suggest that polymorphisms in *CYP2E1* and *GSTP1* involved in metabolism as well as *XRCC1* and *PARP1* involved in DNA repair pathways are associated with higher genetic damage in formaldehyde-exposed subjects (Costa et al. 2015).

Endogenous formaldehyde is removed by the enzyme alcohol dehydrogenase 5 (ADH5/GSNOR), and Adh5(-/-)-deficient mice accumulate formaldehyde-induced DNA adducts. Repair in liver, kidney and blood stem cells is mediated by FANCD2, a DNA cross-link repair protein. Adh5(-/-)Fancd2(-/-) mice lacking protection show greatly reduced bone marrow cellularity and complete failure of haematopoiesis within 3–7 weeks after birth. Increased levels of formaldehyde-induced DNA damage also cause karyomegaly and dysfunction of hepatocytes and nephrons. Bone marrow transplantation not only rescued haematopoiesis but, surprisingly, also preserved nephron function. Nevertheless, all of these animals eventually developed fatal malignancies. Formaldehyde, therefore, appears to be a relevant source of endogenous DNA damage that is counteracted in mammals by a conserved protection mechanism (Pontel et al. 2015).

##### Genotoxicity of formaldehyde

Formaldehyde induces DNA base damage, DNA–protein cross-links as well as DNA-DNA cross-links. DNA–protein cross-links and DNA-DNA cross-links occurred in rats starting from 0.3 ppm (0; 0.3; 0.7; 2; 6; 10 ppm ^14^C-formaldehyde, 6 h (Casanova et al. 1989)) and in monkeys starting from 0.7 ppm (0; 0.7; 2 or 6 ppm ^14^C-formaldehyde, 6 h (Casanova et al. 1991)) (reviewed in Greim 2002; McGregor et al. 2010).

**Fig. 7:**
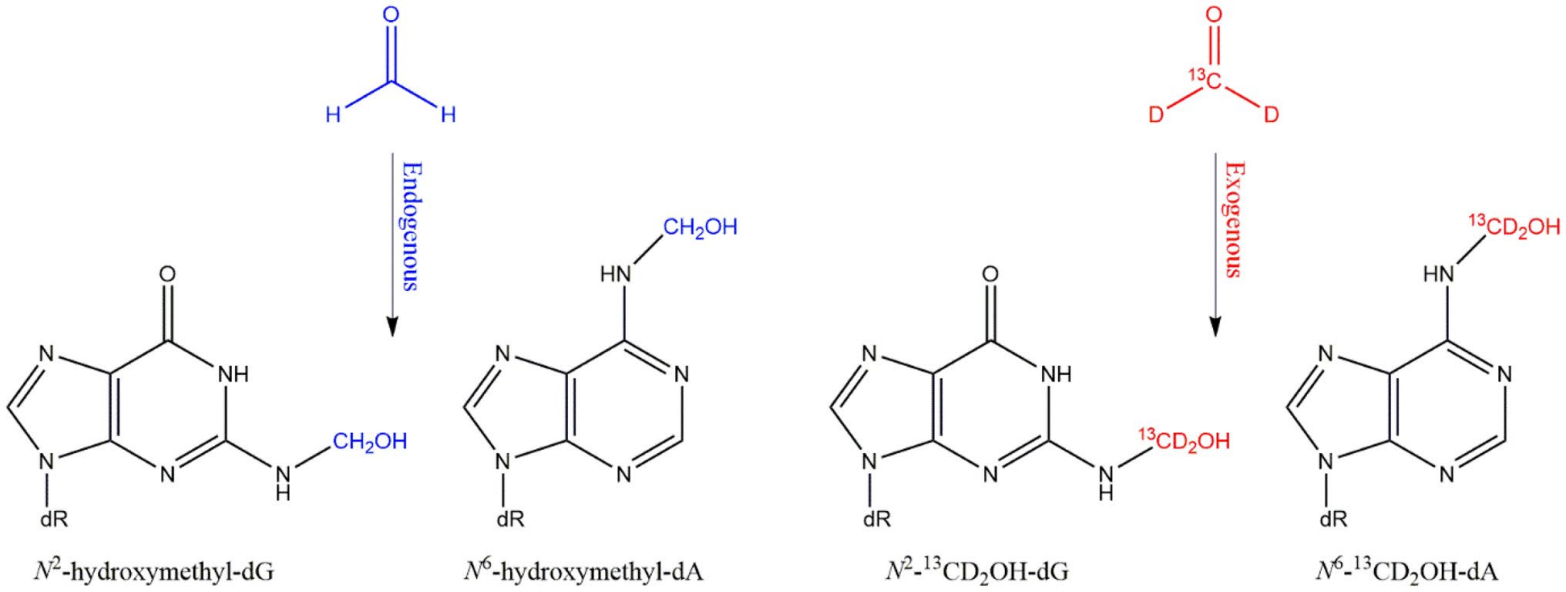
Formation of hydroxymethyl DNA adducts induced by formaldehyde (Swenberg et al. 2011)

Using stable isotopes (^13^CD_2_-formaldehyde, combined with MS analysis), Swenberg et al. (2011) were able to distinguish between endogenously and exogenously induced *N*^2^-hydroxymethyl-dG adducts (Fig. 7). The endogenous steady-state level of DNA base damage accounts for 47 ± 18 lesions/10^8^ dG (12 ± 4.5 lesions/10^8^ nucleotides) resulting from an endogenous formaldehyde burden of 100 µM per cell, measured in human blood and predominantly generated as an intermediate of the amino acid metabolism. In vivo formaldehyde exposure (6 h inhalation, rat) did not yield a threshold at which no additional base damage in the nose was detectable, but there was a sublinear dose–response relationship. Furthermore, the level of endogenous DNA lesions was exceeded only after exposure concentrations of 10 ppm and above. In other organs such as blood and bone marrow, no additional DNA base lesions were detected (Lu et al. 2010; Swenberg et al. 2011; Yu et al. 2015).

**Fig. 8:**
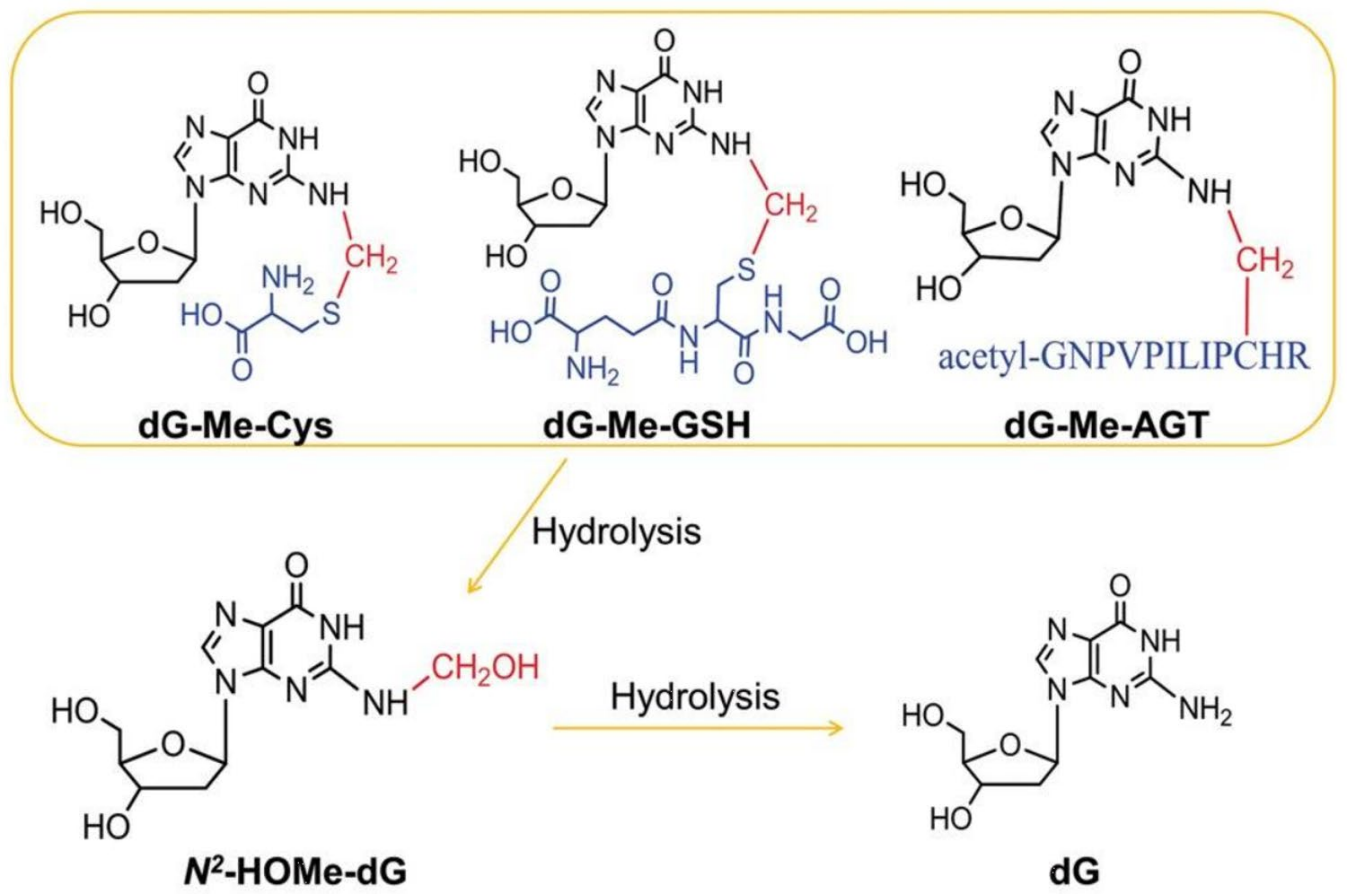
Hydrolysis of DNA–protein cross-links (Yu et al. 2015)

*N*^2^-Hydroxymethyl-dG DNA adducts (*N*^2^-HOMe-dG) were also shown to be the main hydrolysis product of several formaldehyde-induced DNA–protein cross-links investigated (Fig. 8). Both DNA lesions (*N*^2^-HOMe-dG and dG-Me-Cys) were examined with ultrasensitive nano-liquid chromatography–tandem mass spectrometry. DNA adducts resulting from exogenous formaldehyde inhalation were found in the nasal respiratory epithelium in the rat study (28-day, 2 ppm exposure) as well as in the monkey study (2-day, 6 ppm exposure), but not in any other tissue distant to the site of initial contact (Yu et al. 2015). Upon exposure of rats to lower concentrations of 0.001, 0.03, 0.3 ppm [^13^CD_2_]-formaldehyde for 28 days (6 h/day) by nose-only inhalation DNA adducts resulting from exogenous exposure were not detectable in any tissue sample, including the most susceptible nasal epithelium. Endogenous adducts were present in all analysed tissues (nasal epithelium, bone marrow, peripheral blood mononuclear cells (PBMCs), trachea, liver, hippocampus, olfactory bulbs, cerebellum and lung). Altogether, formaldehyde exposure at 0.001, 0.03 and 0.3 ppm did not alter the levels of endogenous formaldehyde induced DNA adducts or DNA–protein crosslinks (Leng et al. 2019). Therefore, the plausibility of the potential induction of leukaemia induced upon inhalation of formaldehyde must be seriously questioned.

Moreover, the amounts of exogenous formaldehyde-induced *N*^2^-HOMe-dG adducts were 3- to eightfold and 5- to 11-fold lower than the average levels of endogenous formaldehyde-induced *N*^2^-HOMe-dG adducts in rat and monkey nasal respiratory epithelium, respectively (inhalation of 2 or 6 ppm ^13^CD-formaldehyde, 6 h/d, 28 days). Thus, our advanced understanding of endogenous formaldehyde levels suggests that the risk of inhaled formaldehyde is apparently overestimated (Yu et al. 2015).

DNA–protein cross-link formation has been studied by an ultrasensitive and selective liquid chromatography-mass spectrometry method, where monkeys and rats were exposed to (^13^CD2)-labelled formaldehyde. This allowed the differentiation between DNA–protein cross-links from inhaled formaldehyde and DNA–protein cross-links from endogenous (naturally) formaldehyde. Monkeys were exposed at 6 ppm, 6 h per day for 2 days. Labelled DNA–protein cross-links were detected in the nasal tissue, but not in peripheral blood mononuclear cells, bone marrow or liver. In the nasal tissue, formation of endogenous DNA–protein cross-links was about three times higher than the exogenously generated ones. Endogenous DNA–protein cross-links were detected in all investigated tissues in varying amounts. Thus, endogenous DNA–protein cross-links were almost threefold higher in the liver than in the nasal tissue. When rats were exposed at 15 ppm, 6 h per day up to 4 days, exogenously generated DNA–protein cross-links were again only detected in the nasal tissue. Extension of exposure to 7 days (2 ppm, 6 h per day) resulted in a more pronounced formation of exogenous DNA–protein cross-links in nasal tissues with a proportion of nearly 50% relative to endogenous DNA–protein cross-links. In the post-exposure period, the amount of exogenously generated DNA–protein cross-links decreased by 20% within the first 24 h, followed by an even slower repair (Lai et al. 2016). It is noted that inhaled formaldehyde only caused DNA–protein cross-link formation in the nasal tissue; additional DNA–protein cross-link formation in internal organs was neither detected nor would it be plausible, due to the high reactivity of formaldehyde at the site of first contact (Nielsen et al. 2017). Rats were exposed by inhalation to either 0 or 2 ppm [^13^C^2^H_2_]-formaldehyde for 7 or 28 days, or 28 days followed by a 7-day recovery (6 h/day). Formation of exogenous N^6^-formyllysine protein adducts was observed in nasal epithelium and to some extent in trachea but not in distant tissues of lung, bone marrow, or white blood cells, with a twofold increase over endogenous N^6^-formyllysine over a 3-week exposure period. These results parallel the behaviour of DNA adducts and DNA–protein cross-links, with protein adducts cleared faster than DNA–protein cross-links, and point to the potential utility of N^6^-formyllysine protein adducts as biomarkers of formaldehyde (Edrissi et al. 2017).

##### Repair of formaldehyde-induced DNA lesions

Repair of the formaldehyde-induced intra-strand cross-links is mediated by the nucleotide excision repair (NER) pathway (Kawanishi et al. 2014). Formaldehyde-induced DNA–protein cross-links may be repaired by the NER repair combined with homologous recombination (HR) (de Graaf et al. 2009; Kawanishi et al. 2014; McHale et al. 2014). Furthermore, DNA–protein cross-links may partly be broken down by specific proteolytic enzymes, allowing translesion synthesis polymerases (a potentially mutagenic pathway) to replicate across DNA-peptide lesions. Additionally, a tolerance pathway also exists, allowing replication across unrepaired DNA–protein cross-links lesions that may generate DNA strand breaks as intermediates (potentially causing genomic rearrangements) followed by strand ligation (Stingele et al. 2015). Not least, the Fanconi anaemia pathway is important in the repair of inter-strand DNA cross-links and DNA–protein cross-links (Kirsch-Volders et al. 2014; McHale et al. 2014; Ren et al. 2013; Schneider et al. 2015).

##### Risk assessment for carcinogenicity at the low dose level on the basis of induction of DNA base damage

Swenberg et al. (2011) have undertaken a tumour risk assessment for formaldehyde under different “worst-case” assumptions. Assuming that (1) all nasal tumours are induced by formaldehyde exposure and (2) a linear increase of the tumour risk is accompanied by an increased number of DNA-dG adducts, the authors estimated a nearly tenfold lower tumour risk for nasal tumours than was derived by the US EPA (US Environmental Protection Agency) from the human formaldehyde-based carcinogenesis data after conservative linear extrapolation from the high dose level.

The new molecular dosimetry information for formaldehyde-induced DNA adducts in the bottom-up approach was used to estimate upper-bound lifetime human nasopharyngeal cancer and leukaemia risks that might arise from continuous inhalation exposure to 1 ppm formaldehyde. Comparison of the resulting bottom-up risk estimates with corresponding top-down estimates derived by US EPA from epidemiological data for exposed workers shows the latter to be markedly higher (Starr and Swenberg 2013). Updated estimates of added risk for leukaemia and nasopharyngeal cancer rely on robust estimates of tissue-specific endogenous and exogenous formaldehyde-induced DNA adducts in monkeys and of DNA-adduct elimination half-life in rats. The risk to humans from inhaled formaldehyde may have been overestimated with the top-down approach and the role of endogenous formaldehyde in the development and progression of bone marrow toxicity and leukaemia may have been underappreciated. It is, therefore, important to consider endogenous sources of DNA–protein cross-links and DNA mono-adducts with the bottom-up approach where there is little or no empirical evidence of a positive dose–response at low exogenous exposure levels. This approach can be used as a useful “reality check” during the risk assessment process (Andersen et al. 2019; Farland et al. 2019; Starr and Swenberg 2016; Yu et al. 2015).

Uptake of formaldehyde in the nose of rats, monkeys and humans was estimated by means of an anatomically accurate computational fluid dynamics model, regarding net overall absorption and potential exhalation of formaldehyde on nasal airway walls. At ≥ 1 ppm, the predicted nasal uptake was about 99, 87 and 85% for rats, monkeys and humans, respectively. At lower concentrations the predicted uptake was nonlinear. At an exposure concentration of 1 ppb, predicted nasal uptake was 17.5 and 42.8% in the rat and monkey model. However, based on this model, in case of humans, the exhalation would even exceed the inhalation, which does not appear to be plausible. Higher fluxes were predicted to occur in regions located in the more anterior sections of the nose (Schroeter et al. 2014).

##### DNA damage and mutations in target cells

An important question concerns the target cells of formaldehyde-induced carcinogenesis. The relevant target cells for nasal tumours are the basal cells, whereas the development of DNA damage is usually quantified in the entire nasal mucosa. Formaldehyde is a highly reactive substance that undergoes rapid metabolism and may not reach the target cells of nasal carcinogenesis at the low dose level. Specific studies addressing the induction of any type of DNA damage, including DNA–protein cross-links and DNA–DNA cross-links in basal cells are missing (Speit et al. 2011). Therefore, the biological relevance of the induced DNA damage and their potential conversion into mutations would depend on whether the relevant target cells (basal cells) are reached and to what extent these target cells are stimulated to proliferation. Also data on the induction of micronuclei can be used to support this theory. In studies with volunteers exposed to 0.7 ppm formaldehyde in the inhaled air (4 h/day, 5 days with 0.3–0.4 ppm and 4 peaks of 0.6–0.8 ppm for 15 min), an increase in the incidence of micronuclei was not detected in exfoliated nasal mucosa cells (Zeller et al. 2011a).

##### Impact of formaldehyde on transcription and gene expression profiles

One important aspect in formaldehyde-induced carcinogenicity consists in its impact on cell proliferation, which in rats is elevated at 6 ppm and above (McGregor et al. 2010; Speit et al. 2011). This raises the question whether transcriptional changes in the expression of genes related to cell growth may be used as sensitive markers, at the mRNA-level, which may serve for the derivation of a NOTEL for cell proliferation at the target cells (Andersen et al. 2010; Zarbl et al. 2010). Rats were exposed to 0 (control), 0.7, 2, 6, 10 and 15 ppm formaldehyde 6 h/day for 1, 4 or 13 weeks. Nasal tissue concentrations of formaldehyde acetal (CH_2_(OH)_2_), GSH, GS-formaldehyde and DNA–protein cross-links were analysed by means of a pharmacokinetic model, taking into account the background formaldehyde acetal and GSH levels. For DNA–protein cross-links no background levels were predicted reproducing the observed data of Casanova et al. (1994). The cellular levels of formaldehyde acetal and DNA–protein cross-links only showed minor increases at exposures at 0.7 and 2 ppm formaldehyde. At these levels, GSH decreased slightly; above 4 ppm, the changes were more pronounced. Histopathology showed nasal lesions at 2 ppm and epithelial cell proliferation at higher concentrations. The lowest transcriptional benchmark dose (BMD) for significant changes in gene expression (sensitive response genes) was approximately 1 ppm in comparison to the transcriptional BMDs for significant changes in the cell cycle (3 ppm) and DNA damage pathways (4 ppm) after 13 weeks of exposure. At all exposure times at 6 ppm and above the expression of sensitive response genes was elevated and reached a plateau at 10 ppm. The authors concluded that formaldehyde levels below 1 or 2 ppm would not affect gene expression above effect potentially evoked by endogenous formaldehyde levels within the epithelial cells (Andersen et al. 2010, 2008).

In addition to DNA damage and gene expression, formaldehyde exerts also epigenetic effects leading to the modification of histone H3 (Yoshida and Ibuki 2014), as well as altered patterns of miRNA (Chappell et al. 2016; Chen et al. 2017; Rager et al. 2011, 2013, 2014).

Non-human primates (cynomolgus macaques) were exposed for 6 h/day for 2 days to 0, 2, or 6 ppm formaldehyde and the miRNA expression profile of nasal tissue from the maxilloturbinate region was analysed. Based on the miRNA microarray analysis, the expression of four most altered miRNAs was confirmed by RT-PCR. In this analysis, exposure to 2 ppm formaldehyde caused significantly increased expression of miR-125b and decreased expression of miR-145. In response to 6 ppm formaldehyde miR-125b and miR-152 were significantly increased in expression whereas miR-145 and miR-142-3p were significantly decreased. The evaluation of predicted targets of miR-125b revealed decreased expression of genes involved in apoptosis signalling (Rager et al. 2013). Rats were exposed by inhalation to either 0 or 2 ppm formaldehyde for 7 or 28 days, or 28 days followed by a 7-day recovery (6 h/day). miRNAs showed altered expression in the nose (84, 59, 0 number of formaldehyde-responsive miRNAs, increased or decreased, in the three exposure groups) and circulating white blood cells (31, 8, 3) but not in the bone marrow. In the same study applying the same exposure scenario microarray analysis was performed to measure transcript levels. In nasal tissue expression levels of 830 mRNA transcripts was altered after 7 days compared to 42 transcripts after 28 days of exposure. In white blood cells, the distribution was 96 and 130 altered mRNA transcripts, respectively. In the nose, the decreased expression of the let-7 family of miRNAs implicated a reduced activity of these tumour suppressors that act on apoptosis/cell proliferation pathways. Beside this, enrichment analysis of miRNAs and mRNAs pointed to alteration in the inflammation and immune system in the examined tissues (Rager et al. 2014).

##### Summary and perspective

Formaldehyde induces nasal tumours with a sublinear dose–response relationship. While IARC–based on hazard–assigned it to Group 1 (carcinogenic to humans), the German MAK Commission classified it in group 4, defining a MAK value which protects from carcinogenicity. The latter approach has been supported by recent developments in the quantification of both endogenous and exogenously induced DNA adducts. Thus, carcinogenicity is due to the induction of different types of DNA lesions as well as to an increase in cell proliferation. DNA damage is generated already at the low dose level. However, after inhalation, levels of DNA damage in internal organs indicative for systemic toxicity, in a similar magnitude as levels derived from the endogenous formaldehyde burden were not reached until about 100 ppm. With respect to the nose as the critical target organ for nasal tumours, it is unclear whether inhaled formaldehyde at the low dose levels reaches the basal cells as target cells at all or whether it undergoes rapid metabolism and/or reaction in the upper cell layers. To clarify this aspect, further research is required. In addition to DNA damage, accelerated cell proliferation is needed for relevant conversion of DNA lesions into mutations. In this context, data from animal experiments analysing gene expression profiles revealed a transcriptional BMD at the mRNA level of 1 ppm for significant changes in sensitive response genes associated with cellular stress, inflammation, and cell proliferation. At miRNA level, 2 ppm induced transcriptional responses such as immune system/inflammation and apoptosis/proliferation. These data are in agreement with the irritation of the eye or nose and throat in human volunteers observed at 0.5 or 1 ppm. Therefore, considering the currently available database it can be assumed that below the level of irritation, there is no relevant additional risk of nasal tumours at the low dose level, such as exposure at the MAK value of 0.3 ppm (0.37 mg/m^3^). Also, mechanistic and experimental data appear to exclude an elevated risk of internal organs, including lymphoma. This is supported by the bottom-up approach assessing the extent of DNA lesions due to endogenous and exogenous formaldehyde exposure, providing convincing evidence that added cancer risk from exposure to all airborne formaldehyde concentrations is far lower than risks estimated by linear extrapolation from high exposure levels. This approach can be used as a useful “reality check” during the risk assessment process where there is little or no empirical evidence of a positive dose–response at low exogenous exposure levels.

### Absence of endogenous background levels of the same or similar DNA lesions

#### Aflatoxin B_1_

##### Introduction

**Fig. 9:**
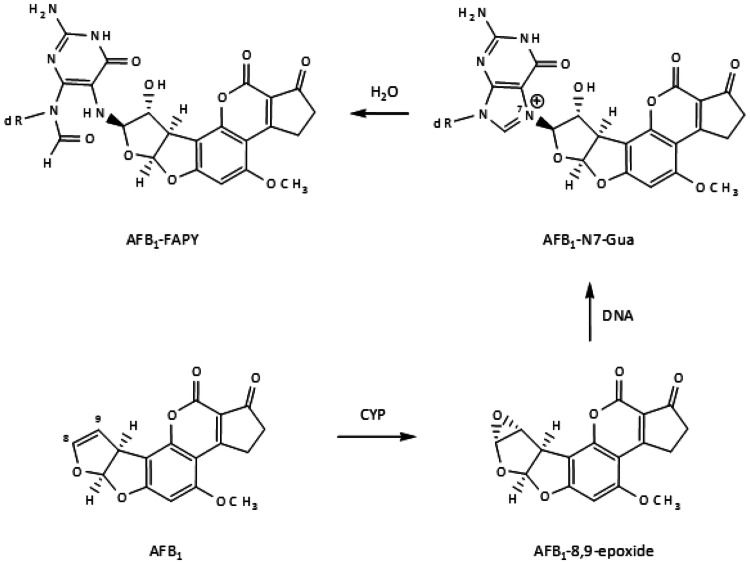
Metabolic activation of aflatoxin B_1_ (AFB_1_) to AFB_1_-8,9-epoxide and subsequent formation of the primary AFB_1_-DNA adduct 8,9-dihydro-8-(N7-guanyl)-9-hydroxyaflatoxin B_1_ (AFB_1_-N7-Gua), which can give rise to two secondary lesions, an apurinic site or the ring-opened AFB_1_-formamidopyrimidine (FAPY) adduct. CYP, cytochrome P450; dR, deoxyribose

Aflatoxin B_1_ (AFB_1_) is a mycotoxin produced, among other fungi, by *Aspergillus flavus* and contaminates a variety of food items including groundnuts, tree nuts, dried fruit, spices and figs. Human exposure to the mycotoxin is particularly high in those regions of the world in which the above-mentioned foods are improperly stored. Moreover, in these regions a high prevalence of hepatitis B virus infections is observed, and epidemiological studies support the view that AFB_1_ and hepatitis B virus infections synergistically interact to induce hepatocellular carcinomas in humans. AFB_1_ is per se biologically inactive, but following ingestion can be metabolically activated by cytochromes P450 1A2 and 3A4 to AFB_1_-8,9-epoxide (Fig. 9) in rats and humans (Busby and Wogan 1984; Croy et al. 1978; Croy and Wogan 1981a; Croy and Wogan 1981b; Groopman et al. 1981; Lin et al. 1977; Martin and Garner 1977). The epoxide may react with DNA to form the primary AFB_1_-DNA adduct 8,9-dihydro-8-(N7-guanyl)-9-hydroxyaflatoxin B_1_ (AFB_1_-N7-Gua), which in turn can give rise to two secondary lesions, an apurinic site or the AFB_1_-formamidopyrimidine (AFB_1_-FAPY) adduct (Fig. 9). The highly persistent AFB_1_-FAPY adduct that consists of an equilibrium mixture of two presumably rotameric forms is thought to be responsible for the strong genotoxicity and mutagenicity of the mycotoxin that ultimately may lead to liver cancer (Smela et al. 2002).

##### AFB_1_-mediated liver tumour induction in rats and rainbow trout

Some strains of rats (e.g. the Fischer 344 strain) are particularly sensitive regarding liver tumour induction by AFB_1_. In this context, a rather limited number of studies, in which AFB_1_ was administered to rats p.o. and in which carcinogenicity data are available, have been published (Butler and Barnes 1968; Elashoff et al. 1987; Epstein et al. 1969; Newberne and Rogers 1973; Nixon et al. 1974; Wogan and Newberne 1967; Wogan et al. 1974) and are summarized in Table 4. Taken together, these studies demonstrate that AFB_1_ given orally induces liver tumours in rats in a dose-dependent manner and that liver tumour induction definitely also occurs at concentrations well below 1 µg/kg bw/day, the only exception being a study by Elashoff et al. (1987), in which doses of 0.25 and 0.75 µg AFB_1_/kg bw/day did not lead to liver tumours in male and female Fischer 344 rats. However, as discussed by Benford et al. (Benford et al. 2010), most of these studies are actually not suited for dose–response modelling when wanting to assess the risk derived from the presence of AFB_1_ in food, since AFB_1_ was administered in a single dose or produced a 100% response in all treated animal groups. One study that was used for the calculation of margin of exposure values for AFB_1_ (Benford et al. 2010) and in which human-relevant dietary AFB_1_ levels were tested was reported by Wogan et al. (1974). Male Fischer 344 rats were fed 1, 5, 15, 50 or 100 ppb AFB_1_ (corresponding to a dose of 0.04, 0.2, 0.6, 2 and 4 µg AFB_1_/kg bw/day, respectively) for up to 104 weeks and a dose-dependent liver tumour incidence was observed (Table 4), even the lowest dose used (0.04 µg/kg bw/day) being able to induce liver tumours in 2 out of 22 animals.

**Table 4:** Induction of liver tumours in rats after dietary administration of aflatoxin B_1_ ( reproduced from EFSA 2007)

Rat strain/Sex	Dose (µg/kg bw/day)	Duration of dosing	Tumour incidence	Comments	References no
Fischer 344/ male	0	80 w	0/25		Wogan and Newberne (1967)
	0.75	68 w	12/12		
	15	35–52 w	6/20		
	50	35–41 w	18/22		
	50	2 w	1/16	after 82 w	
Fischer 344/ female	0	80 w	0		Wogan and Newberne (1967)
	0.75	68 w	13/13		
	15	60–70 w	11/11		
	50	64 w	4/4		
	50	2 w	1/13	after 82 w	
Porton/female	0	104 w	0/34		Butler and Barnes (1968)
	5	104 w	5/30		
	25	104 w	26/33		
Porton/male	0	104 w	0/46		Butler and Barnes (1968)
	4	104 w	17/34		
	25	104 w	25/25		
Wistar/male	0	147 d	0/24		Epstein et al. (1969)
	12.5	147 d	8/13	after 742 d	
	25	147 d	13/18	after 622 d	
	50	147 d	12/14	after 611 d	
CDR/male	0	104 w	0/50		Newberne and Rogers (1973)
	4	104 w	24/50		
Fischer/female	0	104 w	0/15		Nixon et al. (1974)
	1	104 w	1/15		
Fischer/male	0	104 w	0/16		Nixon et al. (1974)
	0.8	104 w	5/13		
Fischer 344/male	0		0/18		Wogan et al. (1974)
	0.04	104 w	2/22		
	0.2	93 w	1/22		
	0.6	96 w	4/21		
	2	82 w	20/25		
	4	54 w	28/28		
Fischer 344/female	0	104 w	0/144		Elashoff et al. (1987)
	0.25	104 w	0/24		
	0.75	104 w	0/24		
	2.25	104 w	1/24		
Fischer 344/male	0		1/144		Elashoff et al. (1987)
	0.25		0/23		
	0.75		0/24		
	2.25		1/23		

In contrast to rats, mice show a remarkable resistance towards AFB_1_. This is due to the fact that mice constitutively express glutathione *S*-transferase Yc, which is extremely efficient in detoxifying the AFB_1_-8,9-epoxide (Buetler and Eaton 1992; Buetler et al. 1992; Eaton and Gallagher 1994; Hayes et al. 1991; Ramsdell and Eaton 1990).

In rainbow trout (*Oncorhynchus mykiss*) the activation of AFB_1_ to AFB_1_-8,9-epoxide is mainly catalysed by CYP 2K1 (Williams and Buhler 1983) and, as in mammals, AFB_1_-N7-Gua is formed and spontaneously converted to AFB_1_-FAPY (Bailey et al. 1988; Croy et al. 1980). Furthermore, AFB_1_ is able to induce time- and dose-dependently liver tumours in rainbow trout (Bailey et al. 1996b). This has been documented in at least seven studies (Ayres et al. 1971; Bailey et al. 1987, 1994, 1998; Dashwood et al. 1989; Lee et al. 1968, 1971) and Table 5 lists the data of five of them. In the other two studies (Bailey et al. 1998; Dashwood et al. 1989) a dose-dependent induction of liver tumours in rainbow trout was also shown, but the tumour incidence data were only documented in form of figures so that they could not be listed. Finally, more recently it has been documented that AFB_1_ also elicits a linear dose–response curve when administered at ultra-low doses (Williams 2012; Williams et al. 2009). Although the latter study only reported preliminary results, which are in the process of being published, these demonstrate that the AFB_1_ dose-tumour response is linear even at ultra-low doses. All in all, the studies clearly indicate that, as in the case of rats, there is no “threshold” for the hepatocarcinogenic effect of AFB_1_ in rainbow trout.

**Table 5:** Induction of liver tumours in rainbow trout after dietary administration of aflatoxin B_1_

AFB_1_ level (ppb)	Duration of dosing	Tumour incidence	Comments	References
0	12 mo	0/50		Croy et al. (1980)
4	12 mo	10/20		
0	15 mo	0/50		Croy et al. (1980)
0.4	15 mo	15/106		
4	15 mo	65/108		
0	12 mo	0/20		Bailey et al. (1988)
4	12 mo	10/40		
8	12 mo	40/57		
20	12 mo	62/80		
0	16 mo	0/40		Bailey et al. (1988)
4	16 mo	14/40		
8	16 mo	32/57		
20	1 d	2/59	sacrificed 12 mo after AFB_1_ exposure	Bailey et al. (1996b)
	5 d	6/51		
	10 d	5/50		
	20 d	23/57		
	30 d	17/47		
0	4 w	0/49	sacrificed 11 mo after AFB_1_ exposure	Lee et al. (1968)
20	4 w	5/49		
0	2 w	0/192	sacrificed 9 mo after onset of AFB_1_ exposure	Lee et al. (1971)
4	2 w	25/382		
8	2 w	98/387		
16	2 w	194/389		
32	2 w	287/389		
64	2 w	302/383		

##### AFB_1_-DNA adduct formation in the liver of rats and rainbow trout

In a study by Lutz (1987) tritiated AFB_1_ was administered p.o. to Fischer 344 rats on 10 consecutive days, liver DNA was isolated 24 h after the last dose and AFB_1_-DNA binding was determined. A linear dose–response relationship was found over four orders of magnitude and particularly in the low dose range, while in the high dose range a flattening out of the curve was seen, probably due to saturation of the activating enzyme systems (Lutz 1987). The lowest dose used in this study was 1 ng AFB_1_/kg bw, which corresponds to about 60 ng per man, i.e. a dose that is taken up daily in certain areas of Africa and Asia. In a later study by the above-mentioned research group (Buss et al. 1990) tritiated AFB_1_ was administered p.o. to Fischer 344 rats at three exposure levels for 4, 6 or 8 weeks, liver DNA was isolated and AFB_1_-DNA binding was determined. After 4 weeks the AFB_1_-DNA adduct level did not increase significantly, thereby indicating that a steady-state for adduct formation and removal had nearly been reached, whereas after 8 weeks the AFB_1_-DNA adduct level was proportional to the dose (Buss et al. 1990). In a study by Cupid et al. (2004) the levels of AFB_1_-DNA adducts were quantified by accelerated mass spectrometry following the oral administration of very low doses (i.e. dietary levels of exposure) of AFB_1_ (0.16 ng/kg bw-12.3 µg/kg bw). It could be shown that AFB_1_-DNA adduct formation was linear over the whole dose range (Cupid et al. 2004).

A dose-dependent linear increase in the number of liver AFB_1_-DNA adducts was also observed after feeding rainbow trout with human-relevant concentrations of AFB_1_ over a period of one to four weeks (Bailey et al. 1998, 1994; Dashwood et al. 1988, 1989). The rate of repair of AFB_1_-DNA adducts in trout is much slower than in mammals: the “half-life” of the AFB_1_-N7-Gua adduct is 7.5 h in rats, while it is about 21 days in trout (Bailey et al. 1988). As noted by Bailey et al. (1996b) the loss of AFB_1_-N7-Gua adducts can be due to chemical conversion, depurination or enzymatic removal and is not a first-order process, so that the above-mentioned values do not reflect true half-lives. However, the long “pseudo half-life” of the AFB_1_-N7-Gua adduct (21 days) in rainbow trout could largely be due to the decreased ability of this animal species to repair bulky DNA adducts.

AFB_1_-DNA adduct levels show a high correlation with tumour incidence in rats as well as rainbow trout (Bailey et al. 1998; Bechtel 1989; Dashwood et al. 1989; Otteneder and Lutz 1999). Based on these results it has been postulated that the adducts persisting in the liver of treated animals induce the formation of hepatic tumours with a similar efficiency in both animal species (Bailey 1994; Bailey et al. 1994; Dashwood et al. 1990; Dashwood et al. 1992). Moreover, Otteneder and Lutz (1999) analysed the DNA adduct levels after the repeated administration of 27 carcinogens, including AFB_1_, to rats or mice and, to correlate them with tumour incidence, the DNA adduct levels measured at a given dose were normalized to the dose that results in a 50% tumour incidence under the conditions of a 2-year bioassay (TD_50_). The lowest calculated adduct level leading to a 50% liver tumour incidence in rat liver (“adduct level at TD_50_”) was that corresponding to AFB_1_ (Otteneder and Lutz 1999). In this context, the fact that the adduct level at TD_50_ for AFB_1_ was 53 per 10^8^ nucleotides and 2082 per 10^8^ nucleotides for dimethylnitrosamine was interpreted by Otteneder and Lutz (1999) to mean that the AFB_1_-DNA adducts are about 40 times more potent in inducing liver tumours than the DNA adducts formed by dimethylnitrosamine. Boysen et al. (2009) reviewed the scientific literature to evaluate the mechanistic evidence for the involvement of N7-guanine adducts formed by nitrosourea compounds, nitrosamines, hydrazines and olefins in mutagenesis and concluded that there is little to no evidence that N7-guanine adducts or their depurination product, the apurinic sites, are the cause of mutations in cells and tissues. In contrast, the ring-opened lesions derived from N7-guanine adducts are much more persistent and have a higher mutagenic potency. This also applies to AFB_1_: it has been shown that the mutation frequency of AFB_1_-N7-Gua is much lower (about sixfold) than that of AFB_1_-FAPY (Bailey et al. 1996a; Smela et al. 2002).

While no study relating cytotoxicity or cell proliferation rate to AFB_1_-mediated liver tumour formation in rats has been published up to now, a report by Nunez et al. (1990) analysed the relationship between AFB_1_ metabolism and cytotoxicity in rainbow trout treated with 0.05, 0.1, 0.25 or 0.5 mg/l water for 30 min and sacrificed 1 or 2 weeks later. After two weeks 4 out of 11 animals exposed to 0.25 mg/l water exhibited severe hepatic cytotoxicity, while all 8 animals exposed to 0.5 mg/l water showed a severe cytotoxic response (Nunez et al. 1990). In contrast, fish exposed to 0.05 or 0.1 mg/l water did not show any signs of cytotoxicity. Hence, AFB_1_-mediated liver cytotoxicity in rainbow trout seems not to display a linear correlation with the administered amount of the mycotoxin. Moreover, it was shown that cytotoxicity is dependent on the activation of AFB_1_ to AFB_1_-8,9-epoxide (Nunez et al. 1990). The critical macromolecular targets leading to cytotoxicity were not named in that study (Nunez et al. 1990), but in a study by Jennings et al. (1994), in which freshly isolated rat hepatocytes were incubated with increasing concentrations of AFB_1_, it could be shown that cytotoxicity correlated with a loss of glutathione *S*-transferase activity. Up to now, a study correlating cytotoxicity with AFB_1_-mediated hepatocarcinogenesis during the whole (i.e. at early and late stages of the) hepatocarcinogenic process in a mammal or non-mammal animal species is missing.

##### ***p53*** mutations, hepadnavirus infections, and AFB_1_-mediated liver cancer formation

A number of studies have linked the formation of hepatocellular carcinomas in humans to a specific mutation in the 3^rd^ base of codon 249 of the *p53* gene induced by the dietary intake of AFB_1_ (reviewed for example in Hussain et al. (2007)). The codon 249 *p53* mutation has been detected in up to 50% of hepatocellular carcinoma cases from regions highly exposed to AFB_1_ such as Qidong and Tongan in China, India, Southern Africa, The Gambia, Mozambique and Senegal (Katiyar et al. 2000; Kirk et al. 2000; Rashid et al. 1999; Shimizu et al. 1999; Yang et al. 1997). In regions where exposure to AFB_1_ is moderate, e.g. Beijing, Shanghai, Xi’an, Hong Kong, Singapore, South Korea, Taiwan, Thailand, Vietnam, South Africa and Egypt, about 7–34% of the hepatocellular cases examined had mutations at the 3rd position of codon 249 of the *p53* gene (Chittmittrapap et al. 2013; Lunn et al. 1999, 1997; Qi et al. 2015; Shen and Ong 1996; Thongbai et al. 2013; Yang et al. 1997), whereas in regions with a low exposure to AFB_1_ such as Australia, Europe, Japan and USA only 1% of the hepatocellular carcinoma samples analysed had the above-mentioned mutation (Boix-Ferrero et al. 1999; Hussain et al. 2007; Rashid et al. 1999; Vautier et al. 1999).

Aguilar et al. (1994) examined normal liver samples from the United States, Thailand, and Qidong, i.e. from regions in which AFB_1_ exposure is negligible, low and high, respectively, for *p53* gene mutations. The frequency of the AGG to AGT mutation at codon 249 of the *p53* gene paralleled the level of AFB_1_ exposure, which supports the hypothesis that this toxin plays a causative and early role in liver cancer development.

In those regions, in which AFB1 contamination of the food chain as well as the hepatocellular carcinoma incidence are high, as in Africa and China (see above), chronic viral hepatitis is widespread. In this context a number of studies have shown that the relative risk to develop liver cancer is significantly higher in the case of a concomitant high dietary AFB_1_ exposure and a chronic hepatitis B virus (HBV) infection if compared to a high dietary AFB_1_ exposure or a chronic HBV infection alone, i.e. both factors act synergistically in hepatocarcinogenesis (Table 6) (Kew 2003; Lunn et al. 1997; Qian et al. 1994; Ross et al. 1992; Wang et al. 1996). As to the possible mechanisms underlying the interaction between AFB_1_ and HBV in liver carcinogenesis, a number of hypotheses have been postulated, among others the following: (1) enhanced hepatocyte necrosis and proliferation caused by chronic HBV infection may increase the probability that AFB_1_-induced mutations are generated and subsequently may lead to the clonal expansion of cells containing these mutations (Chisari et al. 1989); (2) a chronic necroinflammatory disease resulting from HBV infection results in the generation of oxygen and nitrogen reactive species, which in turn may induce gene mutations in liver cells (Liu et al. 1994; Ohshima and Bartsch 1994); (3) the HBx protein, an HBV protein that interferes with the nucleotide excision repair pathway (Jia et al. 1999), thereby favouring the persistence of AFB_1_-DNA adducts and the induction of gene mutations. Studies in The Gambia (Gouas et al. 2012) and Thailand (Ortiz-Cuaran et al. 2013) showed that complete HBx sequences are often associated with the presence of the codon 249 *p53* gene mutation. Although the combined high dietary AFB_1_ exposure and HBV infection strongly increase the risk of developing liver cancer, HBV infection seems not to be required to induce the codon 249 *p53* gene mutation (Hsu et al. 1993; Stern et al. 2001).

**Table 6:** Relative risk to develop liver cancer due to a chronic viral hepatitis B virus (HBV) infection alone, due to a dietary aflatoxin B1 (AFB1) exposure alone and due to a concomitant chronic HBV infection and a high dietary AFB1 exposure (HBV + AFB1) (reproduced from Kew 2003)

Reference	HBV alone RR (95% CL)	AFB_1_ alone RR (95% CL)	HBV + AFB_1_ RR (95% CL)
Ross et al. (1992)	4.8 (1.2–19.7)	1.9 (0.5–7.5)	60.1 (6.4–561.8)
Qian et al. (1994)	7.3 (2.2–24.4)	3.4 (1.1–10.0)	59.4 (15.6–212)
Wang et al. (1996)	17.4 (3.6–143.4)	0.3 (0–3.6)	70.0 (11.5–425.4)
Lunn et al. (1997)	17.0 (2.8–103.9)	17.4 (3.4–90.3)	67.6 (12.2–373.2)

*RR* relative risk, *CL* confidence limits

In contrast to the situation in humans, *p53* mutations have definitely not been detected in AFB_1_-induced liver tumours in rats (Hulla et al. 1993; Lee et al. 1998; Liu et al. 1996; Tokusashi et al. 1994), and in rainbow trout this aspect has not been investigated up to the present time. One could argue that in rats the site corresponding to codon 249 of the human *p53* gene is CGG instead of AGG (Hulla et al. 1993) and that a change in the 3rd base would produce a silent mutation. However, AFB_1_ binding to the second base of this codon could produce a change from arginine to leucine, and this effect has never been reported for AFB_1_-induced rat liver tumours (Hulla et al. 1993; Lee et al. 1998; Liu et al. 1996; Tokusashi et al. 1994). Hence, at least in the rat *p53* mutations cannot be used at all to follow up the induction of liver tumours by AFB_1_ in the low (or even the high) dose range.

##### Glutathione ***S***-transferase and DNA repair enzyme polymorphisms and AFB_1_-mediated liver cancer formation

The highly reactive AFB_1_-8,9-epoxide can efficiently be detoxified by conjugation with glutathione in a glutathione *S*-transferase (GST)-catalysed reaction, thereby protecting cellular DNA by preventing AFB_1_-DNA adduct formation (Guengerich et al. 1998; Johnson et al. 1997). Therefore, genetic polymorphisms affecting the expression of these enzymes may alter the mutagenicity and carcinogenicity of AFB_1_ at a given exposure level. Two GST isoenzymes, GSTM1 and GSTT1, are able to conjugate the AFB_1_-8,9-epoxide (Guengerich et al. 1998; Johnson et al. 1997) and exhibit a deletion polymorphism, which leads to a complete loss of expression of the GSTs at the protein level in individuals homozygous for the deletion. Whereas many studies could not demonstrate that the GSTM1 or GSTT1 null genotype was associated with an increased hepatocellular carcinoma risk, a number of studies reported an enhanced liver cancer risk associated with a high aflatoxin exposure in individuals with the GSTM1 or GSTT1 null genotype as well as with the double null genotype (Chen et al. 1996, 2012, 2014; Kirk et al. 2005; Liu et al. 2013; Long et al. 2006; Shen et al. 2014; Song et al. 2012; Sun et al. 2001; Wang et al. 2010; Yu et al. 2011).

As mentioned in the introduction, the AFB_1_-8,9-epoxide may give rise to the AFB_1_-N7-Gua adduct. It is conceivable that genetic polymorphisms affecting the expression of DNA repair enzymes, which could remove the AFB_1_-N7-Gua adduct, could lead to an increased risk to develop a hepatocellular carcinoma. In this context, it has been reported that polymorphisms affecting the gene coding for the X-ray cross-complementing group 1 (XRCC1) protein was associated with a significant increase in the number of AFB_1_-DNA adducts in Taiwanese subjects (Lunn et al. 1999) and an increase in liver cancer risk in Taiwanese subjects (Chen et al. 2005), with an increased *p53* mutation frequency in codon 249 (Long et al. 2008a) and an increase in liver cancer risk in Chinese subjects (Long et al. 2006) and with an increase in liver cancer risk in Gambian subjects (Kirk et al. 2005). In another study from Taiwan, it was reported that *XRCC1* gene polymorphisms alone did not lead to a statistically significant increase in the risk to develop a hepatocellular carcinoma, whereas the liver cancer risk was significantly enhanced in subjects with a *XRCC1* as well as a *GSTT1* gene polymorphism (Yu et al. 2003). Moreover, it has been reported that polymorphisms affecting the genes coding for XRCC3 (Long et al. 2008b), XRCC4 (Long et al. 2013a, b), XRCC7 (Long et al. 2011), XPC (Long et al. 2010) and XPD (Long et al. 2009) were associated with an increased liver cancer risk and enhanced AFB_1_ DNA adduct levels. A study in China (Yao et al. 2014) showed that *XRCC1*, *XRCC3*, *XRCC4*, *XRCC7*, *XPC* and *XPD* gene polymorphisms in combination with a high exposure to AFB_1_ (determined by measuring serum AFB_1_-albumin adduct levels in peripheral blood cells) were associated with an increased risk of developing a hepatocellular carcinoma. A report from Taiwan (Chen et al. 2005) showed that a *hMLH1* polymorphism led to an enhanced liver cancer risk.

##### Conclusions

- There is a linear relationship between the administered AFB_1_ dose and the extent of AFB_1_-DNA binding (i.e. amount of AFB_1_-DNA adducts formed) in rat and rainbow trout liver, even in the low AFB_1_ dose range.
- There is a linear relationship between the administered AFB_1_ dose and the incidence of AFB_1_-induced liver tumours in rat and rainbow trout liver, even in the low AFB_1_ dose range.
- In the case of AFB_1_ no threshold for the formation of hepatic DNA adducts was observed, even down to extremely low dosage (< 1 ng/kg bw)
- There is an obvious and strong influence of promotional effects as exerted by HBV infection (see Table 6)

#### Allylalkoxybenzenes

**Fig. 10:**
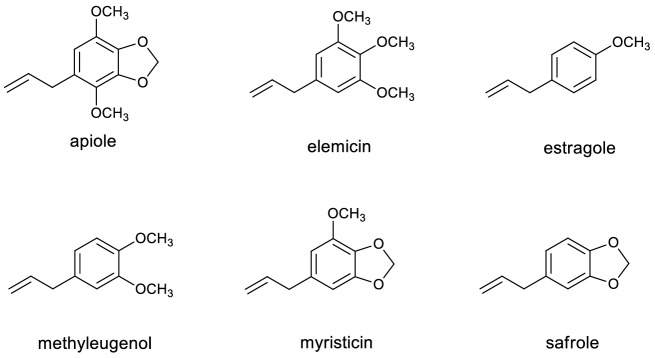
Structures of the related allylalkoxybenzenes

**Fig. 11:**
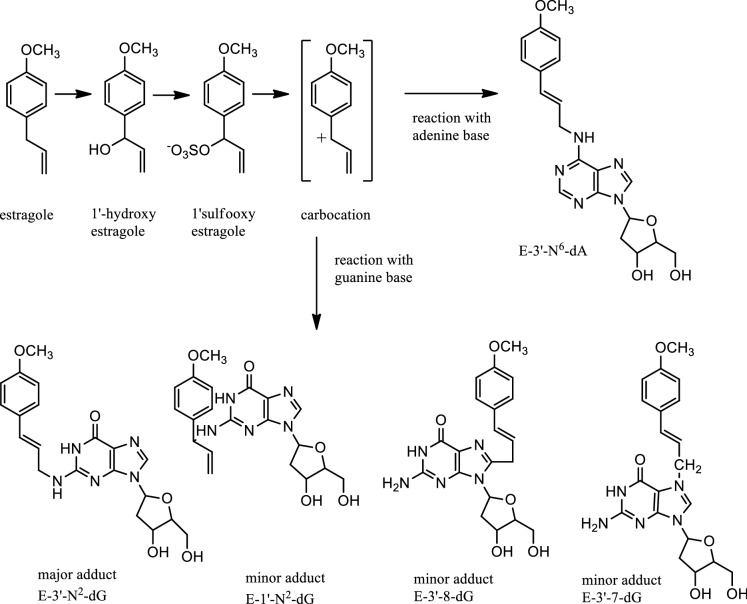
Bioactivation of estragole and formation of the different DNA adducts. *N*^2^-(trans-isoestragol-3′-yl)-deoxyguanosine (E-3′-*N*^2^-dG), *N*^2^-(estragol-1′-yl)-deoxyguanosine (E-1′-*N*^2^-dG), 7-(trans-isoestragol-3′-yl)-deoxyguanosine (E-3′-7-dG), 8-(trans-isoestragol-3′-yl)-deoxyguanosine (E-3′-8-dG), and *N*^6^-(trans-isoestragol-3′-yl)-deoxyadenosine (E-3′-*N*^6^-dA)

Allylalkoxybenzenes are natural ingredients of a variety of herbs and spices, including basil, nutmeg, fennel and many others. They are present in a variety of food items derived from these botanicals including products like pesto and food supplements (SCF 2001a, b, 2002; Van den Berg et al. 2011). Especially alkoxy-substituted allylbenzenes including apiole, elemicin, estragole, methyleugenol, myristicin and safrole (Fig. 10) are converted by cytochrome P450- and sulfotransferase (SULT)-mediated biotransformation via their proximate carcinogenic 1′-hydroxymetabolites to DNA reactive and carcinogenic 1′-sulfooxymetabolites (Fig. 11) (Boberg et al. 1983; Drinkwater et al. 1976; Gardner et al. 1997; Jeurissen et al. 2007; Miller et al. 1983; Phillips et al. 1981; Smith et al. 2002; Wiseman et al. 1985, 1987). Table 7 presents an overview of the genotoxicity and carcinogenicity data on the allylalkoxybenzenes indicating that these compounds are genotoxic carcinogens. The important role for the 1′-sulfooxymetabolite in the DNA adduct and tumour formation by the allylalkoxybenzenes was derived from the observation that intraperitoneal administration of pentachlorophenol, a potent sulfotransferase inhibitor, prior to treatment with estragole, reduced the incidence of animals developing hepatomas to control levels (Wiseman et al. 1987).

**Table 7:** Overview of allylalkoxybenzene genotoxicity and carcinogenicity data

Compound	Carcinogen	Genotoxic	Remarks/ tumour type	References
Apiole	Probably*	Yes	DNA-adducts were detected in HepG2 cells and in vivo. Both dill apiole and parsley apiole did not initiate a significant increase of hepatic tumours	Miller et al. (1983); Phillips et al. (1984); Randerath et al. (1984); Randerath et al. (1985); Zhou et al. (2007)
Elemicin	Probably*	Yes	Elemicin was positive in a DNA-binding assay as well as an UDS assay. No significant increase in the formation of hepatic tumours was found upon administration of elemicin to male mice. 1′-Hydroxyelemicin had hepatocarcinogenic activities at high doses tested	Hasheminejad and Caldwell (1994); Miller et al. (1983); Phillips et al. (1984); Randerath et al. (1984); Randerath et al. (1985); Wiseman et al. (1987)
Estragole	Yes	Yes	Genotoxic and carcinogenic in rodents	EFSA (2009a); Randerath et al. (1984)
Methyleugenol	Yes	Yes	Methyleugenol along with its 1-hydroxy-metabolite is mutagenic in many systems and able to induce DNA adducts and liver tumours in mice	EFSA (2009a); Randerath et al. (1984)
Myristicin	Yes	Yes	Mutagenic and capable of inducing the formation of DNA adducts. 1′-hydroxy-myristicin is considered carcinogenic	EFSA (2009a); Randerath et al. (1984)
Safrole	Yes	Yes	A genotoxic carcinogen. Mutagenic in a variety of assays. A weak carcinogen in rats and mice, and a trans-placental carcinogen in mice	Boberg et al. (1983); EFSA (2009a); Miller et al. (1983); Randerath et al. (1984)

*Structurally related allylalkoxybenzenes are carcinogenic

##### DNA adducts of allylalkoxybenzenes

Several adducts are formed upon reaction of the 1′-sulfooxymetabolites of allylalkoxybenzenes with DNA. As an example Fig. 11 presents the DNA adducts of 1′-sulfooxyestragole. These adducts include *N*^2^-(trans-isoestragol-3′-yl)-deoxyguanosine (E-3′-*N*^2^-dG), *N*^2^-(estragol-1′-yl)-deoxyguanosine (E-1′-*N*^2^-dG), 7-(trans-isoestragol-3′-yl)-deoxyguanosine (E-3′-7-dG), 8-(trans-isoestragol-3′-yl)-deoxyguanosine (E-3′-8-dG), and *N*^6^-(trans-isoestragol-3′-yl)-deoxyadenosine (E-3′-*N*^6^-dA) (Ishii et al. 2011; Phillips et al. 1981; Punt et al. 2007; Suzuki et al. 2012). The major adduct formed with the guanine base is *N*^2^-(trans-isoestragol-3′-yl)-deoxyguanosine (E-3′-*N*^2^-dG) which is considered to play a role in the genotoxic and carcinogenic effects induced by estragole (Phillips et al. 1981; Smith et al. 2002). It was reported that adducts between estragole and adenine (E-3′-*N*^6^-dA) may also be formed to a significant extent in the liver of male rats (F344) exposed to estragole at a dose level of 600 mg/kg bw for 4 weeks (Ishii et al. 2011; Suzuki et al. 2012). However, at a lower dose of 22 mg estragole/kg bw the major adduct measured was the E-3′-*N*^2^-dGuo (Suzuki et al. 2012). This indicates that dose-dependent changes can occur in the type of adduct formed, but that at dose-levels relevant for dietary human intake E-3′-*N*^2^-dGuo adducts may be most relevant.

##### Genotoxicity/mutagenicity of allylalkoxybenzenes

In spite of this clear evidence for the formation of DNA adducts, allylalkoxybenzenes generally tested negative in most standard genotoxicity assays including the Ames test (Ding et al. 2011; Mortelmans et al. 1986; NTP 2000; SCF 2001a, b, 2002; Sekizawa and Shibamoto 1982), and several in vivo genotoxicity tests (Ding et al. 2011; NTP 2000). For example, a 14 week in vivo study, in which mice were dosed orally with methyleugenol, showed no increase in the frequency of micronucleated normochromatic erythrocytes (NTP 2000). The absence of positive results in genotoxicity tests may to some extent be ascribed to the absence of the relevant enzymes, especially the sulfotransferases (SULTs), required for conversion of the allylalkoxybenzenes into their ultimate carcinogenic 1′-sulfooxy metabolites (Fig. 11). This was confirmed by studies using *Salmonella typhimurium* TA100 strains with the expression of human SULT which, especially upon expression of human SULT1A1 and SULT1C2, appeared able to activate 1′-hydroxymethyleugenol to DNA reactive metabolites resulting in positive Ames test results (Herrmann et al. 2012).

##### Role of DNA adduct formation in tumour formation by allylalkoxybenzenes

DNA adduct formation, although involved in the process of tumour formation, is generally considered a biomarker of exposure rather than a biomarker of effect (Brink et al. 2009; La and Swenberg 1996; Swenberg et al. 2008; Williams 2008) although it is also well recognized that increased levels of DNA adduct formation reflect a risk factor in cancer development. For the allylalkoxybenzenes the formation of DNA adducts is considered important in the mode of action underlying the tumour induction. Several attempts have been made to correlate the occurrence of DNA adducts with the carcinogenic outcome, but the significance of their formation in the risk assessment, especially with respect to the discussion and justification of possible thresholds is a matter of ongoing debate (Jarabek et al. 2009; Neumann 2009). Comparison of the level of DNA adducts estimated to be formed at the BMD_10_ for methyleugenol were actually below or close to the background DNA adduct level set at 100 adducts in 10^8^ nt (Paini et al. 2011). Theoretically this observation of DNA adduct formation at the BMD_10_ at levels that are below the background levels of DNA adduct formation might reflect that either the DNA adducts formed are far more mutagenic and carcinogenic than the type of lesions present in the background levels or it might imply that the mode of action underlying the tumour induction includes an additional mechanism in addition to DNA adduct formation such as for example cytotoxicity. For methyleugenol literature data point at a possible role for liver toxicity in addition to DNA adduct formation in the mechanism underlying tumour formation (FAO/WHO 2010).

##### (Non)-linearity of concentration- or dose–response curves for allylalkoxybenzenes

**Fig. 12:**
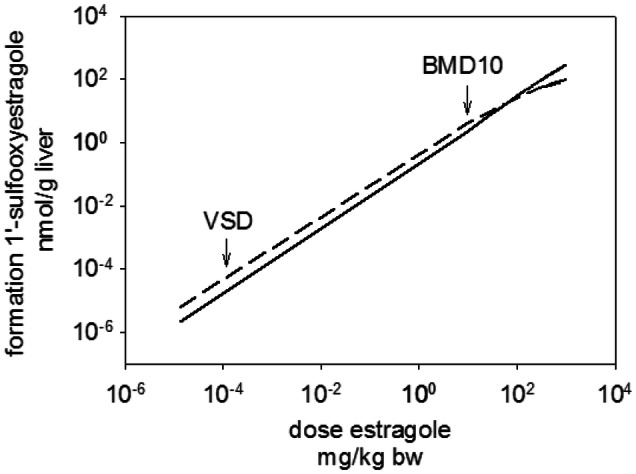
PBK model-predicted dose-dependent formation of 1′-sulfooxyestragole in the liver of rat (─) and human (- -), also indicating the Benchmark dose 10 (BMD_10_) representing the dose level resulting in a tumour incidence of 10% above background level and the Virtual Safe Dose (VSD), calculated by linear extrapolation to represent a dose level causing one in a million tumour incidence above background level. Reproduced with permission from Rietjens et al. (2010). Copyright (2009) WILEY–VCH Verlag GmbH & Co. KGaA, Weinheim

In accordance with the current scientific view on low dose linearity for DNA adduct formation (Neumann 2009; Paini et al. 2011; Swenberg et al. 2008) the dose–response curve for DNA adduct formation of allylalkoxybenzenes seems to be linear through the origin (Ellis et al. 2006; Paini et al. 2011). Furthermore, in primary hepatocytes exposed to 1′-hydroxyestragole, E-3′-*N*^2^-dG adduct formation also increased linearly through the origin with increasing concentration of 1′-hydroxyestragole (Paini et al. 2010). In addition, it was demonstrated that the level of E-3′-*N*^2^-dG adducts detected in the liver of male Sprague Dawley rats also showed a linear correlation with dose (Paini et al. 2012). In line with this the formation of the unstable 1′-sulfooxyestragole (in nmol/g liver), predicted by a rat physiologically based kinetic (PBK) model, also showed a linear dose–response curve (Paini et al. 2012). These PBK models, by now developed and validated for a variety of related allylalkoxybenzenes (Al-Subeihi et al. 2012, 2011; Martati et al. 2011, 2012; Punt et al. 2008, 2009; Van den Berg et al. 2012) have revealed that formation of the 1′-sulfooxymetabolites of allylalkoxybenzenes in human and rat liver is predicted to be linear from doses as low as the normal dietary human intake up to doses as high as the BMD_10_ (benchmark dose that gives 10% extra cancer incidence) (Martati et al. 2012; Rietjens et al. 2010; Van den Berg et al. 2012) (Fig. 12). Whether this translates into linearity for tumour formation remains however to be established.

##### Conclusions

- There is a linear relationship through the origin between the dose of an allylalkoxybenzene and its levels of bioactivation to the DNA reactive metabolite and also to the level of DNA adduct formation in animal experiments
- This linearity holds from dose levels as high as the BMD_10_ (benchmark dose that gives 10% extra cancer incidence) down to dose levels as low as dietary human intake.
- Genotoxicity/mutagenicity bioassays may give false-negative results when adequate enzymes are lacking.
- Physiologically based kinetic (PBK) modelling provides insight in the form of the dose–response curve for the formation of unstable DNA reactive metabolites even at dose levels that are experimentally not accessible.
- In addition to DNA adduct formation, cytotoxicity may add to the mode of action underlying the tumour formation by allylalkoxybenzenes.

#### 2-Amino-3,8-dimethylimidazo[4,5-*f*]quinoxaline (MeIQx)

##### Introduction

**Fig. 13:**
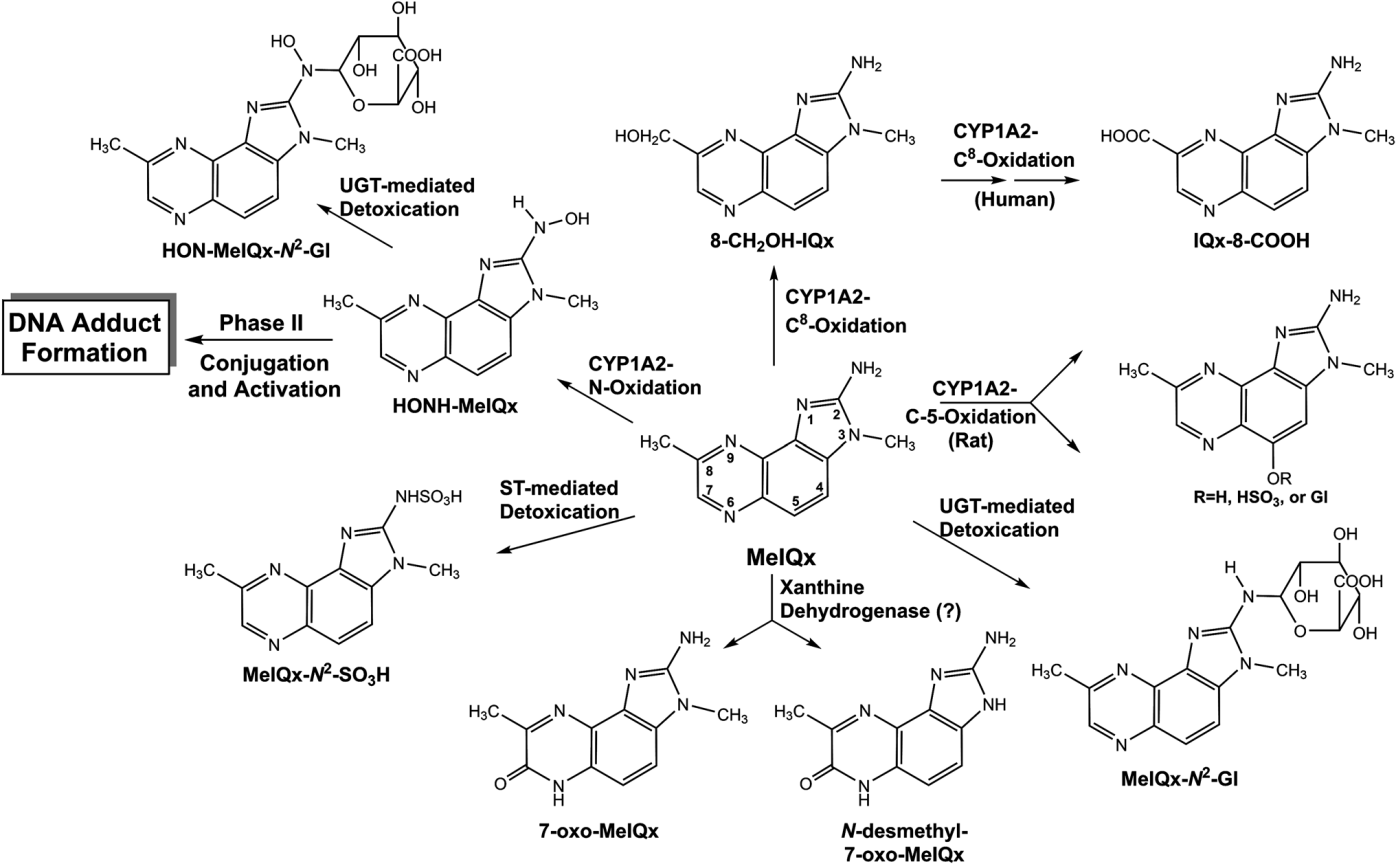
Metabolic activation and detoxification of 2-amino-3,8-dimethylimidazo[4,5-*f*] quinoxaline (MeIQx). Reprinted from Turesky et al. (2002), Copyright (2002), with permission from Elsevier.

**Fig. 14:**
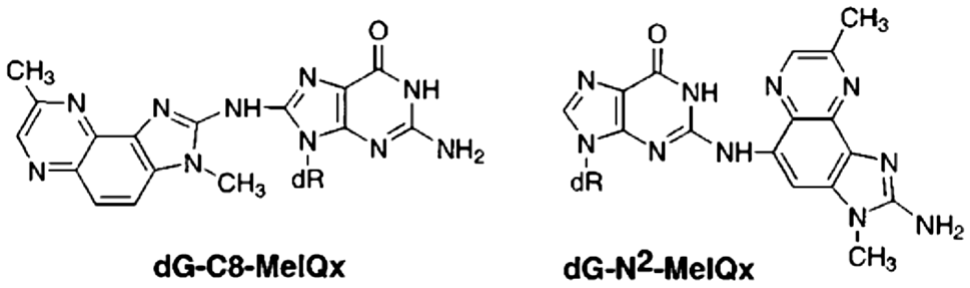
Structures of *N*-(deoxyguanosin-8-yl)-2-amino-3,8-dimethylimidazo[4,5-*f*] quinoxaline (dG-C8-MeIQx) and 2-amino-3,8-dimethylimidazo[4,5-*f*]quinoxaline (dG-*N*^2^-MeIQx) (reproduced from Paehler et al. 2002)

Heterocyclic aromatic amines (HAA) are formed in red meat and fish during the cooking procedure at high temperatures for a long time or over an open fire through pyrolysis reactions between amino acids, glucose and creat(in)ine (Felton and Knize 1990; Knize et al. 1994; Skog and Jagerstad 1993; Wakabayashi et al. 1992). Under most of the cooking conditions and from a quantitative point of view 2-amino-3,8-dimethylimidazo[4,5-*f*]quinoxaline (MeIQx) is the second most prevalent HAA in heated food items (Felton and Knize 1990; Skog and Jagerstad 1993). MeIQx per se is biologically inactive. To exert its genotoxicity and carcinogenicity, it must first be metabolically activated (Fig. 13) (Turesky et al. 2002). A cytochrome P450-mediated *N*-hydroxylation of MeIQx followed by conjugation of the *N*-hydroxy moiety to any of several leaving groups, such as acetate or sulfate, results in covalent binding to DNA (Paehler et al. 2002; Rich et al. 1992; Sjodin et al. 1989; Solomon et al. 1996; Turesky et al. 1988, 1991a, b, 1992). In vitro* N*-(deoxyguanosin-8-yl**)**-MeIQx (dG-C8-MeIQx) is the major and 5-(deoxyguanosin-*N*^2^-yl)-MeIQx the minor adduct (Turesky et al. 1992) (Fig. 14). In contrast, in vivo and in the case of administering a low dose of MeIQx to rats 5-(deoxyguanosin-*N*^2^-yl)-MeIQx (dG-*N*^2^-MeIQx) seems to significantly contribute to the genotoxic damage elicited by MeIQx (Paehler et al. 2002).

##### MeIQx-DNA adduct formation in the liver of rats

Yamashita et al. (1990) analysed the formation of MeIQx-DNA adducts in the liver of male Fischer 344 rats after dietary administration of high to extremely high levels of MeIQx (i.e. 0.4, 4, 40 and 400 ppm, equivalent to 0.036, 0.36, 3.6 and 36 mg/kg bw/day) for up to 12 weeks by ^32^P-postlabeling. MeIQx-DNA adducts in rat liver increased time-and dose-dependently, the relationship between the administered dose of MeIQx and the detected MeIQx-DNA levels being clearly linear (Yamashita et al. 1990). A later study by the same group showed that MeIQx-DNA adducts formed are very persistent, their removal occurring in a biphasic manner, i.e. an initial rapid removal is followed by a phase of slow changes in MeIQx-DNA adduct levels (Hirose et al. 1995b). If male Sprague–Dawley rats were exposed to very low dietary doses of MeIQx (dose range: 0.000001–0.034 ppm) for 1, 7 or 42 days, a linear correlation between the administered doses and the detected hepatic MeIQx-DNA adduct levels was again observed and the adducts persisted for at least 14 days after the exposure ceased (Frantz et al. 1995; Turteltaub et al. 1997).

It has been shown in the past that synthetic antioxidants are able to inhibit MeIQx-induced hepatocarcinogenesis in rats (Hirose et al. 1995a, 1998a, b). Based on these findings, it has been suggested that oxygen-derived free radicals may play an important role in MeIQx-mediated tumour development. In three studies, hepatic 8-hydroxyguanine levels have been measured following the dietary administration of MeIQx to male Fischer 344 rats (Fukushima et al. 2002; Kato et al. 1996; Murai et al. 2008). Kato et al. (1996) exposed rats to increasing concentrations (0.05, 0.2, 0.8, 3.2, 12.5, 50 and 200 ppm, equivalent to 0.006, 0.024, 0.096, 0.384, 1.5, 6 and 24 mg/kg bw/day) of MeIQx in the diet for six weeks and the animals were partially hepatectomised 1 week after beginning to expose the animals to MeIQx. The 8-hydroxyguanine levels in the resected liver samples (i.e. at week 1) increased in a concentration-dependent manner, but the correlation between the MeIQx doses and the 8-hydroguanine levels was not linear. Surprisingly, the hepatic 8-hydroxyguanine levels at the end of the exposure period (i.e. at week 6) were similar to those in control animals, independently of the administered dose. This might be due to an induction of DNA repair enzymes in the course of the 6-week feeding period, but this possibility has not been proven experimentally up to now. In a second study, Fukushima et al. (2002) fed rats MeIQx at dietary dose levels of 0.001, 0.01, 0.1, 1, 10 and 100 ppm (equivalent to 0.00005, 0.0005, 0.005, 0.05, 0.5 and 5 mg/kg bw/day) for 16 or 32 weeks. A statistically significant increase in the 8-hydroxyguanine levels when compared to those in control animals was only observed with 100 ppm MeIQx. In a third study, Murai et al. (2008) reported that in rats fed MeIQx at dietary levels of 0.001, 1 and 100 ppm (equivalent to 0.00005, 0.05 and 5 mg/kg bw/day) for up to 104 weeks the hepatic 8-hydroxyguanine levels did not increase at all if compared to those of control rats. Taken together, the data sets on the relationship between the administered MeIQx doses and the detected hepatic 8-hydroxyguanine levels are inconsistent and at this stage do not allow any conclusion on the relevance of the 8-hydroxyguanine levels in the MeIQx-mediated hepatocarcinogenic process.

##### Induction of preneoplastic and neoplastic lesions in the liver of MeIQx-fed rats

The hepatocarcinogenic potential of MeIQx was tested in male Fischer 344 rats exposed to dietary doses of 100, 200 and 400 ppm (equivalent to 5, 10 and 20 mg/kg bw/day) (Kushida et al. 1994). The incidence of hepatocellular carcinomas in these three groups was 0, 45 and 94%, respectively, and based on these results the authors described the correlation between the administered MeIQx doses and the hepatocellular carcinoma incidences as non-linear.

In later studies by Japanese researchers (Fukushima 1999; Fukushima et al. 2003, 2016; Hoshi et al. 2004; Wei et al. 2006), MeIQx was tested at much lower doses, but the biological endpoint quantified in the above-mentioned studies was the number of glutathione *S*-transferase P (GST-P)-positive foci per cm^2^ hepatic tissue instead of the incidence of hepatocellular carcinomas. GST-P-positive foci are described as being preneoplastic hepatic lesions that very well correlate with liver cancer induction in rats (Ogiso et al. 1990) and have the advantage that they are detected very early in the hepatocarcinogenic process (i.e. the feeding experiments can be strongly shortened). Fukushima (1999), Fukushima et al. (2016) and Hoshi et al. (2004) fed male Fischer 344 0.001, 0.01, 0.1, 1, 10 or 100 ppm MeIQx (equivalent to 0.00005, 0.0005, 0.005, 0.05, 0.5 or 5 mg MeIQx/kg bw/day) for 16 weeks. The number of GST-P-positive liver foci per cm^2^ in animals fed 0.001–1 ppm MeIQx was similar to that in control rats, whereas it increased if animals were fed 10 or 100 ppm (Fukushima 1999; Hoshi et al. 2004) (only at 100 ppm in the case of Fukushima et al. (2016)). In male BN rats fed 0.1, 1, 5, 10 or 100 ppm MeIQx (equivalent to 0.005, 0.05, 0.25, 0.5 or 5 mg MeIQx/kg bw/day) for 16 weeks, the number of GST-P-positive liver foci per cm^2^ in animals fed 0.1–10 ppm MeIQx was similar to that in control rats, whereas it increased significantly in animals fed 100 ppm (Wei et al. 2006). If male Fischer 344 rats were fed 0.001, 0.01, 0.1, 1, 10 or 100 ppm MeIQx (equivalent to 0.00012, 0.0012, 0.012, 0.12, 1.2 or 12 mg MeIQx/kg bw/day) for 4 weeks and thereafter exposed to the tumour promoter phenobarbital for 11 weeks (Fukushima et al. 2003), the number of GST-P-positive liver foci per cm^2^ in animals fed 0.001–1 ppm MeIQx was similar to that in control rats, whereas it increased if animals were fed 10 or 100 ppm. The observation that MeIQx only induces liver tumours in rats when administered in extremely high (totally human-irrelevant) doses is also observed with 2-amino-1-methyl-6-phenylimidazo[4,5-*b*]pyridine (PhIP), the quantitatively most important HAA in strongly heated fish and meat samples (Fukushima et al. 2004; Hasegawa et al. 1993; Ito et al. 1991).

Hoshi et al. (2004) reported that the mutation frequency in the liver of male Fischer 344 rats fed 0.001, 0.01, 0.1, 1, 10 or 100 ppm MeIQx (equivalent to 0.00005, 0.0005, 0.005, 0.05, 0.5 or 5 mg MeIQx/kg bw/day) for 16 weeks was 14.9, 15.6, 19.9, 29.4, 51.4 and 641.5 mutants/10^6^ nucleotides, respectively, only the values in the 10 and 100 ppm group being significantly increased when compared to the control group. Furthermore, the liver cell proliferation rate was similar in all experimental groups (Hoshi et al. 2004).

Based on the above-mentioned results (Fukushima 1999; Fukushima et al. 2016, 2003; Hoshi et al. 2004; Wei et al. 2006) it has been claimed that a threshold for the induction of liver tumours does in fact exist. However, the results obtained may very well be explained by the fact that MeIQx either lacks or possesses a very weak tumour promoting activity. Kleman et al. (1993) showed that MeIQx was a weak initiator and did not promote the growth of diethylnitrosamine-initiated GST-P-positive liver foci in a short-term rat liver carcinogenesis model. Moreover, in a newborn mouse two-stage tumorigenesis assay the tumour promoting effect of MeIQx also proved to be weak (Miyauchi et al. 1999). In line with these studies it has been demonstrated that low doses of MeIQx can indeed lead to the increased formation of GST-P-positive liver foci if a strong liver cell proliferation (i.e. a strong promoting effect) is induced in rats by carbon tetrachloride (Sone et al. 1992) or a choline-deficient diet (Sone et al. 1993) or takes place as a consequence of a genetic alteration, as in Long-Evans with cinnamon-like coat colour (LEC) rats, which have a mutation in the copper transporting ATPase gene Atp7b, accumulate high levels of copper in the liver and suffer from hereditary hepatitis leading in the end to liver cancer (Sone et al. 1996). Moreover, as previously pointed out by Bailey et al. (2009) the absence of background corrections, the variability in response and the limited number of animals in the previously cited studies (Fukushima 1999; Fukushima et al. 2003; Hoshi et al. 2004; Wei et al. 2006) does not allow to conclude that in the case of MeIQx a threshold for the induction of liver tumours exists.

##### Conclusions

- There is a linear correlation between the administered MeIQx doses and the extent of MeIQx-DNA binding (i.e. amount of MeIQx-DNA adducts formed) in the liver of rats exposed to MeIQx, even in the low dose range.
- There is no clear-cut correlation between the administered MeIQx doses and the 8-hydroxyguanine levels in the liver of rats exposed to MeIQx.
- Hepatic preneoplastic and neoplastic lesions were only induced if rats were exposed to very high doses of the HAA, i.e. in this case no linear correlation between the administered MeIQx doses and the induction of preneoplastic and neoplastic lesions in the rat liver is observed. This is most probably due to the fact that MeIQx lacks or only possesses a very weak tumour promoting activity. Moreover, it must be pointed out that the studies supporting the existence of a threshold for the MeIQx-mediated induction of liver tumours show serious methodological limitations and, therefore, do not allow to reach such a conclusion.

#### Benzo[*a*]pyrene

Benzo[*a*]pyrene (BaP) belongs to the class of polycyclic aromatic hydrocarbons (PAH) which are widespread environmental and food contaminants. PAH are continuously formed during incomplete combustion or pyrolysis of organic material and are thus present in the ambient air, water, soils and sediments. Major environmental sources are heating, motor-vehicle exhaust and industrial emissions, reaching levels of 1–30 ng/m^3^, with considerably higher levels in road tunnels and large cities with extensive use of coal for residential heating. Significant exposures for the general population result furthermore from tobacco smoke and food, especially barbecued, grilled, broiled and smoke-cured meats. Highest levels of workplace exposure to PAHs are observed in aluminium production (Söderberg process) with values up to 100 μg/m^3^. Mid-range levels are observed in roofing and paving (e.g. 10–20 μg/m^3^) and concentrations at or below 1 μg/m^3^ are observed in coal liquefaction, coal-tar distillation, wood impregnation, chimney sweeping and power plants (IARC 2010b).

##### Carcinogenicity

Since BaP is only one component of PAH mixtures of different compositions, no epidemiological data exist for BaP alone with respect to carcinogenicity. Nevertheless, based on strong and consistent evidence of carcinogenicity of BaP in many animal species after basically all routes of exposure, supported by consistent and coherent mechanistic information, BaP has been classified as carcinogenic to humans (Group 1) by IARC (2010b) and in carcinogen group 2 by the German MAK Commission (Hartwig 2013c).

##### DNA adducts and mutagenicity

**Fig. 15:**
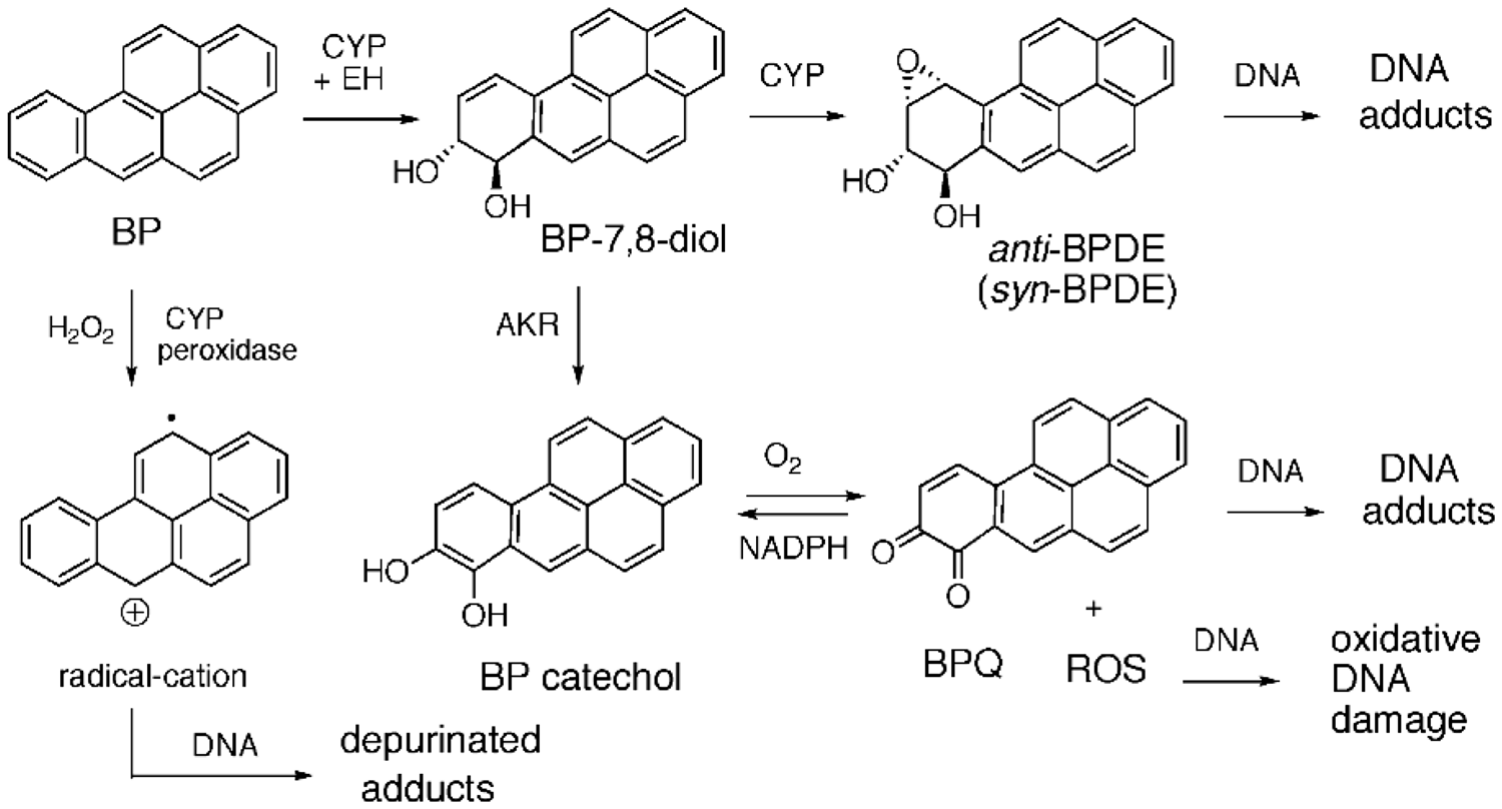
Metabolism of Benzo[*a*]pyrene (Harvey et al. 2005), reprinted by permission of the publisher (Taylor & Francis Ltd, https://www.tandfonline.com)

The carcinogenic activity of BaP is attributed to the formation of DNA adducts, resulting from electrophilic attack predominantly at guanine residues by metabolically activated intermediates formed from the parent hydrocarbon. Routes of metabolic activation include the formation of radical cations via P450 and/or peroxidases and the formation of *o*-quinones via dihydrodiol dehydrogenases (Fig. 15).

For carcinogenicity, the probably most relevant metabolic pathway is connected to the action of cytochromes P450 1A1 and 1B1 and epoxide hydrolase, yielding *syn*- and *anti-*B[*a*]P-7,8-diol 9,10-epoxides (BPDE), which form adducts at the *N*^2^ position of guanine. In contrast to BaP-induced lesions at positions N7 or C8 of purines derived from radical cations, which give rise to apurinic sites due to hydrolysis of the N-glycosidic bond, hydrolysis of BPDE-induced DNA adducts is very slow and they are considered to be stable on cellular conditions. These lesions are substrates of nucleotide excision repair (NER) (Camenisch and Naegeli 2009; Hess et al. 1997). When DNA is replicated prior to their removal, they can lead to mutations and cancer (Melendez-Colon et al. 1999).

**Fig. 16:**
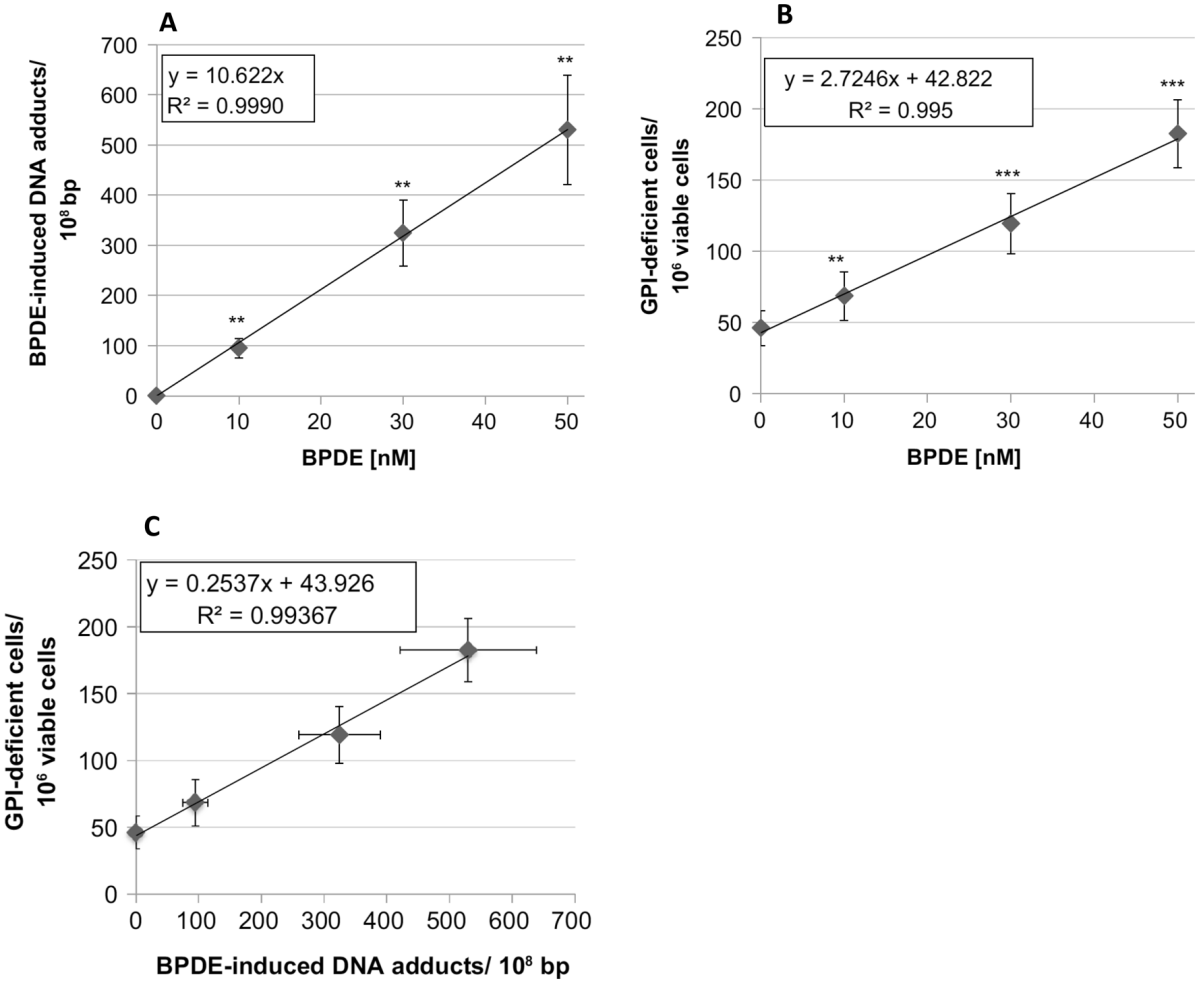
BPDE-induced DNA adduct levels determined via HPLC/Fluorescence detection (**a**), mutation frequencies determined by PIG-A assay (**b**), and the correlation between DNA adducts and mutation frequencies (**c**). For details see Piberger et al. (2017)

Within a recent study we specifically addressed the question on dose–response relationship in the low dose range for BaP-induced stable DNA adducts in the *N*^2^-position of guanine, their repair and the induction of mutations, and compared it to the onset of the DNA damage response on the transcriptional level (Piberger et al. 2017). All endpoints were investigated in the same cell line, namely TK6 cells. To exclude cellular detoxification of BaP preceding the induction of DNA lesions, cells were treated with its DNA reactive metabolite, ( +)-*anti*-benzo[*a*]pyrene 7,8-diol-9,10-epoxide (( +)-*anti*-BPDE). Mutations were quantified via the in vitro PIG-A mutagenicity test, which has been recently established for TK6 cells (Krüger et al. 2015). Quantification of ( +)-*anti*-BPDE-induced DNA adducts was performed via a highly sensitive HPLC-based assay coupled with fluorescence detection, enabling the detection of the respective tetrol I-1 upon hydrolysis of DNA adducts in the very low dose range (Schwerdtle et al. 2002). Finally, a high-throughput RT-qPCR approach was applied to quantitatively elucidate the onset of the transcriptional DNA damage response at the same conditions (Fischer et al. 2016). The results demonstrate a linear dose–response relationship of DNA adducts, detectable at concentrations as low as 10 nM BPDE. Furthermore, a linear increase in mutations was observed in the same dose range. In addition, a linear correlation between the amounts of DNA adducts and mutations was observed, indicating no threshold-like effect for the conversion of DNA adducts into mutations (Fig. 16).

To exclude that low-dose mutagenicity was due to diminished repair in this cell line, the time course of DNA lesion removal was elucidated. Within 8 h post-incubation time, only 30% of lesions were removed, and 40% were still left after 24 h. This repair course is in agreement with previous observations in A549 and HCT116 cells (Grosskopf et al. 2010; Piberger et al. 2014; Schwerdtle et al. 2002), supporting similar repair capacities in different cell lines. Interestingly, repair kinetics were independent of the applied dose, indicating that within this low dose range of BPDE, no differences in relative repair were observed and thus no dose showed faster or even complete repair. This may be explained by specific features of NER mediating the repair of stable BPDE-DNA adducts. NER removes structurally unrelated bulky base adducts generating significant helical distortions. It involves at least 30 different proteins and enzymes in mammalian cells, including those which are defective in patients suffering from the DNA repair disorder Xeroderma Pigmentosum (XP) complementation groups A through G (de Boer and Hoeijmakers 2000). Two different pathways can be distinguished: the global genome repair (GG-NER) operating in all parts of the genome and the transcription-coupled repair (TC-NER) eliminating DNA damage from the transcribed strand of active genes. While TC-NER is usually fast and efficient to restore transcription, GG-NER on the other hand is slower and may be incomplete, leading to an accumulation of mutations in poorly repaired regions (Fousteri and Mullenders 2008; Mullenders et al. 1991). Accordingly, three levels of repair efficiencies have been identified in human fibroblasts after treatment with BPDE: The transcribed strand of the active *HPRT* gene was repaired fastest, followed by the non-transcribed strand, while only a small fraction of BPDE adducts were removed from the inactive locus *754* within 20 h (Chen et al. 1992). In addition, the rates of incision of stereochemically identical BPDE-induced DNA lesions catalysed by the prokaryotic UvrABC system was shown to be higher in the TG*T than in the CG*C sequence context (Ruan et al. 2007). The longevity of at least some PAH-induced DNA adducts was also shown in lung autopsy samples of non-smokers, ex-smokers, and smokers. Lowest frequencies of lesions were found in the first group, intermediate frequencies in the second, and highest values in the third group. Furthermore, almost all samples even of the non-smoking group had detectable levels of PAH-induced DNA lesions, indicating that even low levels of environmental exposure lead to unrepaired DNA adducts (Lodovici et al. 1998).

##### Transcriptional response to DNA damage

To elucidate the transcriptional response to DNA damage on the same conditions in the same cell line, gene expression analysis was conducted in the very low concentration range of 10 to 200 nM BPDE, by applying a high-throughput RT-qPCR technique, enabling quantitative time- and concentration-dependent gene expression analyses for 96 samples in parallel, and providing a comprehensive gene expression profile with respect to DNA damage response, DNA repair factors, oxidative stress response, cell cycle arrest, cell proliferation, and apoptosis. As expected, treatment with BPDE induced genes coding for DNA damage signalling such as *GADD45A*, DNA repair factors involved in DNA damage recognition during NER, p53 and AP-1 dependent signalling, as well as those coding for oxidative stress response and pro-apoptotic factors. However, almost all significant changes in gene expression were restricted to the two highest concentrations applied, 100 and 200 nM BPDE, while highly significant increases in mutation frequencies were observed at concentration levels 10- and 20-fold lower. Therefore, neither the induction of DNA repair genes nor for example p53-dependent cell cycle control or apoptotic genes were able to protect against BPDE-induced mutations in the very low dose range (Piberger et al. 2017).

##### Conclusions and Outlook

Taken together, BPDE increased the level of GPI-deficient mutant cells in a dose-dependent manner, with no obvious deviation from linearity also at the lowest concentrations. Furthermore, there was a linear correlation between DNA adduct formation and mutagenicity, again arguing against a “no effect” range in the low dose exposure towards BPDE. In contrast, the transcriptional response to DNA damage was restricted to higher, partly cytotoxic concentrations. One reason for the discrepancy between the linear correlation of DNA damage and mutations shown here and the reported “threshold” in response to some alkylating agents appears to be the impact of different types of DNA lesions, especially also different types of DNA repair systems involved in their removal. Even though nucleotide excision repair is a largely error-free process, which protects from mutagenicity, it has been shown to be slower and less effective as compared to base excision repair, due to heterogeneity of repair throughout the genome and also with respect to the DNA sequence in which the lesion is located. Nevertheless, even though DNA repair capacities were found to be similar in different cells lines, they may differ in vivo in different tissues; this needs to be further investigated. This aspect also accounts for the transcriptional response to DNA damage. Whether or not our observations derived for treatment with BPDE also apply to other substrates of NER requires future research.

#### Pyrrolizidine alkaloids

##### Introduction

Pyrrolizidine alkaloids (PAs) are a huge group of natural plant phytochemicals. Many PAs are known to be highly hepatotoxic, and some have been shown to be carcinogenic in laboratory animals. PAs are found in many plants around the world, in particular in plants of the families *Boraginaceae*, *Asteraceae* and *Fabaceae*. It is estimated that approximately 3% of the world’s flowering plants contain one or more toxic PAs (COT 2008; Fu et al. 2004; Mattocks 1986; Stegelmeier et al. 1999). Possible food sources of human exposure are herbal remedies and teas, contaminated foods such as grain crops and honey, as well as food supplements that contain PA-containing plants (BfR 2011, 2013; EFSA 2011a).

**Fig. 17:**
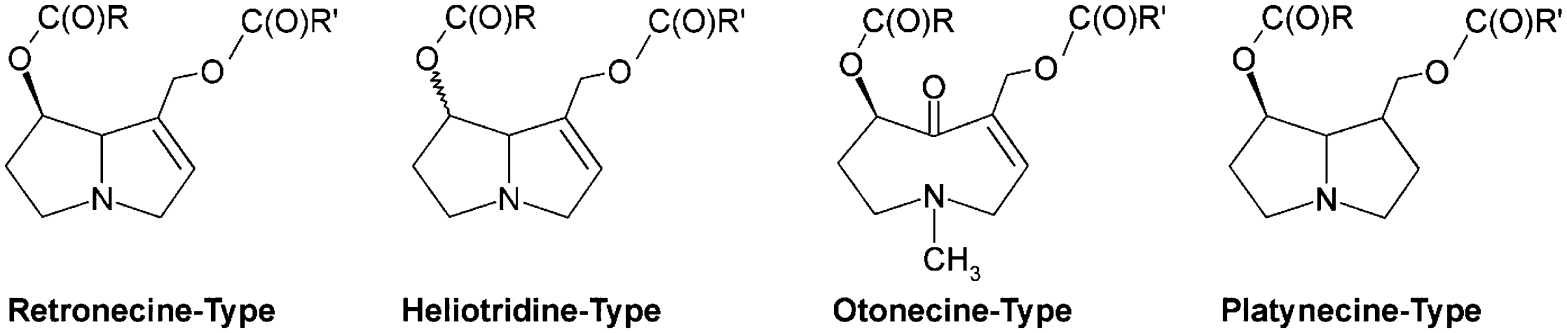
Structures of the representative necine bases, retronecine, heliotridine, otonecine and platynecine, that form the basis of a variety of pyrrolizidine alkaloids

**Fig. 18:**
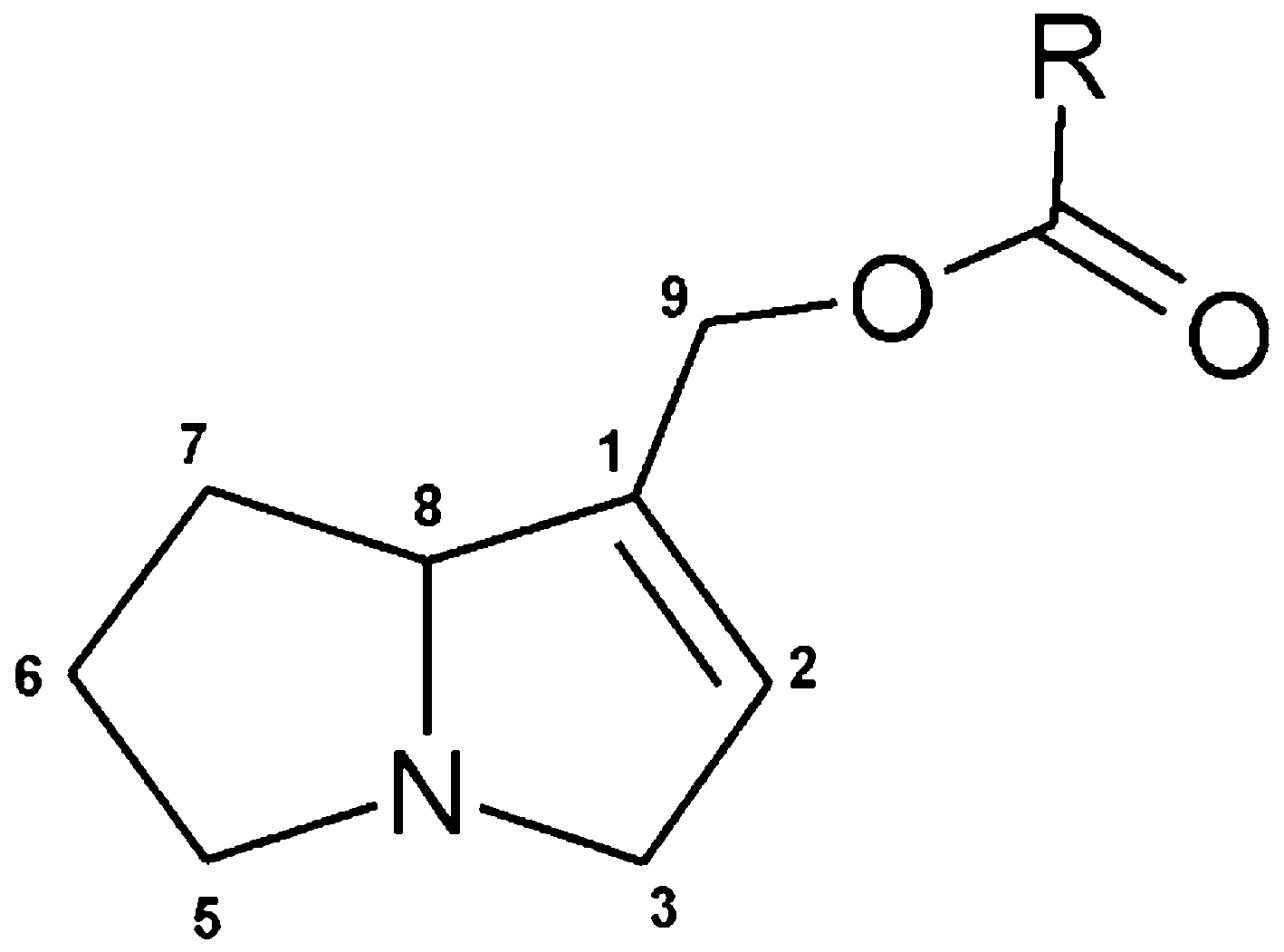
Generic structure required for pyrrolizidine alkaloids to cause toxicity (reproduced from COT 2008)

PAs are heterocyclic compounds, sharing a basic structure derived from esters of four necine bases: retronecine, heliotridine, otonecine and platynecine (Fig. 17). The acid moieties of the esters are termed necic acid. To date, all of the known tumorigenic PAs are based upon a retronecine, heliotridine, or otonecine basic structure (Fu et al. 2004). A number of structural features determine the toxic properties of PAs (see Fig. 18). PAs correlated with adverse effects are esters of 1-hydroxymethyl-1,2-dehydropyrrolizidine (1,2-unsaturated PAs). There may be a second hydroxyl group at the C7 position. At least one of these hydroxyl groups must be esterified to exert toxicity and the acid moiety of the ester linkage must contain a branched chain (COT 2008).

**Fig. 19:**
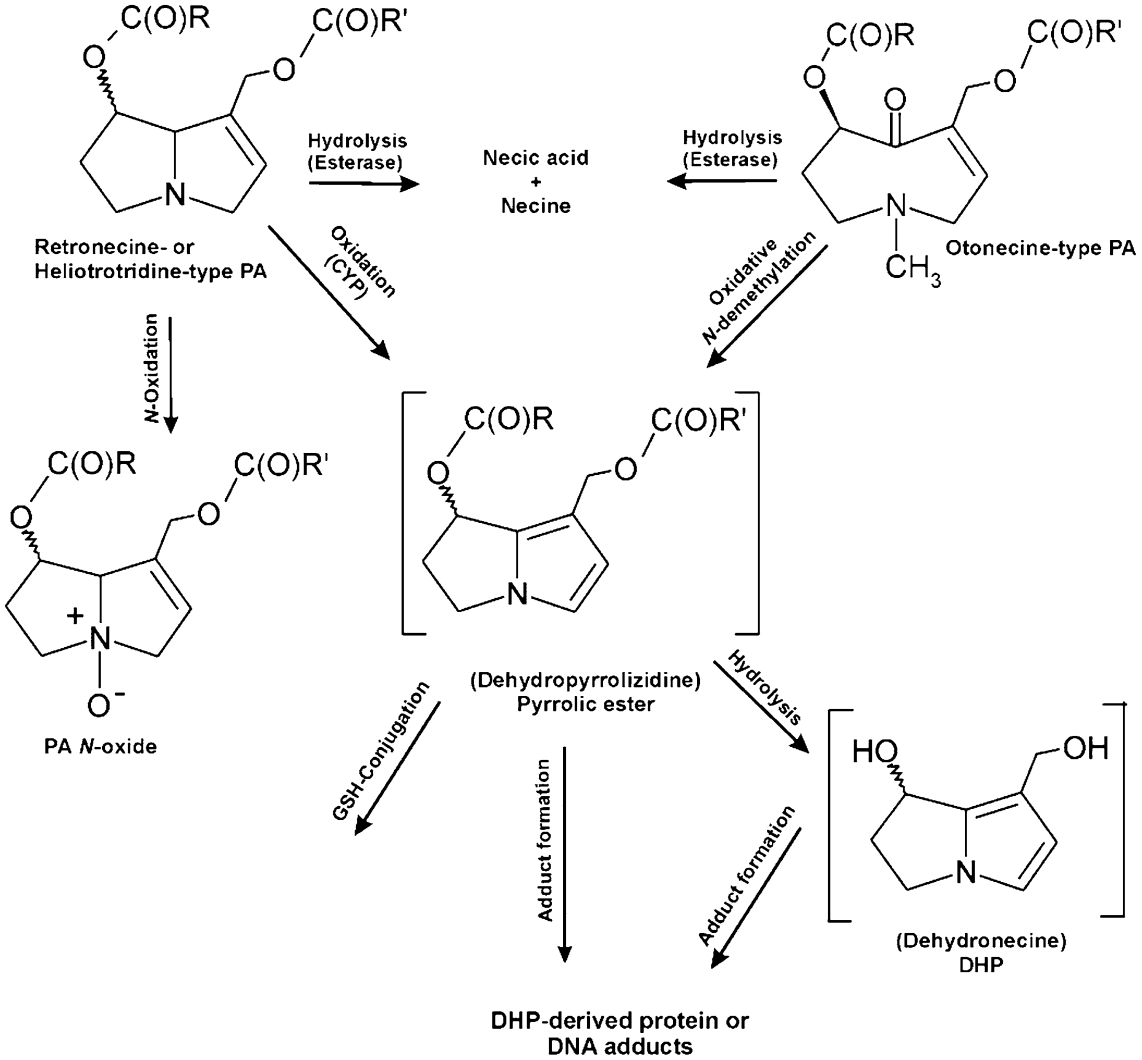
Metabolic activation and detoxification of pyrrolizidine alkaloids (Chen et al. 2010b). Reprinted by permission of the publisher (John Wiley & Sons, Ltd)

PAs require metabolic activation to exert their genotoxicity and tumorigenicity (Fig. 19). The activation pathway is oxidation at the C3 or C8 position of the necine base (catalysed mainly by hepatic CYP) resulting in the formation of the corresponding pyrrolic esters, which are chemically reactive and can act as alkylating agents towards proteins and DNA. Pyrrolic esters can undergo further biotransformation by glutathione conjugation (Phase II metabolism) or can be further hydrolysed at the ester bond to form the free pyrrole base, often referred as DHP (6,7-dihydro-7-hydroxy-1-hydroxymethyl-5[H]-pyrrolizine). While DHP is less reactive than the parent pyrrolic esters, it is still unstable in aqueous solutions and retains considerable alkylating activity. The PA detoxification pathways include the esterase-mediated cleavage of PA with the release of the necine base and necic acid(s). Furthermore, retronecine- and heliotridine-type PAs can undergo *N*-oxidation with the formation of highly water-soluble *N*-oxides which are rapidly excreted in the urine. The activity of metabolic enzymes towards individual PAs plays an important role for the toxicity and varies between species, sexes and at different development stages (COT 2008; Fu et al. 2004; Fu et al. 2002; Huan et al. 1998).

In laboratory experiments, PAs showed the potential to induce genotoxicity in different model systems, and carcinogenicity was observed following chronic oral exposure to several PAs. The main carcinogenicity target for PAs in experimental animals is the liver, although tumours have been also found in other tissues. Today there are no human epidemiological data on PA carcinogenesis (EFSA 2011a; Fu et al. 2004).

Some PAs have been evaluated by the International Agency for Research on Cancer (IARC) (Table 8). According to IARC, for lasiocarpine, riddelliine and monocrotaline the available data gave sufficient evidence for carcinogenicity to experimental animals. Consequently, IARC classified these PAs as being “possibly carcinogenic to humans” (Group 2B) (IARC 1983, 1987, 2002). The experimental results on isatidine, retrorsine, senkirkine were considered as “limited evidence for the carcinogenicity to experimental animals” and these PAs were evaluated as “not classifiable as to its carcinogenicity to humans” (Group 3) (IARC 1983, 1987).

**Table 8:** Evaluation of PAs by IARC

Agent	Animal carcinogenicity data	Evidence for carcinogenicity		IARC-group
		Human	Animals	
Hydroxysenkirkine^a^	Insufficient data	ND	L	3
Isatidine^a^	Carcinogenic in rats: liver tumours (oral administration, limited study)	ND	L	3
Jacobine^a^	Insufficient data	ND	L	3
Lasiocarpine^a^ Lasiocarpine^a^	Carcinogenic in rats: tumours of the liver, skin, intestine (i.p. administration, NTP study)	ND	S	2B
Monocrotaline^a^	Carcinogenic in rats: liver tumours, (oral administration, limited study)	ND	S	2B
Retrorsine^a^	Carcinogenic in rats: tumours in liver and other organs (oral administration, limited study)	ND	L	3
Riddelliine^c^	Carcinogenic in rats and mice: tumours of the liver, respiratory organs (oral administration, NTP study)	ND	S	2B
Seneciphylline^a^	No data	ND	L	3
Senkirkine^b^	Carcinogenic in rats: liver adenomas (i.p. administration, limited study)	ND	L	3
Symphytine^b^	Insufficient data	ND	L	3

*ND* no adequate data, *S* sufficient evidence, *L* limited evidence, *group 2B* “possibly carcinogenic to humans”, *group 3* “unclassifiable as to carcinogenicity in humans”

References: ^a^(IARC 1976); ^b^(IARC 1983); ^c^(IARC 2002)

##### PAs-mediated liver tumour induction in rats

Lasiocarpine and riddelliine are the two PAs with most available data regarding carcinogenicity. Both compounds have been found to induce liver tumours (haemangiosarcomas) in the National Toxicology Program (NTP) chronic tumorigenicity bioassays (NTP 1978, 2003). Other PAs (monocrotaline, senkirkine, symphytine, isatidine and retrorsine) have been also shown to induce tumours in laboratory animals (liver angiosarcomas or adenomas). However, these results were obtained only in non-standard carcinogenicity assays (reviewed in EFSA 2011a).

In the NTP-carcinogenicity study with lasiocarpine, groups of 24 male and 24 female Fischer 344 rats were fed with lasiocarpine doses of 0, 0.35, 0.75 and 1.5 mg/kg bw per day for 104 weeks (NTP 1978). All high dose females had died by week 69, and high dose males had died by week 88, and a dose-related decrease in survival also occurred at the two lower doses. In male rats, the incidence of liver haemangiosarcomas (the most sensitive effect) was 21% (5/24), 48% (11/23) and 56.5% (13/23) in the corresponding three dose groups (low, middle and high). In its assessment from 2011, EFSA used these data on male rats for deriving a BMDL_10_,^1^ the recommended toxicological reference point for calculating the margin of exposure. The derived BMDL_10_ was 0.07 mg/kg bw (EFSA 2011a). No clear dose–response relationship was observed in the incidence of liver haemangiosarcomas in female rats: 36% (8/22), 29% (7/24) and 9% (2/23) incidence in the low, middle and high lasiocarpine dose group, respectively. However, the high early mortality, particularly in female rats, may hinder the adequate detection of the tumorigenic activity.

Riddelliine has been investigated in a two-year carcinogenicity study in F344/N rats and B6C3F1 mice exposed by gavage (5 days/week) for 105 weeks (Chan et al. 2003; NTP 2003). The formation of liver haemangiosarcomas was identified as the key effect. Mice appeared to be less sensitive to riddelliine than rats; and in rats, males were more sensitive than females. Male rats received only one riddelliine dose (1.0 mg/kg bw per day), and liver haemangiosarcomas were found in 43 of 50 animals of this group. The incidence of liver haemangiosarcomas in female rats, receiving riddelliine doses of 0.01, 0.033, 0.1, 0.33 and 1.0 mg/kg bw mg/kg bw per day was 0% (0/50), 0% (0/50), 0% (0/50), 6% (3/50) and 76% (38/50), respectively. Due to the more adequate study design of the riddelliine study and updated guidelines regarding dose–response modelling, EFSA decided in its most recent risk assessment on PAs, to use these data on female rats for deriving the BMDL_10_—instead of the data from the lasiocarpine study. A BMDL_10_ of 0.237 mg/kg bw was derived (EFSA 2017b).

For other PAs (monocrotaline, senkirkine, symphytine, isatidine and retrorsine), positively tested for carcinogenicity in non-standard assays, no dose–response data is available (since only one dose was tested in each case).

##### Genotoxicity and possible mechanisms of tumour formation

The genotoxic mechanisms of PAs include DNA binding as well as DNA-DNA and DNA–protein cross-linking (Table 9). PAs have been shown to be clastogenic and mutagenic (Table10). The metabolic activation of PAs to pyrrolic ester(s) and the subsequent binding to DNA is considered to be the key pathway leading to the genotoxic effects (EFSA 2011a). Several PAs, including monocrotaline, riddelliine, lasiocarpine, clivorine, heliotrine and retrorsine, have been shown to form the same set of DHP-derived DNA adducts either in vivo or in vitro (Fu et al. 2004).

**Table 9:** Studies on reactions of PAs with DNA (based on Chen et al. 2010b; EFSA 2011a; He et al. 2017; Xia et al. 2013).

Agent	DNA adducts in vitro	DNA adducts in vivo	DNA-DNA cross-linking	DNA–protein cross-linking	DNA strand breaks in vitro	UDS in vivo
Clivorine		+				
Heliotrine	+	+				
Heliotrine N-Oxide	+					
Isatidine					+	
Jacobine			+	+	–	
Lasiocarpine	+	+				
Lasiocarpine N-Oxide	+					
Lycopsamin		+				
Monocrotaline	+	+	+	+	±	
Monocrotaline N-oxide	+					
Retrorsine	+	+	+		–	+
Retrorsine N-Oxide	+					
Riddelliine	+	+	+	+	–	+
Riddelliine N-Oxide	+	+				
Senecionine		+	+	+	–	+
Seneciphylline		+	+	+	–	+
Senkirkine		+				

*UDS* unscheduled DNA synthesis. “ + ” positive result; “ –  ” negative result, *empty cells* not tested.

**Table 10:** Clastogenicity and mutagenicity of selected PAs (based on Allemang et al. 2018; Chen et al. 2010b; EFSA 2011a).

Agent	MN in vitro	MN in vivo	CA in vitro	CA in vivo	SCE in vitro	Mutations in bacteria	Mutations in Drosophila
Heliotrine	+	+	+		+	±	+
Isatidine	+		±			–	
Integerrimine		+		+			+
Lasiocarpine	+		+			+	
Monocrotaline	+	+	+		+	–	+
Retrorsine	+		+			+	+
Riddelliine	+	±	+		+	±	
Senecionine						–	+
Seneciphylline		+			+	( +)	+
Senkirkine			+		+	+	+

*MN* micronuclei, *CA* chromosomal aberrations, *SCE* sister chromatid exchange. “  *+ * ” positive result, “ –  ” negative result, *empty cells* not tested

Yang et al. (2001) studied mechanisms of DNA adduct formation by the representative carcinogenic PA riddelliine in female F344 rats. Metabolism of riddelliine by liver microsomes of F344 female rats in the presence of calf thymus DNA resulted in the formation of eight DNA adducts, among which two were enantiomers of DHP-derived 7′-deoxyguanosin-*N*^2^-yl adducts and the other six were DHP-modified dinucleotides. A similar DNA adduct profile was detected in the livers of F344 female rats fed riddelliine for 3 and 6 months. Furthermore, a dose–response relationship was obtained between the dose administrated to the rats (0.01, 0.033, 0.1, 0.33 and 1.0 mg/kg bw) and the level of the riddelliine-induced DNA adducts. Authors concluded, that a genotoxic mechanism, mediated by DNA adduct formation, is involved in the induction of liver tumours in rats fed riddelliine (Yang et al. 2001).

In a mechanistic study by Chou et al. (2004), F344 rats and B6C3F_1_ mice were treated by gavage (5 days per week, for 2 weeks) with 1 and 3 mg/kg bw riddelliine, respectively, and DHP-derived DNA adduct levels were measured in purified DNA from rat and mouse liver parenchymal cells and liver endothelial cells (the cells of origin of haemangiosarcomas). Treatment resulted in significantly greater DHP-derived DNA adduct levels in the endothelial cells than in the parenchymal cells. It was concluded, that the DHP-derived DNA adducts are, at least partially, responsible for the liver tumour development (Chou et al. 2004). In their review on the genotoxicity of PAs, Fu et al. (2004) concluded that the DHP-derived DNA adducts have the potential to be utilized as a biomarker of PA exposure and tumorigenicity.

Unfortunately, DNA adduct formation by lasiocarpine was studied only in vitro (Xia et al. 2006), and no information is available about a possible relationship (correlation) between lasiocarpine-induced DNA adduct formation and tumorigenic responses in vivo. Since lasiocarpine is among the most toxic of the PAs that have been tested, such data would be of high relevance.

PA metabolites (pyrrolic ester(s) and DHP metabolites) contain several reactive sites in their molecule (C5/C7 and C9 positions of the necine base), that are able to bind to two sites of DNA or protein to form DNA or protein cross-linking. Indeed, a number of PAs and their pyrrolic derivatives have been found to form DNA-DNA and DNA–protein cross-links (Table 9) (Coulombe et al. 1999; Kim et al. 1995, 1999). DNA-DNA and DNA–protein cross-links are also discussed as possible mechanisms leading to tumour induction by PAs. Unfortunately, the structures of these cross-links have never been fully characterized; and the levels of their formation have not been correlated with the tumorigenic potencies of rodents treated with PAs (Fu et al. 2004).

PAs are strongly clastogenic agents (Allemang et al. 2018; Chen et al. 2010b; EFSA 2011a; Louisse et al. 2019). Several PAs have been shown to produce micronuclei in vitro with different potency (cultured rat or human hepatocytes) and in vivo (mouse bone marrow erythrocytes and peripheral blood cells). In a structure-dependent way some PAs induce the γH2AX in cell western assay in HepaRG human liver cells in vitro. In addition, PAs induce chromosomal aberrations in mammalian cells or in mouse bone marrow (when they are appropriately metabolically activated). Furthermore, several PAs have been found to induce sister chromatid exchange (SCE) (see Table 10). Altogether, the positive results of clastogenicity assays indicate a likely induction of chromosomal mutations by PAs.

Mutagenicity induced by PAs has been intensively studied in different biological assays, including bacteria (*S. typhimurium*, *E. coli*), *Drosophila*, and rodents. Several PAs were found to be mutagenic in *S. typhimurium* TA100 in the presence of the S9 activation enzyme system (Mattocks 1986; Rubiolo et al. 1992) (see Table 10). In addition, the mutagenicity of riddelliine in rat liver was investigated using Big Blue transgenic rats (Mei et al. 2004a, b). Groups of six female rats were treated by gavage (5 days per week, for 12 weeks) with 0.1, 0.3 and 1.0 mg/kg bw riddelliine. The two top doses have been shown to produce liver tumours in the NTP carcinogenicity study (NTP 2003). Treatment resulted in a significant and almost linear dose-dependent increase in mutant frequencies in the liver *cII* genes. Further investigation in the transgenic rats showed that endothelial cells (the cells of origin of haemangiosarcomas) had a significantly higher *cII* mutation frequency in treated rats compared to control rats, whereas parenchymal cells showed no difference, indicating that mutation by riddelliine is a key event in the carcinogenesis pathway. Moreover, a statistically significant difference was found between the mutational spectra from the riddelliine-treated and control animals. The major types of mutations induced by riddelliine were G:C → T:A transversion and a tandem base substitution of GG → TT and GG → AT. The types of mutations induced by riddelliine were consistent with riddelliine adducts involving G:C base pairs. The GG → TT and GG → AT tandem base substitutions were believed to result from intra-strand cross-links in adjacent guanine bases forming DHP-modified dimers (Chou et al. 2003). In summary, these results show that riddelliine is a genotoxic carcinogen in rat liver and that the types of mutations induced by riddelliine are consistent with riddelliine adducts involving G:C base pairs.

##### Conclusions

Although the carcinogenesis by PAs has long been studied, the mechanisms for the tumour induction in experimental animals by PAs are not completely clear. This is probably due to the structural diversity of PAs. For future, it is necessary to elucidate structure–activity relationships referring to different endpoints to proper assess the risk of PAs for humans and livestock. This includes on the one hand clear data for oral bioavailability of mono-, di- and cyclic diesters at human-relevant doses in dependence of their respective structure. On the other hand, more data for the mode of action, especially in the target organ liver, are needed. Other molecular mechanisms resulting in toxic effects beside the genotoxic-acting properties are under investigation (e.g. Hessel-Pras et al. 2019).

The metabolic activation of PAs to reactive pyrrolic species (DHP and related esters) with the subsequent binding to DNA leads to nucleoside adduct formation, DNA-DNA and DNA–protein cross-linking, which in turn result in gene and chromosomal damage. Both gene and chromosomal mutations are important factors in the induction of tumours by PAs. Furthermore, PAs are considered to be more potent as chromosomal mutagens than as gene mutagens. Since mutations have been found in the target tissues of tumour formation and in oncogenes of PA-induced tumours, it was concluded that PAs induce tumours via a mutagenic mode of action.

There is limited information on dose–response relationships for PA, especially in the low-dose range. In the case of riddelliine, the levels of the DHP-derived DNA adducts correlated closely with tumour formation in rats fed different doses of riddelliine. Moreover, a linear correlation was observed between the administered riddelliine doses and mutation frequencies in liver *cII* genes of Big Blue transgenic rats fed carcinogenic riddelliine concentrations (Chou et al. 2003).

### Carcinogenic metal compounds: Examples cadmium and arsenic

Metals and their compounds are ubiquitously distributed in the environment; however, industrial uses add significantly to human exposure. Many metal compounds are carcinogenic to humans and to experimental animals. This applies not only to toxic metals or semi-metals like cadmium, lead, chromium(VI), arsenic and antimony but also to essential trace elements like nickel and cobalt on conditions of metal overload, exceeding the homeostatic capacity. Nevertheless, with the exception of Cr(VI), most metal compounds are not mutagenic in bacterial test systems and mutagenic responses in mammalian cells are rather weak. Therefore, again with the exception of Cr(VI), direct interactions of metal ions with DNA appear to be of minor importance (Beyersmann and Hartwig 2008). One mechanism frequently proposed to be involved in metal-induced tumour formation is an increase in reactive oxygen species and oxidatively damaged DNA. Furthermore, the interference with the cellular response to DNA damage and with distinct signalling pathways has been identified for many metal compounds during the last years, including interactions with different types of DNA repair systems, cell cycle control and tumour suppressor functions as well as with cell proliferation and cell death (Hartwig 2013b). In addition to interactions with distinct proteins involved in DNA repair, tumor suppressor functions as well as transcription factors, epigenetic mechanisms may play an important role even at low exposure conditions (Arita and Costa 2009). However, especially with respect to occupational and/or environmental exposure limit values, in addition to carcinogenicity, non-cancer organ toxicity has been observed at low concentrations as well, relevant to environmental exposure, as described and discussed for cadmium in detail below. Within the frame of the present work, two examples are particularly relevant, namely cadmium and arsenic.

#### Cadmium

Cadmium is ubiquitously distributed throughout the environment, attributable to natural sources, agriculture and manifold industrial uses. At workplaces, exposure occurs mainly via inhalation, while the general population is exposed predominantly via food, tobacco smoke and ambient air. Even though adverse health effects induced by cadmium have been known for decades, during recent years there has been an ongoing discussion on cadmium-induced toxicity, even at comparatively low exposure conditions. Two lines of toxicity appear to be relevant, the carcinogenicity as well as non-cancer organ toxicity. With respect to the latter, cadmium exposure is associated with bone demineralization, cardiovascular diseases and especially nephrotoxicity as the most sensitive endpoint (Nawrot et al. 2010). Thus, the European Food Safety Authority (EFSA) has lowered the Provisional Tolerable Weekly Intake (PTWI) of 7 µg/kg bw established previously by the Joint FAO/WHO Expert Committee on Food Additives to a TWI of 2.5 µg/kg bw based on cadmium-induced nephrotoxicity (EFSA 2009c, 2011c).

##### Carcinogenicity

Cadmium has been classified and confirmed as human carcinogen by several authorities and scientific committees, including the International Agency for Research on cancer (IARC) and the German MAK Commission (Greim 2006; IARC 2012a). For example, IARC based its decision on sufficient evidence for increased incidences of lung tumours upon inhalation as well as limited evidence for kidney and prostate tumours in humans (IARC 2012a). Furthermore, new data indicate that human cadmium exposure may also be associated with female breast and endometrial cancer, even though the causalities are not definitively established. In experimental animals, cadmium induces carcinomas of the lung after inhalation and cancers of the prostate and testis after ingestion or injection (for review see Hartwig 2013a, 2018) and references therein.

##### DNA damage, mutagenicity and clastogenicity

In most bacterial assays soluble cadmium compounds were not mutagenic. Also, in standard mammalian mutagenicity tests effects of cadmium salts are usually weak and/or restricted to comparatively high concentrations. In contrast, pronounced co-mutagenic effects in combination with DNA alkylating agents and with UVC radiation were observed both in bacteria and in mammalian cells. In addition, in mammalian cells cadmium compounds provoke clastogenic effects such as chromosomal aberrations and micronuclei (Filipic et al. 2006; Greim 2006; Hartwig 2010; IARC 2012a). The clastogenic activity of cadmium compounds is moreover evident in vivo in exposed rodents, while evidence for chromosomal damage in cadmium-exposed humans via environmental or workplace exposure is equivocal, partly due to simultaneous exposure to other metal compounds (Greim 2006; IARC 2012a; Verougstraete et al. 2002). Furthermore, cadmium compounds increase the extent of oxidative DNA damage in cultured cells and in vivo*,* albeit by indirect mechanisms (see below).

##### Mechanisms in cadmium-induced carcinogenicity

Since cadmium salts do not cause DNA damage in cell extracts or with isolated DNA (Valverde et al. 2001) but rather interact with proteins, the genotoxicity of cadmium is likely due to indirect mechanisms. Multiple indirect mechanisms appear to be relevant, (1) the increased formation of reactive oxygen species, (2) interactions with the cellular DNA damage response system, such as DNA repair processes, cell cycle control and apoptosis as well as (3) epigenetic changes in DNA methylation patterns, leading to a high degree of genomic instability (Arita and Costa 2009; Beyersmann and Hartwig 2008; Fischer et al. 2016; Hartwig 2013a; Hartwig 2013b; Hartwig 2018; Joseph 2009; Templeton and Liu 2018).

*Oxidative stress and oxidatively* induced *DNA lesions.* Even though cadmium(II) is not able to participate in redox reactions under physiological conditions, increased levels of reactive oxygen species (ROS) have been observed both in vitro and in vivo (Liu et al. 2009), and their appearance is interpreted by the inhibitory effect of cadmium on antioxidant enzymes such as catalase, superoxide dismutase, glutathione reductase, and glutathione peroxidase (Stohs et al. 2001; Valko et al. 2006). Furthermore, different cadmium compounds have been shown to induce DNA strand breaks and oxidatively induced DNA base modifications in mammalian cells, but effects were usually small and/or restricted to comparatively high concentrations (e.g., Dally and Hartwig 1997; Schwerdtle et al. 2010) and thus do not appear to be sufficient to explain the carcinogenicity of cadmium, and enhanced frequencies of oxidative DNA lesions in cells and in vivo may also be due to impaired removal of the respective lesions (see below).

*Interactions with DNA repair and tumour suppressor functions.* Cadmium has been shown to impair almost all major DNA repair pathways. Multiple evidence is available for its interference with nucleotide excision repair, base excision repair and mismatch repair, providing a plausible explanation for its co-mutagenicity in combination with different DNA damaging agents, including UVC radiation and DNA alkylation agents (reviewed in Hartwig 2013a). With respect to NER, impaired removal of UVC- and benzo[*a*]pyrene-induced DNA damage has been demonstrated in cell culture systems, due to an impaired assembly/disassembly of the DNA damage recognition proteins XPC and XPA at the repair complex after UVC irradiation in cells (Schwerdtle et al. 2010). Regarding BER, low concentrations of cadmium inhibited the repair of oxidative DNA base damage as well as DNA alkylation damage in mammalian cells (Dally and Hartwig 1997; Fatur et al. 2003). When compared with the induction of oxidatively induced DNA base modifications such as 8-oxoguanine (8-oxoG), inhibitory effects on the repair of this lesion were observed at much lower cadmium concentrations. This has been observed by direct comparison in HeLa cells: While the induction of DNA strand breaks by cadmium was restricted to 10 µM and higher, the removal of oxidative DNA base modifications induced by visible light and recognized by the bacterial formamidopyrimidine DNA glycosylase (FPG) was inhibited starting at 0.5 µM cadmium, yielding complete inhibition at 5 µM, a completely non-cytotoxic concentration in this test system (Dally and Hartwig 1997). Inhibitory effects of enzymes involved in the defence against oxidative DNA damage are also evident in vivo: When investigating, for example, the impact of cadmium on rat testis, a target organ for cadmium carcinogenesis, a gradual decrease in testicular 8-oxo-dGTPase activity was observed, accompanied with the progressive increase of 8-oxo-dG levels in testicular DNA (Bialkowski et al. 1999). Therefore, increases in oxidative DNA damage in vivo may at least in part be due to the repair inhibition of endogenously induced oxidative DNA lesions. In addition to excision repair, cadmium has been shown to impair DNA mismatch repair in different systems. It was first reported by Jin and co-workers that exposure towards low concentrations of cadmium resulted in pronounced hypermutability in yeast, and the mutation specificity along with responses in proofreading-deficient and MMR-deficient mutants indicated a reduced capacity for MMR of small misalignments and base–base mismatches upon cadmium exposure. Furthermore, in extracts of human cells, cadmium inhibited at least one step leading to mismatch repair (Jin et al. 2003). Since then, different studies demonstrated the interference by cadmium with proteins involved in the initial step of MMR, i.e. damage recognition by MSH2-MSH6 and MSH2-MSH3. Even though the exact mechanism is still not known at present, cadmium affected ATP binding and hydrolysis of MMR enzymes, reducing their DNA binding activity and their ability to discriminate between mismatched and matched DNA base pairing in isolated systems and in mammalian cells in culture (for review see Hartwig 2010). In addition to manifold interference with DNA repair systems, cadmium has also been shown to disturb the function of the tumour suppressor protein p53 and thus interferes with cell cycle control in response to DNA damage (Méplan et al. 1999; Schwerdtle et al. 2010). Furthermore, cadmium-induced malignant transformation of human prostate epithelial cells acquired resistance to apoptosis (Qu et al. 2007). As a consequence, damaged cells could escape from elimination by apoptosis, allowing them to replicate damaged DNA with a high frequency of mutations, which may play an important role in cadmium-induced carcinogenicity (see below).

*Impact on gene expression and deregulation of cell proliferation.* Cadmium interacts with the expression of a large number of genes, including stress response genes, immediate early response genes, transcription factors and translation factors. Major stress response genes induced by cadmium are those involved in the synthesis of metallothionein (MT), as well as those encoding heat shock proteins, glutathione (GSH) synthesis and homeostasis and oxidative stress response (Fischer et al. 2016; Joseph 2009; Waisberg et al. 2003). Immediate early response genes induced by cadmium include proto-oncogenes like *c-fos*, *c-jun* and *c-myc* activated in response to mitotic stimuli and frequently found overexpressed in tumours. In a recent study, the impact of cadmium on the transcriptional oxidative stress and DNA response was investigated via a quantitative high-throughput PCR approach. Cadmium activated genes coding for the stress response, anti-oxidative defence, mitotic signalling and cell cycle control as well as the intrinsic apoptotic pathway. With respect to DNA damage induction and repair, it induced damage response genes like *GADD45*, but down-regulated genes coding for specific DNA repair proteins involved in all major DNA repair pathways (Fischer et al. 2016). All these interactions mirror the manifold interactions of cadmium supposed to be involved in cadmium-induced carcinogenicity. With respect to transcription factors, cadmium exposure may lead to activation or inactivation, depending on the actual transcription factor under investigation.

*Molecular mechanisms.* Independent of the actual cadmium compound applied, Cd^2+^ appears to be the ultimate damaging species, and similar interactions have been observed in case of water soluble and particulate cadmium compounds. Repair inhibitory effects were strongly correlated with cadmium levels in the nuclei (Schwerdtle et al. 2010). Since cadmium ions exert high affinity towards SH groups, potential targets are zinc-binding proteins (Hartwig 2001; Witkiewicz-Kucharczyk and Bal 2006). They comprise a family of proteins where zinc is complexed through four invariant cysteine and/or histidine residues forming a zinc finger domain, which is mostly involved in DNA binding, but also in protein–protein-interactions. Even though most zinc finger structures have been described as DNA binding motifs in transcription factors, they have also been identified in several DNA repair proteins required for basically all major DNA repair pathways as well as the tumour suppressor protein p53 and the DNA damage signalling enzyme poly(ADP)polymerase 1 (PARP1). Many of these proteins have been shown to be inhibited by cadmium in different experimental systems, presumably via displacement of zinc by cadmium, as evident from structural investigations elucidating interactions with the nucleotide excision repair protein XPA or a peptide resembling the zinc-binding domain of XPA (reviewed in Hartwig 2010). Additionally, cadmium ions may interfere with thiols in cysteines in critical positions outside zinc-binding structures as well; these structures are frequently redox-regulated for example in transcription factors. In addition, especially moderately elevated levels of ROS have been implicated in cell proliferation due to mitotic stimuli and the activation of redox-sensitive transcription factors (Valko et al. 2006). Therefore, enhanced oxidative stress induced by cadmium may deregulate cell growth and promote tumour growth depending on dose and time of interference. Altogether, an interference with redox-regulation of cell growth may be one unifying mechanism in metal-induced carcinogenicity (Hartwig 2013b). Finally, cadmium also exerts epigenetic effects, evident by inhibition of DNA-(cytosine-5) methyltransferase and disturbed DNA methylation patterns during cadmium-induced cellular transformation (Arita and Costa 2009; Brocato and Costa 2013; Takiguchi et al. 2003). In conclusion, it is evident that cadmium interferes with cellular controls of proliferation in several ways, which all can contribute to the observed deregulation of cell growth by this metal.

#### Arsenic

Arsenic is a semi-metal and occurs in the oxidation states + 5, + 3, 0 and –3 in organic and inorganic species. Both natural and anthropogenic sources are relevant. Depending on geological conditions, drinking water can be a significant source of exposure to arsenic. Here, arsenic is mostly present in inorganic form as arsenate (+ 5), under reducing conditions also as arsenite (+ 3). The concentration of arsenic in the groundwater is usually less than 10 μg/L, but in some areas of the world, as in India or Bangladesh, concentrations of more than 3000 μg/L are reached. Other significant sources towards inorganic arsenic are rice and rice products, which contain concentrations of 0.1–0.4 mg/kg dry mass and sometimes clearly above. Even higher amounts of dietary intake arise from the consumption of fish and seafood, where arsenic is predominantly found in organic form as arsenobetaine in average concentrations of 0.1–1.8 mg arsenic/kg. In algae, arsenic is predominantly present as arsenosugars, the concentrations usually being in the range of 2–50 mg arsenic/kg dry mass Occupational exposure to arsenic occurs in metal production and processing. Arsenic and arsenic compounds are used in semiconductors as gallium arsenide, in wood preservatives and in alloys. In the past, arsenic-containing pesticides added further to human exposure. Another anthropogenic source for the release of arsenic into the environment is the burning of fossil fuels. From a toxicological point of view, especially inorganic arsenic such as arsenate (+ 5) and arsenite (+ 3) exert adverse health effects, while organic arsenic appears to be less toxic. Inorganic arsenic compounds, especially arsenic trioxide (As_2_O_3_), are famous as poison in many murder cases. While 0.1 g arsenic trioxide by the oral route is already fatal, small doses of 2 mg taken daily by so-called arsenic eaters were claimed to have a postulated performance-boosting effect and to protect from poisoning; from today's point of view, however, clearly chronic toxicity including carcinogenicity is associated with these intake levels. The acute toxic effects of arsenic and arsenic compounds include gastrointestinal, cardiovascular, renal and neurotoxic effects. Trivalent inorganic arsenic compounds are usually more toxic than the pentavalent compounds. Organic arsenic compounds usually have a markedly lower acute toxicity. Today, more attention is paid to the chronic effects of arsenic. Besides exposure at the workplace, the chronic uptake of inorganic arsenic compounds with the drinking water and–especially for infants and children–rice and rice products can lead to toxic effects. These include skin changes and blood flow disorders (“blackfoot disease”), cardiovascular diseases, neurotoxicity as well as developmental toxic effects, but also carcinogenicity at particularly low concentrations (for reviews see (EFSA 2014a; Greim 2005; Hartwig and MAK Commission 2016; IARC 2012a).

##### Carcinogenicity

As for arsenic in general, also for carcinogenicity exposure towards inorganic arsenic is most relevant. After inhalation or ingestion, humans and many other mammals metabolize inorganic arsenic into organic forms. After reduction of arsenate, arsenite is metabolized to trivalent and pentavalent methylated species, namely monomethylarsonous (MMA(III)) and dimethylarsinous (DMA(III)) acid, monomethylarsonic (MMA(V)) and dimethylarsinic (DMA(V)) acid, and especially the trivalent methylated species contribute to arsenic-induced genotoxicity and presumably carcinogenicity. Epidemiological studies provide reliable evidence for an increased incidence of lung tumours after inhalative and oral exposure towards inorganic arsenic. Thus, arsenic and its inorganic compounds have been classified as carcinogenic in humans by the IARC (Group 1) (IARC 2012a) and the MAK Commission (carcinogenicity category 1) (Greim 2006). A drinking water limit of 10 μg arsenic/L was established by the World Health Organization (WHO) and the US Environmental Protection Agency (EPA). For oral uptake of inorganic arsenic via foodstuff, the European Food Safety Authority (EFSA) has identified a lower confidence limit for an additional risk for lung, skin and urinary bladder cancer of 1% (BMDL_01_) of 0.3 to 8 μg/kg bw and day based on dose–response relationships derived from epidemiological studies, by means of benchmark calculations. Since even in Europe the estimated average dietary exposure of the general population to inorganic arsenic is within this range, a possible cancer risk for consumers cannot be excluded and a TDI value could not be derived (EFSA 2014a).

##### DNA damage, mutagenicity and clastogenicity

Arsenite does not induce point mutations in bacterial or mammalian test systems. However, it increases the mutagenicity of other DNA damaging agents, such as UVC radiation. In contrast, the induction of micronuclei, chromosomal aberrations, DNA strand breaks and oxidative DNA base damage is well documented and has been observed at comparatively low concentrations in cultured mammalian cell lines such as V79, CHO, A549, in human peripheral lymphocytes, buccal and bladder cells after exposure to arsenite via drinking water as well as in mice after oral exposure to comparatively low concentrations of arsenite. With respect to the inorganic species, arsenate (with pentavalent As) and arsenite (with trivalent As), similar genotoxic effects have been observed, albeit at about tenfold higher concentrations of arsenate as compared to arsenite. Regarding the methylated species, MMA(III) and DMA(III) are genotoxic at lower concentrations than arsenite at all endpoints, while genotoxic effects of MMA(V) and DMA(V) are either absent or restricted to much higher concentrations (for review see Beyersmann and Hartwig 2008). As underlying mechanisms for co-mutagenicity and clastogenicity, interactions with DNA repair mechanisms and other DNA damage response systems appear to be most relevant.

*Oxidative stress and oxidatively induced DNA lesions.* Oxidative stress and thus elevated levels of ROS and RNS is thought to be an important mechanism in arsenic-induced carcinogenicity. Underlying mechanisms may be manifold and include their generation during metabolism, interactions with the respiratory chain, the release of iron from ferritin and modulation of NO synthases. Moreover, arsenicals have been shown to interfere with cellular redox homeostasis by decreasing the cellular GSH content, either by complexing thiol groups, resulting in GSH binding and depletion, consumption of GSH during arsenic metabolism, or by interactions with glutathione-related enzymes (Hartwig 2013b; Thomas 2007; Valko et al. 2006 and references therein).

*Interactions with DNA repair and tumour suppressor functions.* Similar to cadmium, one important mechanism in arsenic-induced carcinogenicity appears to be the interaction with DNA repair systems. Trivalent but not pentavalent arsenicals have also been shown to inhibit NER at low, non-cytotoxic concentrations. In this context, the removal of UVC- and benzo[*a*]pyrene diol epoxide-induced DNA lesions was impaired by arsenite and even more pronounced by MMA(III) and DMA(III) in cultured cells and laboratory animals. In addition, arsenite and its methylated metabolites inhibited BER, evident for example in diminished hOGG function (for review see Beyersmann and Hartwig 2008; Hartwig and Schwerdtle 2009 and references therein). The most sensitive target related to DNA repair affected by trivalent arsenicals, however, is poly(ADP-ribosyl)ation. Thus, low nanomolar concentrations of arsenite, MMA(III) and DMA(III) inhibited poly(ADP-ribosyl)ation in human HeLa cells, while the pentavalent species monomethylarsonic (MMA(V)) and dimethylarsinic (DMA(V)) were not inhibitory. Furthermore, all three trivalent arsenicals inhibited the isolated PARP1, indicating a direct interaction with this enzyme (Hartwig et al. 2003; Walter et al. 2007; Zhou et al. 2011).

*Impact on gene expression and deregulation of cell proliferation.* Arsenite has also been shown to activate several redox-regulated signalling pathways, including all three classes of mitogen-activated protein kinases (MAPKs). Thus, for example, in a mesencephalic cell line arsenite at low, non-cytotoxic concentrations activated NF-κB and AP-1 and induced phosphorylation of ERK1/2. One other transcription factor activated by arsenite is Nrf2, leading to the expression of antioxidant enzymes via ARE-responsive elements (for reviews see Druwe and Vaillancourt 2010; Hartwig 2013b; Kumagai and Sumi 2007).

Published data concerning the impact of arsenite on the tumour suppressor protein p53 are controversial and seem to depend on cell line and incubation time. Thus, arsenite-induced accumulation of p53 in human fibroblasts and a human lymphoblastoid cell line via an ATM-dependent pathway (Menendez et al. 2001; Yih and Lee 2000). On the other hand, p53 function has been shown to be inactivated by arsenite and MMA(III). Thus, MMA(III) led to a marked impairment of p53 induction in response to benzo[*a*]pyrene diol-epoxide and reduced p53 DNA binding, presumably involved in arsenical-induced NER inhibition (Nollen et al. 2009; Shen et al. 2008). This may be due to the unfolding of the zinc-binding domain of p53, yielding the so-called mutant conformation, as has been shown after treatment of human SV-40 immortalized uroepithelial cells after treatment with arsenite (Chai et al. 2007). Interestingly, after long-term exposure of human skin keratinocytes an inactivation of p53 was found to be mediated via poly(ADP-ribosyl)ation of p53, in spite of a globally reduced level of poly(ADP-ribosyl)ation (Komissarova and Rossman 2010).

With regard to changes in gene expression, one other mechanism consists in epigenetic alterations provoked by arsenite, both in cellular systems as well as in exposed humans, leading to both hypomethylation and hypermethylation, with the consequence of the activation of oncogene expression and silencing of tumour suppressor genes. One underlying mechanism appears to be an inactivation of DNA methyltransferases; however, since hyper- and hypomethylations are observed, interactions appear to be complex and are currently not completely understood (for review see Brocato and Costa 2013; Reichard and Puga 2010).

*Molecular mechanisms.* In general, trivalent arsenicals such as arsenite and MMA(III) exert higher affinities for dithiol or trithiol sites in proteins as compared to monothiol sites. Therefore, zinc-binding structures in DNA repair proteins, in the tumour suppressor protein p53 as well as in transcription factors may be particularly sensitive targets. Structural investigations further revealed a selective interaction with zinc-binding structures containing three or four cysteine residues (Zhou et al. 2011). This may explain the inactivation of DNA repair systems at very low concentrations. Thus, for example, poly(ADP-ribose)polymerase I contains three zinc-binding structures involved in DNA damage recognition and interactions with further DNA repair proteins. Recent evidence suggests that especially zinc finger I may not be saturated with zinc on normal cellular conditions, rendering it potentially very sensitive towards arsenite (Bossak et al. 2015). Also with regard to the inhibition of DNA repair systems interactions with zinc-binding NER proteins may be plausible. Thus, trivalent but not pentavalent arsenicals have been shown to release zinc from a 37 amino acid peptide resembling the zinc finger domain of the human XPA protein (XPAzf) (Schwerdtle et al. 2003), albeit by different mechanisms. While equimolar concentrations of MMA(III) mediated zinc release, forming mono- and diarsenical derivatives of XPAzf, a tenfold excess of arsenite was required to partially oxidize XPAzf, yielding one or two disulfide bonds (Piatek et al. 2008). With regard to the inhibition of NER in cells, arsenite and—again at even lower concentrations—MMA(III) were shown to inhibit the association of the damage recognition protein XPC to the site of UVC-induced DNA damage. Along with diminished gene expression of XPC and XPE and a reduced XPC protein level, this may be explained by reduced p53 activity, perhaps due to unfolding of the zinc-binding structure within the DNA binding domain described above. Regarding interference with signal transduction pathways, reactions with critical cysteine residues and thus interference with redox regulation of transcription factors appear to be the dominant mechanism, demonstrated for example for the Keap1-Nrf2 system (reviewed in Hartwig 2013b). In addition, also epigenetic mechanisms appear to be relevant (Brocato and Costa 2013; Reichard and Puga 2010), but molecular mechanisms remain to be elucidated.

#### Conclusions and in vivo relevance

In the case of cadmium and arsenic, direct interactions with DNA appear to be not relevant in the low-dose range, also supported by missing direct mutagenicity. Nevertheless, the frequency of mutations may be elevated by indirect mechanisms, such as via interference with basically all major DNA repair systems. Since DNA damage is not only induced by exogenous mutagens but also continuously due to endogenous processes as described above, this may even lead to pronounced hypermutability of exposed cells. In addition, further targets have been identified with relevance for genomic stability, such as an inhibition of anti-oxidative defense systems, an inactivation of tumor suppressor functions and altered signal transduction processes. While in some cases distinct proteins have been identified as molecular targets, also epigenetic mechanisms appear to be relevant. All these features taken alone could contribute to carcinogenicity, but most likely their combination seems to be of particular importance. Thus, for example, long-term exposure to low concentrations of cadmium leads to adapted cells exerting increased cadmium accumulation, increased proliferation, diminished DNA repair and cell cycle control as well as resistance to apoptosis (reviewed in Hartwig 2018). In principle, all the above-mentioned mechanisms are mediated via interactions with proteins, and a threshold is likely. However, for both metals, there are only limited in vivo data available which allow a clear distinction of mechanisms relevant or irrelevant of exposed humans, but some interactions have been observed at particularly low concentrations, which are found in either environmentally or occupationally exposed humans.

In case of cadmium, levels in blood and urine are usually low, in the nanomolar concentration range (Borjesson et al. 1997), and thus considerably lower when compared with, for example, DNA repair inhibitory concentrations in cell culture systems in the low micromolar concentration range. However, far higher concentrations are found in organs like liver and kidney, reaching–based on the wet weight in the respective organs–up to 90 µM in the liver and up to 0.5 mM in the kidney, already in the general population not additionally exposed at the workplace (Lech and Sadlik 2017). Even millimolar concentrations have been reported in the kidney cortex as well as high micromolar concentrations in the liver of cadmium-exposed workers (Borjesson et al. 1997). However, in these organs, exposure-related induction of metallthioneins (MT) a family of small cysteine-rich proteins occurs. MTs exert high binding affinity to metal ions such as cadmium via SH groups, thereby achieving effective cellular protection. Nevertheless, while clearly protecting from acute toxicity, binding of cadmium ions to MTs in vivo leads to very long half-lives of up to several decades. This is most apparent in the case of cadmium accumulation and retention in the kidney, as evident from MT-knockout mice (reviewed in Klaassen et al. 2009). As a consequence, MT-mediated adaptation may lead to reduced DNA repair activities as well as suppressed apoptosis (Hart et al. 2001; Singh et al. 2009a). Even though cadmium binding to MT is thermodynamically very stable, toxic metal ions may be released, for example under conditions of oxidative stress, thereby becoming available for interaction with critical cellular structures (for review see e.g., Namdarghanbari et al. 2011). Of note, indications for DNA repair inhibition were observed in lymphocytes of cadmium-exposed workers (Hengstler et al. 2003). Thus, research is needed on cadmium levels that may lead to cellular interactions leading to genomic instability in exposed humans.

However, with regard to limit values for cadmium exposure, there is clear evidence that non-cancer effects in humans may be relevant at even lower concentrations. Thus, as evaluated by EFSA and detailed above, nephrotoxicity was selected as the most sensitive endpoint to determine the tolerable weekly intake (TWI) of 2.5 µg Cd per kg body weight for cadmium. This value is almost within daily uptake levels, even without additional workplace exposure and may be exceeded by some population groups with high food intake (EFSA 2009c, 2011c). In consequence, in the case of oral cadmium exposure, kidney toxicity appears most relevant, occurring within an exposure range close to environmental exposure. Thus, exposure levels protecting from kidney toxicity upon oral exposure as well as from lung toxicity after inhalation presumably also protect from carcinogenicity.

In case of arsenite, the situation is different. Here, relevant cancer risks appear to be associated already with environmental exposure levels (EFSA 2014a). Thus, as detailed above, for oral uptake, EFSA has identified an additional risk for lung, skin and urinary bladder cancer of 1% (BMDL_01_) for uptake levels of 0.3 to 8 μg/kg bw and day which is within the estimated average dietary exposure of the general population. Therefore, an elevated cancer risk for consumers cannot be excluded and a TDI value could not be derived (EFSA 2014a). Levels of inorganic arsenic in urine of the general population are around 6.7 nM (Ochsmann et al. 2018). Therefore, on the molecular level, especially those interactions observed at particularly low, nanomolar or even sub-nanomolar concentrations in cellular test systems will likely be relevant also in vivo. This includes an interference with poly(ADP-ribosyl)ation involved in DNA repair, cell cycle control and apoptosis observed at sub-nanomolar concentrations. In addition, also epigenetic alterations leading to gene expression changes appear to be relevant at very low concentrations (e.g. States 2017). Thus, for risk assessment of arsenite, even though a threshold-like mechanism has been demonstrated, it may not be possible to define health-based exposure levels protecting from respective interactions and thus from carcinogenicity, or, if so, they may be expected to be below present environmental exposure levels; again, research is still needed to clarify this aspect.

## Part C: Conclusions and Perspectives

### General considerations

In general, toxicological cancer risk assessment is intended to quantitatively link exposure levels (or doses) with the associated cancer risk or incidence. The risk assessment of carcinogenic substances in food and at the workplace is an issue of ongoing scientific scrutiny since the exposure, in particular at trace levels, to these compounds is not completely avoidable.

The current risk assessment paradigm relies on the differentiation between genotoxic and non-genotoxic carcinogens. For non-genotoxic carcinogens, which are often classified as “promotors”, no observed adverse effect levels (NOAELs) are accepted to exist, and threshold values are frequently proposed. In contrast, genotoxic agents, their metabolic precursors and DNA reactive metabolites are assumed to represent risk factors at all concentrations because in principle even one or a few DNA lesions may result in mutations and thus increase tumour risk. Frequently, a linear extrapolation from the high dose range (for example resulting in 25% tumour risk in animal studies) down to low dose levels is applied to assess the cancer risk under low exposure conditions. For most substances, however, this may result in a significant overestimation of the low dose risk, since tumour formation by genotoxic carcinogens is often drastically enhanced in the high dose range because: (1) defense mechanisms become saturated (2) various types of promotional mechanisms are activated. Examples include regenerative growth due to cytotoxic effects of the agents (for details, see part A "Fundamental considerations", chapter "DNA reactivity of chemicals: Hazard versus Risk"and "Test systems in genetic toxicology"). This gives rise to a biphasic shape of the dose–response curve (part A "Fundamental considerations", Fig. 1), with the implication that under realistic exposure conditions only the slope of the first (low dose) range in most cases is relevant for the risk assessment (part A "Fundamental considerations", chapter “Introduction”). Moreover, some agents exert their genotoxicity via indirect interactions, such as interference with the cellular response to DNA damage including DNA repair processes, cell cycle control and tumour suppressor functions, giving rise to non-linear dose–response relationships.

As a consequence, for genotoxic carcinogens, a mechanism-driven risk assessment is proposed that considers key events leading to the apical outcomes, mutation and malignant transformation. Every single key event is expected to follow its own (maybe non-linear) dose dependence and kinetics.

#### Identification of the mode of action (MoA) of chemical carcinogens

In general, combinations of genotoxicity tests are used to evaluate the genotoxic potential of a compound, with the aim of covering all possible mechanisms that can lead to tumour initiation. In addition to rather “classical” test systems for mutagenicity and clastogenicity (e.g., Ames, HPRT, mouse lymphoma, chromosomal aberrations or micronucleus tests), novel test systems, which provide more comprehensive mechanistic information on key events and toxicological “fingerprints” leading to genotoxicity and/or mutagenicity in vitro and in vivo*,* are under development (see part A "Fundamental considerations", chapter "Protein adducts as human biomarkers"). Furthermore, mechanistic studies are required to discriminate between direct and indirect genotoxicity.

The induction of genomic damage (such as DNA and chromosomal lesions or chromosomal mutations) is the initial event in a chain of steps that eventually may lead to the formation of a tumour. The ability to exactly identify and quantify DNA lesions in tissues and body fluids has remarkably increased in recent years (see part A "Fundamental considerations", chapter "Toxicogenomics for hazard identification and risk assessment"). Reliable dosimetry of DNA damage associated with exposure to minute traces of genotoxic contaminants in food and other consumer media can be achieved with present day advanced instrumental analysis. Of note, DNA is also continuously damaged by endogenous processes, in which reactive oxygen species (ROS) and other electrophiles are generated, in part due to leakage from the electron transport chain operative in cellular respiration and to electrophile leakage from physiological metabolism but also in part by regular metabolic intermediates such as formaldehyde, acetaldehyde and ethylene oxide (see also part B “Selected examples”, chapter “Presence of endogenous background levels of the same or similar DNA lesions”). This leads to measurable steady-state levels of different types of DNA lesions, such as oxidized bases and alkylation products, in apparently all types of cells and tissues (see part A “Fundamental considerations”, chapter "Background DNA lesions in rodent and human tissues/body fluids"). In exposed humans, several cellular defence mechanisms and scenarios may counteract or even prevent the induction of significant levels of DNA damage. It is, therefore, of crucial importance to measure steady-state levels of DNA damage in vivo*,* which would account for both, detoxification before the compound or its reactive metabolite reaches the DNA, and for DNA repair. If no increase in steady-state levels of “background” DNA damage or no increase in endogenously induced levels of the respective DNA lesions is observed, no increase in mutations and thus no elevated risk for malignant transformation would be expected at this exposure level. In practice, however, one has to consider the statistical power of the respective measurements to quantify remaining risks. If human exposure is well (in practice often orders of magnitude) below the levels at which, for example, an appreciable increase in the background frequency of the respective DNA lesions can be statistically excluded, genotoxicity at this exposure level may be judged irrelevant and, thus, of low priority for further consideration of carcinogenicity. Moreover, in the case of very reactive chemicals such as acetaldehyde or formaldehyde (chapters "Acetaldehyde and Ethanol" and "Formaldehyde"), one needs to consider that not only the background levels of the compounds in blood but also their local levels at the site of entry, for example, nasal tissue in case of inhalation or levels reached in the gastrointestinal tract in case of oral exposure, might be relevant.

In contrast to DNA lesions, which may be repaired, mutations are irreversible changes in the nucleotide sequence and thus in genetic information, which, depending on the affected position or gene, might be of considerable relevance for the carcinogenicity process. Therefore, it would be highly desirable to measure mutations instead of DNA damage. Nevertheless, this would require very sensitive in vivo mutagenicity test systems, which are currently not available. One promising approach could be the newly established PIG-A test, which could even be implemented in repeated-dose toxicity studies, since only sampling of a small amount of blood is required for this test (for a recent review see Olsen et al. 2017).

Genotoxic compounds leading to DNA lesions may be clustered together with respect to the type of DNA damage they induce. Provided basal levels of the respective lesions in human tissues/cells are known, the additional DNA modifications caused by the exposure to a given xenobiotic compound can be used to evaluate the incremental increase of risk (relative additional risk) associated with this exposure. A key prerequisite for such a risk assessment approach is to know the type and frequency of biological effects, especially mutations and malignant cell transformation processes, possibly occurring after the respective DNA lesions have been induced. The kinetics of induction and disappearance of DNA lesions, e.g. by damage repair, need specific consideration with regard to mutation fixation. In addition, the increase in induced mutations may follow quite different dose–response relationships. At higher concentrations (part A "Fundamental considerations", Fig. 1, range B), additional important effects need to be taken into account, especially proliferative responses and enhanced cell division within target cells/tissues.

In addition to the dosimetry of DNA lesions, monitoring of blood protein adducts provides complementary information. This relates especially to the internal exposure towards electrophilic compounds resulting from a defined exogenous exposure, e.g. at specific working places. Techniques to assess the internal exposure via quantification of protein adducts are well established for haemoglobin (Hb) and serum albumin (SA) as preferred targets (dosimetry) (see part A "Fundamental considerations"; chapter "Toxicogenomics for hazard identification and risk assessment"). It has to be kept in mind, however, that protein adducts are to be considered markers of exposure, since, in contrast to DNA lesions, they are not repaired and, thus, indicate elevated exposure, usually without relevance for the carcinogenic process as such. To monitor short term exposures that may not cause measurable changes of adduct levels in blood proteins, the dosimetry of mercapturic acids (MA) or other metabolites in urine appears the method of choice (see part B "Selected examples", chapter "Acrylamide"). Such biomonitoring results can be assessed using reference parameters, e.g. the BAR value (Biological Reference Value) of the German DFG MAK Commission. The BAR value is based on the respective biomarkers reflecting background exposure of an occupationally unexposed reference population at working age. In perspective, protein adducts and/or urinary/body fluid biomarkers (e.g. MA) may complement or even replace the determination of external exposure to dietary and environmental genotoxins.

More comprehensive mechanistic information can be provided by toxicogenomics approaches. Appropriate toxicogenomics data have to be linked, also in terms of dose–response behaviour, to toxicologically relevant traditional apical endpoints. If properly used, such studies can provide information to improve our mechanistic understanding of low dose relationships and, in consequence, of “biological thresholds”. However, it has to be kept in mind that the onset of transcriptional responses may require a certain level of DNA damage, which may be converted into mutations even at lower concentrations (see part B "Selected examples", chapter "Benzo[a]pyrene"), which can restrict the sensitivity of such approaches. In addition, it becomes apparent that wherever available, patterns or signatures of biomolecules should be used to elucidate modes of action and to identify key events of relevance for adverse outcome pathways (see part A "Fundamental considerations", chapter "Background DNA lesions in rodent and human tissues/body fluids").

In combination with physiologically based kinetics (PBK) unique possibilities are provided to build up models reflecting the human situation, and even to take into account inter-individual differences. Furthermore, responses under realistic low dose regimens can be predicted. This may inform about the dose–response relationship of DNA reactive metabolites supposedly operative at dose levels that are experimentally not accessible (see part B "Selected examples", chapter "Allylalkoxybenzenes").

Structure Activity Relationship (SAR) involves in silico methods designed to find relationships between the chemical structure and the biological activity of compounds. Such novel in silico approaches may support a read-across from compounds for which in vivo studies are available to those with limited or completely absent toxicity data.

The mechanistic profile resulting from the above information is expected to comprehensively inform about adverse outcome pathways relevant for improved risk assessment.

#### Distinction of carcinogens based on MoA and quantitative risk assessment

**Fig. 20:**
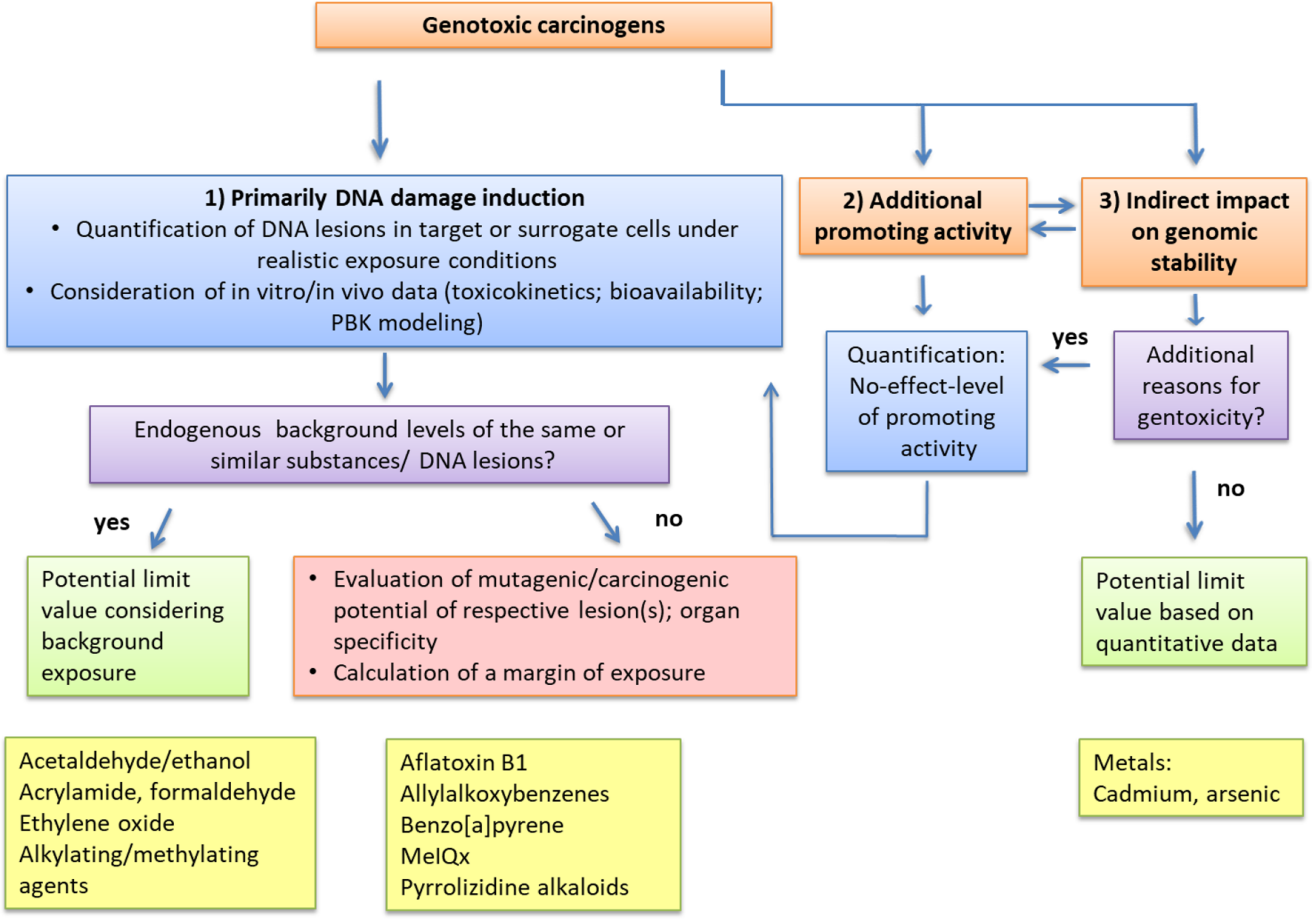
A Strategy for the assessment of the carcinogenic risk associated with an exposure to examples of genotoxic compounds. For details see text

Based on such an assessment of the key events leading to the apical outcome, i.e. the malignant cell transformation, strategies may become applicable in the near future that allows to further characterize the mode of action of genotoxic carcinogens and to specify virtually safe doses at least in some cases. An example of this type of strategy is outlined in Fig. 20 and described below.

The first step in the strategy outlined in Fig. 20, therefore, is the mode-of-action based mechanistic characterization of genotoxic compounds. At the doses at which an increased cancer incidence is observed they may act primarily by DNA damage induction (1), they may additionally exert promoting activity (2) or they may (possibly additionally) affect the processing of DNA modifications (3). For the discrimination of the three groups in vitro data provide valuable hints on the mode of action, including dose–response relationships with regard to DNA damage induction and mutagenicity. For substances with genotoxic and additional promotional activity (Group 2), risk assessment would largely profit from the reliable quantification of cancer risk at concentrations below the onset of promotional activity, i.e. the border between range A and range B in Fig. 1 (Part "Fundamental considerations", “Introduction”). However, in most cases tumour incidences in this range will be too low to be assessed experimentally or from epidemiological data. In these cases, the onset of promotional effects may be assessed and quantified by suitable biochemical parameters (including suitable toxicogenomics data, see part A "Fundamental considerations", chapter “Background DNA lesions in rodent and human tissues/body fluids”). Regarding Group 3, i.e. compounds that increase DNA damage and mutagenicity via indirect mechanisms, for example by inactivation of DNA repair processes, in principle NOAELs (i.e. thresholds) exist in the absence of additional direct genotoxic activity, since the effects are due to interactions with proteins and/or induction of ROS. Nevertheless, this mode of action deserves special attention since unlike many other promoting effects the relevant interactions may occur at particularly low concentrations. This could provoke repair-deficient conditions which also lead to an accumulation of endogenous DNA damage as well as DNA lesions induced by exogenous sources and, thus, may considerably increase the mutation frequencies per cell division. This is the case of many metal compounds, as detailed in part B "Selected examples" for cadmium and arsenic (see chapter Sec. "Carcinogenic metal compounds: Examples cadmium and arsenic"). Since DNA repair inhibition would result in increased mutagenicity and clastogenicity, the absence of elevated levels of DNA damage, mutations and/or chromosomal damage in vivo under steady-state conditions may serve to quantify the no-effect concentrations as a basis for a limit value setting. Another example would be the induction of genome mutations following the disturbance of the spindle function (aneugenic effects).

Below the concentration at which additional promotional events occur, i.e. in the low dose Range A (Fig. 1), genotoxicity needs to be evaluated quantitatively for all groups in a similar manner. In most cases, since the tumour incidence within range A is too low to be determined experimentally, only an upper limit for the additional tumour risk can be estimated from the data (population and animal studies). However, the ability to exactly measure DNA lesions in tissues and body fluids has remarkably increased in recent years (see part A Fundamental considerations, chapter "Background DNA lesions in rodent and human tissues/body fluids"). In the case of a continuous exposure to a given genotoxic agent (steady-state conditions) in vivo a linear correlation between the induced DNA damage and the additional cancer incidence can be anticipated for range A in Fig. 1 (part A "Fundamental considerations"), in which additional promotional effects of the genotoxic agent are absent by definition. Therefore, the quantification of DNA lesions in target or surrogate cells in vivo is the next step in the suggested strategy shown in Fig. 20. Quantification of surrogate endpoints such as protein adducts might provide additional information (see part A "Fundamental considerations", chapter “Protein adducts as human biomarkers”). Subsequently, two cases (types of genotoxic agents) should be distinguished. If background levels of the same or very similar DNA lesions which can be ascribed to an unavoidable exposure to the same or similar compound, e.g. from endogenous sources, are detectable in the organism/tissue, the (extrapolated) level of DNA damage generated at a certain low level of exposure to the xenobiotic agent can be judged as irrelevant by comparison with the background levels: The relative increase in the cancer incidence can be estimated to be irrelevant, even if the actual absolute risk emanating from this type of DNA lesion is not known. A prerequisite for such a relative risk assessment is the reliable knowledge of the background level of relevant DNA lesions in target/reference populations. Examples for this type of genotoxic agents are ethanol, acetaldehyde and ethylene oxide, among others (see below).

For compounds without comparable background lesions such as benzo[a]pyrene or aflatoxin B1, the next task would be to evaluate the carcinogenic risk at concentrations within range A. If this risk were quantifiable, cancer risk in the low dose range could be assessed according to the slope in the range A in Fig. 1. However, in most cases this will not be possible experimentally; and a worst-case scenario (maximum slope calculated for the cancer incidence in range A, estimated from the onset of range B), might be applied. Furthermore, in many cases data on induced mutations in the low dose range in vivo are not (yet) available and/or not readily assessable, in part due to the lack of sensitive in vivo mutagenicity tests. In such cases, DNA lesions may be quantified in vivo (preferably in the target tissue), thereby providing quantitative data on the onset of measurable DNA damage in vivo. The quantification of steady-state levels of specific DNA lesions, determined after repeated exposure, provides an integrated measure of genotoxicity, which also accounts for the influence of detoxification and DNA repair.

In cases in which the carcinogenic risk associated with a specific lesion is known, these data could be used for quantitative risk estimation. To this end, for example, experiments with animals deficient in the specific repair mechanism of the respective DNA lesion may provide important further information. Such a defect can result in a several-fold increase of the respective lesion without confounding promotional effects. If an increase of cancer incidence becomes observable in the animals under these conditions, the correlation between the DNA lesion levels and the cancer incidence—and thus the carcinogenic potential of the lesions—can be experimentally established. In this case, a reliable risk estimation based on the determination of DNA lesions appears possible.

### Application of the proposed strategy to selected examples

In the following paragraphs, the present situation regarding the outlined strategy is exemplified for several genotoxic agents described in part B "Selected examples"

DNA lesions for which an endogenous background has been described comprise those emanating from the exposure to acetaldehyde/ethanol, ethylene oxide, formaldehyde and further endogenous alkylating agents, such as acrylamide (see part B "Selected examples", chapter "Presence of endogenous background levels of the same or similar DNA lesions").

For example, for **ethanol and acetaldehyde**, which are endogenous substances mainly originating from the intermediate metabolism, a dose-dependent increase in DNA adduct levels induced by acetaldehyde in vitro and in vivo was observed in several studies. There is substantial inter-individual variability with regard to the endogenous background of these adducts in humans, reported to range from about 13–150 adducts/10^8^ nucleotides. The biological significance of the identified adducts with respect to the mutagenicity and carcinogenicity of ethanol and acetaldehyde remains unclear. However, an additional intake from exogenous sources that remains within the range of variation of the endogenous body burden will only contribute to a limited extent to cancer risk. Of note, the comparison to background levels requires to consider all tissues and body compartments of relevance, especially including the sites of first contact, such as the respiratory tract upon inhalation or the upper gastrointestinal tract upon oral exposure. In addition to the induction of DNA damage, acetaldehyde is very reactive, giving rise to irritation and, thus, leading to an additional promotional activity, which may need special consideration (see part B "Selected examples", chapter "Acetaldehyde and Ethanol").

**Formaldehyde** has been classified as carcinogenic, inducing nasal tumours with a sublinear dose–response relationship upon inhalation. While IARC assigned it to group 1 (human carcinogen), the German MAK Commission established a MAK value which protects from formaldehyde carcinogenicity (carcinogen category 4). The induction of different types of DNA damage as well as an increased cell proliferation are considered key events. On the systemic level, DNA damage equivalent to the extent exerted by the endogenous formaldehyde burden is not induced until an inhalative exposure of about 100 ppm is reached. Since the critical effect of formaldehyde is the induction of nasal tumours, the crucial question is at which concentrations formaldehyde reaches the basal cells (target cells for nasal tumours). In addition to DNA damage, the promotional effect of cell proliferation appears required to convert DNA damage into mutations. Since formaldehyde is a very reactive irritant, irritation is most critical, becoming evident far below exposure levels that increase the systemic formaldehyde background levels. With the currently available data it can be assumed that there is no additional risk of nasal tumours at low dose levels (≤ 0.3 ppm) (see part B "Selected examples", chapter Sec. “Formaldehyde”).

In the case of **acrylamide,** the key event resulting in genotoxicity has long been considered to be the formation of glycidamide (GA) and its interaction with DNA. However, GA, preferentially inducing N7-GA-guanine adducts, has been found to be a mutagen of rather low potency. This may be reconciled, at least in part, with the fact that it preferentially alkylates DNA at N7-guanine, an adduct of very low mutagenic potential. Compared to the background range of a spectrum of human tissue DNA adducts determined by advanced HPLC–ESI–MS/MS methodology, N7-GA-guanine levels measured in rats at single dosages up to at least 100 μg/kg bw were found to occur sporadically, not dose dependently and close to the low bound of human background levels of similar N7-guanine adducts. Toxicogenomic response profiles in rodent target tissues, appear to indicate a non-genotoxic MOA rather than a genotoxic MOA. Taken together, the current weight of evidence supports a non-genotoxic MOA, and genotoxicity may only occur at unrealistically high dosage. If this conclusion is further confirmed, it may allow to establish a non-observed adverse effect level as a POD for risk assessment.

In the case of **alkylating agents (MMS, EMS, MNU and ENU)**, which induce genotoxic effects via direct covalent binding to DNA, the quantification of mutagenicity endpoints in vitro and in vivo (lacZ, pig-a, micronuclei) in parallel with that of haemoglobin adducts in vivo support the notion that significant mutagenicity is only observable under conditions at which the relevant DNA repair mechanisms (in these cases mediated by MGMT) are saturated (see part B "Selected examples", chapter Sec. “Alkylating agents”). This may be true even for cases in which the dose-dependence appears linear in the concentration range tested, because non-saturating concentrations were not applied (as for ENU in the lacZ and micronucleus tests, in contrast to the results in the pig-a test, in which lower, non-saturating concentrations were also tested). In other words, the available data allow to define the onset of the range B in Fig. 1, which in this case is characterized by a saturation of repair mechanisms. The remaining question is whether an upper limit for the mutagenicity (and in the next step cancer risk) in the range below this threshold concentration (i.e. in range A) can be estimated, making use of the known sensitivities of the mutagenicity assays applied and considering that the endogenous background levels of N7-alkyl-guanine adducts have been reported to be in the range of about 29–44 adducts/10^8^ nucleotides. As suggested in the general strategy outlined above (Fig. 20), such a calculation appears more appropriate than either the assumption of a zero risk below this threshold concentration or the linear extrapolation of the range B data (which would clearly result in a high over-estimation of the risk).

**Ethylene oxide** is endogenously formed due to CYP-dependent ethylene oxidation, mainly driven by CYP2E1. Ethylene is an endogenous metabolite in part generated by the gut microbiome and is also taken up from external sources, such as food. However, a quantitative risk estimation based on the DNA adduct levels appears not to be suited, since the levels of the different adducts formed vary in different organs, and their formation is not solely based on ethylene oxide. Therefore, the endogenous formation rate may better be extrapolated from the haemoglobin adduct frequency in unexposed human individuals by linear regression (see part B "Selected examples", chapter Sec. “Ethylene oxide”). An increment of approximately 5 nmoles hydroxyethyl valine per gram haemoglobin corresponds to an average additional inhalative workplace exposure (40 h per week) of 1 ppm in the air. Compared to the average background of 20 pmol hydroxyethyl valine per gram haemoglobin in non-exposed healthy non-smokers, this increase by almost three orders of magnitudes per ppm qualifies this lesion as a particularly sensitive marker of exposure. This background value suggests the ethylene oxide exposure from endogenous sources to be at best in a range equivalent to a workplace exposure of 0.004 ppm and should represent a lifetime tumour risk increase of far less than 1 in 100,000.

Several compounds have been discussed in part B "Selected examples" which show a linear relationship between the dose and the level of DNA adducts. For example, there is a linear relationship between the administered **Aflatoxin B1** (AFB1) dose and the extent of AFB1-DNA binding (i.e. amount of AFB1-DNA adducts formed) in rats and rainbow trout, and no threshold for the formation of hepatic DNA adducts appears to exist. This means that tumour promoting effects are either absent or present at all concentrations to an extent that is proportional to the aflatoxin concentration and DNA adduct formation. In both cases, risk estimation based on the linear extrapolation of adduct levels appears possible. The linearity of the data allows to calculate that in rats only 53 aflatoxin-DNA adducts per 10^8^ nucleotides are required to induce a 50% tumour incidence, thereby indicating that this is a very potent DNA lesion with respect to mutagenicity and carcinogenicity (Otteneder and Lutz 1999). Of note, the AFB1-DNA adduct in humans is often causing an inactivating point mutation in codon 249 of the p53 tumour suppressor gene according to sequencing data from population studies. Since an analogous p53 inactivation is not observed in rats, the finding is an indication that the potency of a given type of point mutation to cause tumours is species-dependent (see part B "Selected examples", chapter Sec. “Aflatoxin B1”). For carcinogenicity in humans, it is to be noticed however that an obvious and marked influence of promotional effects is exerted by HBV infection.

There is also a linear relationship through the origin between the dose of an **allylalkoxybenzene** and its level of bioactivation to the DNA reactive metabolite as well as the level of DNA adduct formation (see part B "Selected examples", chapter Sec. “Allylalkoxybenzenes”). This linearity holds true from dose levels as high as the BMD_10_ (benchmark dose that leads to a 10% extra cancer incidence) down to dose levels as low as dietary human intake. Physiologically based kinetic (PBK) modelling provides insight into the form of the dose–response curve for the formation of unstable DNA reactive metabolites even at dose levels that are experimentally not accessible. With respect to tumour formation, a role for additional toxicity as a promotional event in addition to the DNA adduct formation may be relevant as well, given that for alkenylbenzenes dose levels causing carcinogenicity are close to or even overlap with those causing increased toxicity. This hampers a quantitative risk assessment for a low-dose range.

**Benzo[a]pyrene** induces various types of DNA damage. In cell culture systems, stable adducts of its reactive metabolite, benzo[a]pyrene diolepoxide (BPDE) at the *N*-2 position of guanine are detectable at a concentration of ≥ 10 nM. Repair occurs via nucleotide excision repair; however, even in the low dose range, it was observed to be not complete. A linear dose–response relationship between DNA adducts induced by the reactive metabolite BPDE and mutations in the same cell line was reported even down to very low concentrations. In contrast, the transcriptional response to DNA damage was restricted to higher concentrations. In consequence, no difference within the relationship between concentrations inducing DNA adducts and mutations was observed, and a linear dose–response relationship with respect to carcinogenicity in the low dose range should be anticipated (see part B "Selected examples", chapter Sec. "Benzo[a]pyrene").

There is a linear correlation between the administered **2-amino-3,8-dimethylimidazo[4,5-f]quinoxaline (MeIQx)** doses and the extent of MeIQx-DNA binding (i.e. amount of MeIQx-DNA adducts formed) in the liver of rats exposed to MeIQx, even in the low dose range (see part B "Selected examples", chapter Sec. “2-Amino-3,8-dimethylimidazo[4,5-f]quinoxaline (MeIQx)”). Thus, the steady-state adduct level of 113 ± 10 attogram/µg DNA was observed at a daily dose of 0.1 µg/kg (Frantz et al. 1995). This dose is only slightly higher than the daily uptake of 0.003–0.043 µg/kg bw/day calculated for humans (Murai et al. 2008). Indications for carcinogenic effects in the rat liver (formation of preneoplastic foci) were obtained at an exposure level of 10 ppm MeIQx in the diet (approx. 400 µg/kg bw/day). Data support the notion that the promoting activity of the compound is very low, but nevertheless involved in the cancer induction observed (which is associated with a high toxicity). The compound, therefore, seems suitable for a quantitative low-dose cancer risk assessment according to the scheme in Fig. 20. However, a putative threshold for the promoting effects of MeIQx (liver cell proliferation) and an upper limit for the cancer incidence directly below this concentration remain to be established.

As described in detail in part B "Selected examples", **Pyrrolizidine alkaloids (PAs)** differ largely not only with respect to their carcinogenic potential but also to the types of DNA adducts formed (e.g. monoadducts and DNA-DNA cross-links). Only limited information on dose–response relationships, especially in the low-dose range is available (see part B "Selected examples", chapter Sec. “Pyrrolizidine alkaloids”). In the case of riddelliine, the levels of DNA adducts were found to correlate closely with the tumorigenic potencies in rats in a dose-dependent manner. Moreover, a linear correlation was observed between the administered riddelliine doses and mutation frequencies in liver *cII* genes of Big Blue transgenic rats. However, no clear linear correlation was observed between the induction of hepatic neoplastic lesions (liver haemangiosarcomas) and the administered riddelliine or lasiocarpine doses, especially in the low-dose range. The potent hepatotoxicity of the compounds makes it difficult to separate genotoxic from tumour promoting effects. This hampers a quantitative risk assessment for the low-dose range.

Another important effect consists in the potential interference of specific agents with the cellular response to DNA damage, thereby decreasing genomic stability. This applies, for example, to **cadmium and arsenic**, which are known human carcinogens, but also to other carcinogenic metal compounds like those of nickel, cobalt and antimony. Both cadmium and arsenic interact with DNA repair processes, cell cycle control and tumour suppressor functions, thus increasing the mutation frequency due to repair inhibition of endogenously generated DNA lesions as well as in combination with other DNA-damaging agents (part B "Selected examples", chapter Sec. “Carcinogenic metal compounds: Examples cadmium and arsenic”). Furthermore, cadmium induces oxidative stress by inactivation of anti-oxidative defense systems, additionally affecting genomic stability. Also, both metals provoke epigenetic alterations. Of note, in the case of cadmium a potential limit value may be based on the interactions with the DNA damage response system. However, the concentration at which cadmium interferes with this system still needs to be elucidated and should be compared to exposure levels causing kidney toxicity (see detailed discussion in part B "Selected examples"). In the case of arsenic, an increased tumour risk is already evident at environmental exposure conditions even in Europe, and thus respective interactions are expected to be relevant already at very low exposure levels. In agreement with this assumption, interactions with DNA repair systems such as poly(ADP-ribosyl)ation have been observed at extremely low concentrations in vitro. This allows the conclusion that meaningful health-based limit values would need to be below current environmental exposure. (for a detailed discussion see part B "Selected examples"). At present, therefore, the best option is to minimize exposure.

### Gaps in knowledge and research needs

The Senate Commissions MAK and SKLM recommend the advancement of science-based limit values for tolerable exposure to (genotoxic) carcinogens. Therefore, the exploration of key events relevant to the mode of action of genotoxic carcinogens, with specific consideration of DNA damage induction, dose–response relationships, DNA repair and mutagenic potential is proposed. To that end the following research needs have been consented:

- Exploration and validation of dependable (surrogate) biomarkers to monitor endogenous and exogenous exposure to genotoxic agents in populations living under different nutritional, lifestyle, socioeconomic and working conditions.
- Determination of human background DNA damage in these populations and establishment of an open-access comprehensive database on DNA background damage.
- Establishment of standard reference methodology for (in vivo) mutagenicity assays and exploration of the correlation between in vivo DNA damage and in vivo mutation induction, case by case for individual genotoxic agents and for various types of DNA damage in different tissues.
- Systematic grouping of DNA lesions into classes according to chemical structure and mutagenic potency to allow for read-across estimates within similarity classes.
- Development of lifetime cancer risk estimates associated with the various types of DNA damage and induced mutations in different tissues.
- Mechanistic understanding of (additional) promotional and indirect mechanisms of genotoxicity and establishment of thresholds for these activities.
- Evaluation of the merit of novel methodology (target tissue to single-cell toxicogenomics, advanced PBK and in silico methods) to comprehensively inform about biomolecular key events driving adverse outcome pathways.

## Definitions/Glossary

DNA damage: chemical modification of DNA without physiological function

Mutation: heritable change of the genome of a cell

Mutagenicity: capability to increase the number of mutations per cell division

Genotoxicity: capability to cause cellular DNA damage and/or increase the number of mutations per cell division

Indirect genotoxicity: capability to increase the number of mutations per cell division without direct DNA interaction

Promoting activity: capability to increase the tumour frequency without genotoxicity

Carcinogenicity: capability to increase the tumour frequency

## Footnotes

1 BMDL_10_ = benchmark dose lower confidence limit for a benchmark response of 10%
